# Isostructural Halogen Exchange and Halogen Bonds: The Case of *N*-(4-Halogenobenzyl)-3-halogenopyridinium Halogenides

**DOI:** 10.1021/acs.cgd.1c01285

**Published:** 2022-01-05

**Authors:** Luka Fotović, Nikola Bedeković, Vladimir Stilinović

**Affiliations:** Department of Chemistry, Faculty of Science, University of Zagreb, Horvatovac 102a, 10000 Zagreb, Croatia

## Abstract

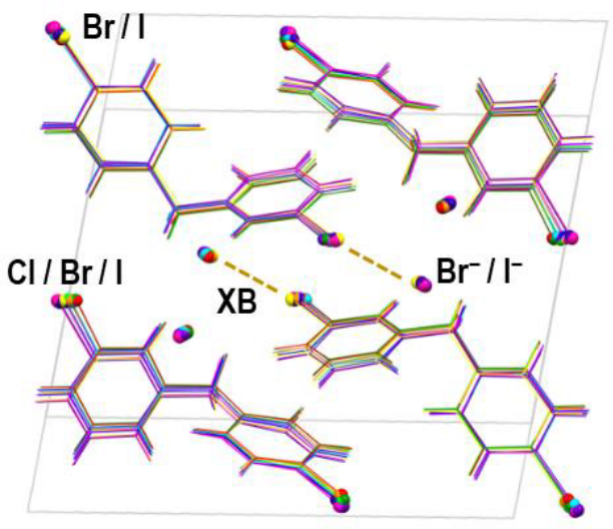

Six *N*-(4-halogenobenzyl)-3-halogenopyridinium cations were prepared by reacting *meta*-halogenopyridines (Cl, Br, and I) with (4-halogenobenzyl) bromides (Br and I) and were isolated as bromide salts, which were further used to obtain iodides and chlorides. Sixteen compounds (out of 18 possible cation/anion combinations) were obtained; two crystallized as hydrates and 14 as solvent free salts, 11 of which belonged to one isostructural series and 3 to another. All crystal structures comprise halogen-bonded chains, with the anion as an acceptor of two halogen bonds, with the pyridine and the benzyl halogen substituents of two neighboring cations. The halogen bonds with the pyridine halogen show a linear correlation between the relative halogen bond length and angle, which primarily depend on the donor halogen. The parameters of the other halogen bonds vary with all three halogens, indicating that the former halogen bond is the dominant interaction. This is also in accord with the calculated electrostatic potential in the σ-holes of the halogens and the thermal properties of the solids. The second isostructural group comprises combinations of the best halogen bond donors and acceptors, and features a more favorable halogen bond geometry of the dominant halogen bond, reaffirming its significance as the main factor in determining the structure.

## Abstract 2

Halogen atom exchange in *N*-(4-halogenobenzyl)-3-halogenopyridinium halogenides yielded two series of isostructural salts which differ in halogen bonding and properties.

## Introduction

The goal of crystal engineering is the deliberate design of crystals with planned structures and predictable physical and chemical properties.^1−6^ In attempting to achieve this, one must take into account both the geometric properties of the constituent molecules, as well as their proclivity to participate in intermolecular interactions. The intricate interplay of these two effects is perhaps best illustrated by two phenomena: the ability of one substance to crystallize in more the one crystal structure—polymorphism^7−11^—and the occurrence of different substances adopting very similar (or almost identical) crystal structures—isostructurality.^12−16^ Of these two phenomena, isostructurality is particularly useful for the study of minute differences in crystal structures and properties, as in isostructural materials the majority of various contributions of the overall crystal packing can be taken to be equivalent, leaving the differences between the constituent molecules as the main cause of any variability between the structures and the properties within an isostructural series.^17−25^

One field of solid state of supramolecular chemistry which has particularly benefited from the study of isostructural systems is the study of the halogen-bonded materials.^26−34^ The reason for this is that replacing one halogen atom with another in a molecule generally has only a minute effect on molecular geometry. It was already noted by Kitaigorodsky in his seminal *Organic Crystallochemistry*(35) that replacing one halogen atom with its neighbor in the group (Cl/Br or Br/I) will in roughly 50% of cases lead to isostructural materials. As the halogen bond energy greatly increases with the donor atom size,^36^ in such isostructural crystals (providing the halogen atom does act as a halogen bond donor), the only significant difference between the two crystals will be the strength of the halogen bond. Furthermore, as the only significant difference between two such isostructural crystals lies in the halogen bond strength, all differences in physical and chemical properties are also mainly due to the difference in halogen bond energies. This has been employed for experimental observations of the effect of the halogen bond on the macroscopic properties of isostructural halogen-bonded materials, as well as fine-tuning of their properties.^37,38^ Unfortunately, this approach does have its limitations: while isomorphous dual exchange Cl/Br and Br/I is a fairly common phenomenon,^17−19^ triple isomorphous exchange Cl/Br/I is quite rare. To the best of our knowledge, there have been to date only eight published systems with triple isomorphous exchange^30,32,39−44^ involving halogen which acts as a halogen bond donor.

The tunability of halogen bond strength within a set of isostructural crystals can be increased if the acceptor atom can also be replaced without changing the overall structure of the crystal. One method for systematic application of this principle is using halogen atoms not only as donors but also as halogen bond acceptors. This can be achieved in several ways; halogen atoms can act as halogen bond acceptors either of part of the neutral molecule (type II XB) or as halogenide anions (in their “free” form or coordinated as ligands to metal centers). The latter approach was very successfully employed by the Brammer group for the study of the hierarchy of intermolecular interactions in 3-halogenopyridinium tetrahalogenometalates, demonstrating that the structure type is dependent on both the hydrogen and the halogen bond strength.^37^ Also, halogenopyridinium halogenides were shown to be extremely prone to isostructurality: among both the *ortho*- and *para*-halogenopyridinium halogenides (halogen = Cl, Br, I), there are groups of six isostructural salts, while among *meta*-halogenopyridinium halogenides there are two groups of four.^45−48^

In the present work, we are describing the design, preparation, and study of a series of *N*-(4-halogenobenzyl)-3-halogenopyridinium halogenides. The cation was selected as a potential donor of two inequivalent halogen bonds—one through the halogen on the pyridine ring (on which the majority of the charge is expectedly located) and the other through the halogen on the *N*-benzyl substituent. Using a halogenide counterion as a halogen-bond acceptor gives a total of three halogen atoms (two on the cation and one of the anion) which can be interchanged in order to investigate in which cases the exchange of the halogen will induce a change in the structure and when it will yield an isostructural solid. Additionally, as we are using a relatively large cation (comprising two rings), the exchange of halogen substituents will only lead to small differences in the overall molecular volume, which should lead to a higher probability of obtaining isostructural crystals.^49^ This would enable us to study in more detail how halogen bond affects the structures and properties of the crystals.

## Results and Discussion

**Scheme 1:**
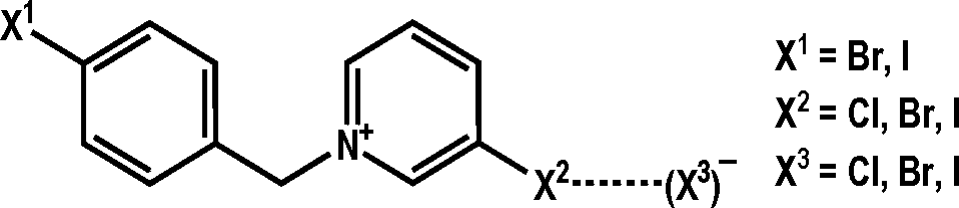
Halogen-Bonded Cation–Anion Pair with Annotation of the Three Halogen Atoms

The cations were prepared by reacting *meta*-halogenopyridines (Cl, Br, and I) with (4-halogenobenzyl) bromides (Br and I), which yielded a series of six bromide salts of *N*-(4-halogenobenzyl)-3-halogenopyridinium cations. Iodides and chlorides were prepared from the bromides by ion exchange, giving an overall potential of 18 compounds which differ only in one or more halogen atoms (Scheme 1). For the sake of simplicity, these will be referred throughout the text as **X**^**1**^**X**^**2**^**X**^**3**^, where **X**^**1**^ is the halogen substituent on the benzyl ring, **X**^**2**^ the substituent on the pyridine ring, and **X**^**3**^ the halogenide anion.

All the bromides and iodides crystallized as simple 1:1 salts with no inclusion of solvent molecules. In the case of chlorides, however, the outcome of the synthesis was found to depend on the halogen substituent on the pyridine ring. The two cations derived from 3-iodopyridine thus yielded simple chloride salts equivalent to the iodides and the bromides. When bromine replaced the iodine atom on the pyridine ring, the chlorides crystallized as hydrates (**IBrCl**·H_2_O and **BrBrCl**·1.5H_2_O), while the two chlorides of the cations derived from 3-chloropyridine could not be isolated. Thus, out of the 18 possible **X**^**1**^**X**^**2**^**X**^**3**^ combinations, a total of 16 were obtained: 14 as simple salts, and two as hydrates.

**Figure 1:**
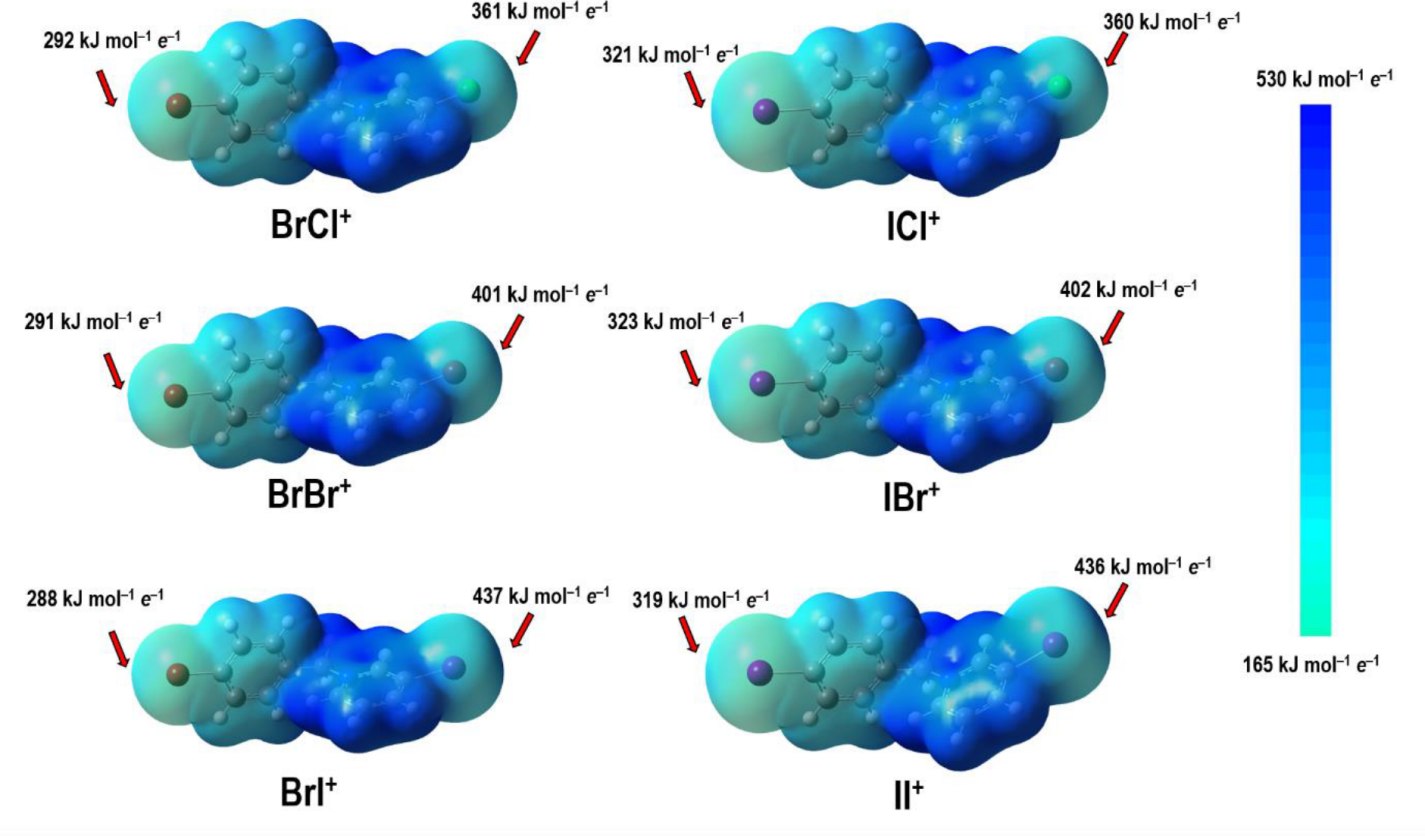
Electrostatic potential plotted on a 0.002 *e* Å^–3^ electron density isosurface for the six cations covered by this study, with values of ESP on the halogen σ-holes (*V*_max_) for **X**^**1**^ and **X**^**2**^.

In order to evaluate the potential of the halogen atoms on the cations for formation of halogen bonds, we performed DFT computations of the electrostatic potential (ESP) of the cations *in vacuo*. These have shown that the halogen substituent on the pyridine ring in all cases has a more positive σ-hole ESP (*V*_max_) than the benzyl substituent: for the halogen on the pyridine ring, *V*_max_(**X**^**2**^) decreases from iodine (ca. 435 kJ mol^–1^*e*^–1^) over bromine (ca. 400 kJ mol^–1^*e*^–1^) to chlorine (ca. 360 kJ mol^–1^*e*^–1^), whereas for the benzyl halogenides *V*_max_(**X**^**1**^) is ca. 320 kJ mol^–1^*e*^–1^ for iodine and ca. 290 kJ mol^–1^*e*^–1^ for bromine (Figure 1). It is therefore evident that the pyridine halogen atom is expected to form stronger halogen bonds, which will therefore (expectedly) be the dominant contribution in determining the properties of the materials. Indeed, this seems to be illustrated by the synthesis of the chlorides. As chloride is the best hydrogen bond acceptor of the three anions, only the strongest halogen bond donors (iodopyridinium cations) can entirely replace the water molecules which solvate the chloride in solution. Weaker halogen bond donors (bromopyridinium cations) do replace some of the solvent water, giving hydrates in which the chloride is an acceptor of a halogen bond and several HO–H···Cl^–^ hydrogen bonds. However, when only the weakest halogen bond donors (chloropyridinium cations) are present, the chloride remains entirely hydrated, rendering the salt extremely soluble (and probably hygroscopic and deliquescent), which explains our failure to obtain solid products.

**Figure 2:**
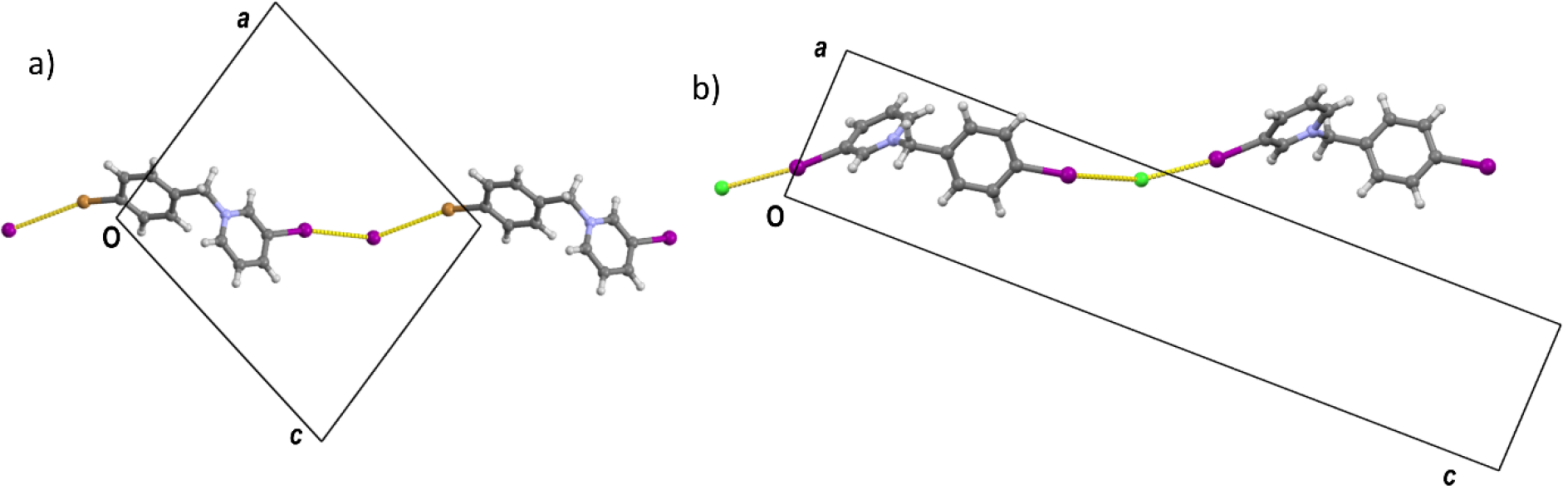
Halogen-bonded chains within the unit cell in (a) type I structures (in **BrII**) and (b) type II structures (in **IICl**). Both structures viewed along the crystallographic *b* axis.

Out of the 14 anhydrous salts, 11 belong to one isostructural series (type I) and the remaining three to another (type II). The first isostructural series comprises the iodide salts of all six cations and five bromides, while the second comprises the two chlorides and the remaining bromide (**IIBr**). The crystal structures of both series contain halogen-bonded chains, with cations and anions interconnected by halogen bonds (Figure 2, Figure S35 in the Supporting Information); each anion is an acceptor of two halogen bonds, one with the pyridine halogen atom (**X**^**2**^···(**X**^**3**^)^−^), and one with the benzyl halogen atom (**X**^**1**^···(**X**^**3**^)^−^). Along with the two halogen bonds, the halogenide anion is also an acceptor of several weak C–H···(**X**^**3**^)^−^ hydrogen bonding contacts with cations belonging to neighboring halogen-bonded chains (see Table S3 in the Supporting Information). These interactions interconnect the halogen-bonded chains into a 3D structure.

### Isostructurality within the Type I Structures

In order to quantify the similarity of the 11 structures belonging to type I, we have calculated the unit cell similarity indices:
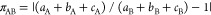
where *a*_A_, *b*_A_, and *c*_A_ are orthogonalized cell parameters for structure A, and *a*_B_, *b*_B_ and *c*_B_ are orthogonalized cell parameters for structure B, as well as the isostructurality indices:
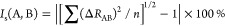
where Δ*R*_AB_ is the difference of distances of atomic coordinates of equivalent atoms in structures A and B, and *n* is the number of atoms in the section of the structure which is being compared for each pair of the structures.^13^ For computation of *I*_s_, all non-hydrogen atoms in the unit cell were taken into account (*n* = 56). The values of π_AB_ and *I*_s_(A,B) for the 11 crystals belonging to the structural type I are given in Table 1.

**Table 1:**
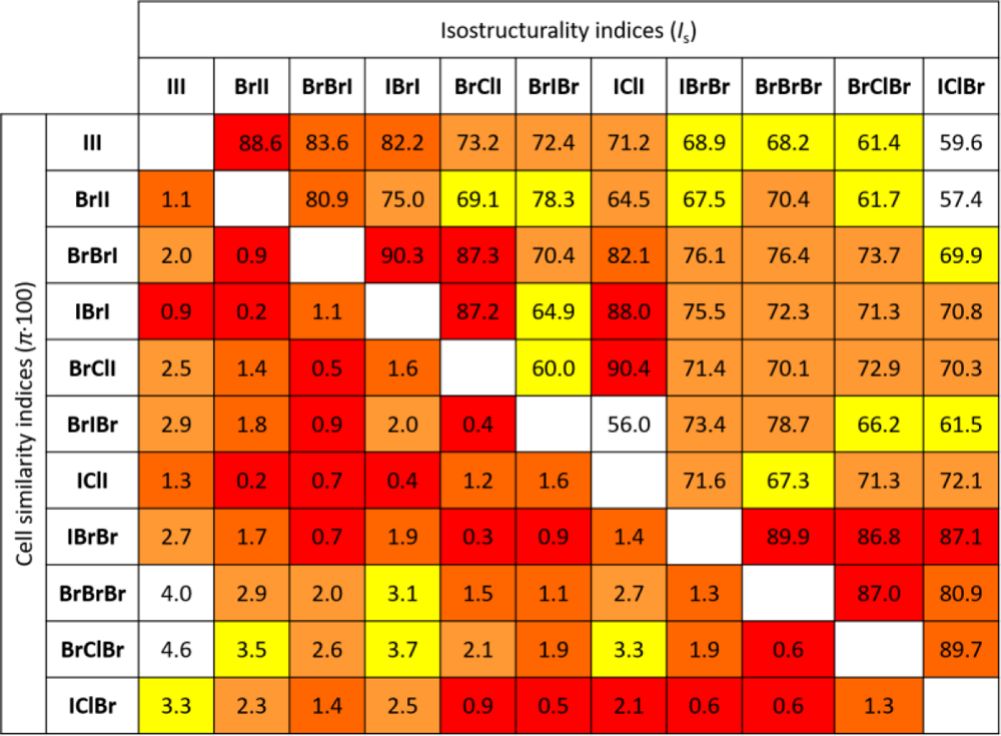
Unit Cell Similarity (π_AB_) and Isostructurality Indices (*I*_s_(A,B)) for the 11 Crystals Belonging to Structural Type I^a^

a The isostructurality indices have been computed taking into account all non-hydrogen atoms in the unit cell (*n* = 56).

Expectedly, the most similar structures (with *I*_s_ values above 85%) are generally those where only a single Cl/Br or Br/I substitution has occurred, although there are two highly isostructural pairs which differ in two halogen atoms (**IBrI**/**BrClI** with *I*_s_ of 87.2% and **IBrBr**/**BrClBr** with *I*_s_ of 86.8%). It should be noted that the structures are generally less sensitive to replacement of the halogen atoms of the cation (all of the above-mentioned pairs differ only in **X**^**1**^ or/and in **X**^**2**^) than the anion. This is most probably due to the larger differences in the radii of the halogenide anions. On the other hand, while the same observations generally hold for overall cell similarities, some of the isostructural pairs with the most similar cells do differ in the anion (**BrIBr**/**BrClI** with π = 0.004 and **IClBr**/**BrClI** with π = 0.009). Also, some pairs with the lowest π_AB_ feature Cl/I exchange (the above-mentioned **BrIBr**/**BrClI**, **IClI**/**BrII** with π = 0.002 and **IClBr**/**BrIBr** with π = 0.005). It is interesting to note that the Cl/I exchange with the least effect on cell similarity occurs only in **X**^**2**^ and is always in conjunction with a second (Br/I) exchange so that the large increase in the size of **X**^**2**^ is apparently somewhat compensated by a decrease in **X**^**1**^ or **X**^**3**^ and consequently does not significantly affect the overall unit cell dimensions (although it does lead to an overall change in atom positions, as evidenced by lower corresponding isostructurality indices: *I*_s_(**BrIBr**/**BrClI**) = 60.0%; *I*_s_(**IClI**/**BrII**) = 64.5% and *I*_s_(**IClBr**/**BrIBr**) = 61.5%).

**Figure 3:**
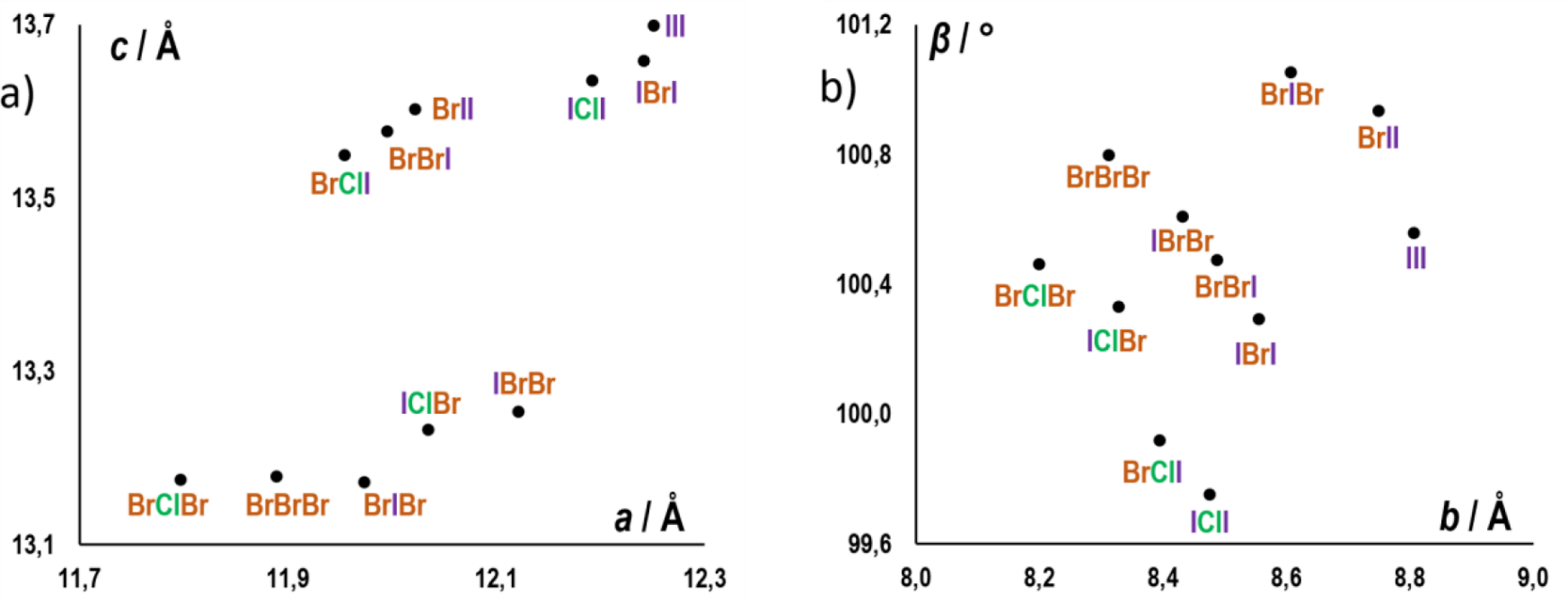
Unit cell parameters of type I structures (a) *c* vs *a* plot, (b) *β* vs *b* plot.

Generally, unit cell parameters within the type I series change fairly little—there is only ca. 4% difference between the maximum and minimum *a* and *c* lengths, ca. 7.5% between the maximum and minimum *b* lengths, and mere 1% between the maximum and minimum β angles. All the unit cell parameters are somewhat affected by the nature of all three halogens. The unit cell vector lengths (*a*, *b*, and *c*) generally increase with the size of all three halogens, while β generally increases with **X**^**2**^ and decreases with **X**^**1**^ and **X**^**3**^. However, apart from the cell angle which appears to be affected by all three halogens to a more-or-less equal measure, *a*, *b*, and *c* cell parameters depend more on some of the halogens. Thus, *a* is mostly affected by **X**^**1**^ and to a lesser extent by **X**^**2**^ and **X**^**3**^, with bromides more sensitive to the changes of **X**^**1**^ than the iodides. Conversely, *c* is primarily dependent on **X**^**3**^ and to a smaller degree on **X**^**1**^; among the bromides, *c* is almost entirely independent of **X**^**2**^, but among the iodides, *c* slightly increases with the size of **X**^**2**^. The change of **X**^**2**^ has by far the largest effect on the length of *b*, as well as on the unit cell angle (Figure 3).

**Scheme 2:**
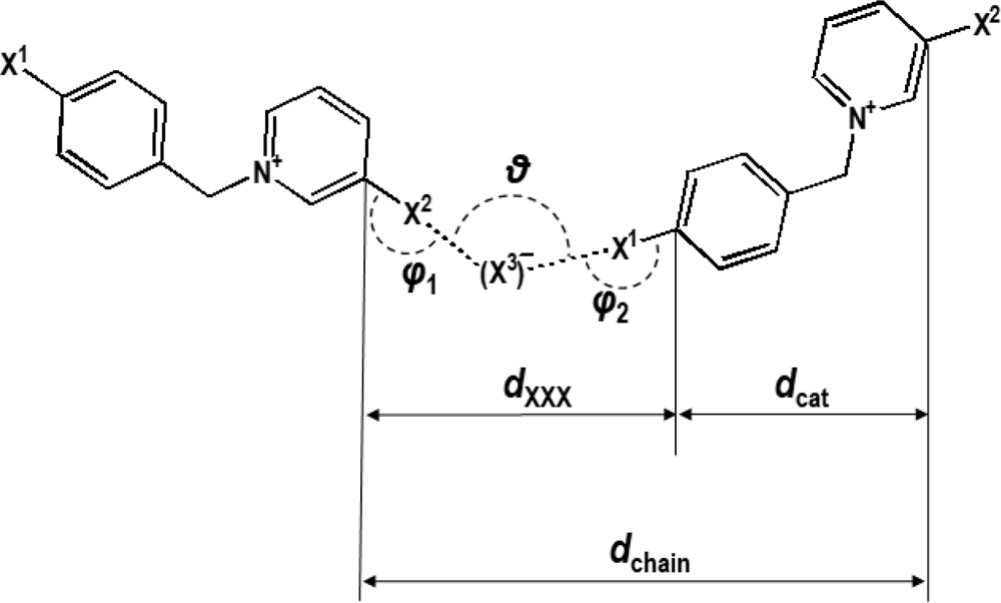
Definition of the Parameters Describing the Halogen-Bonded Chains in Type I and Type II Structures

As the halogen-bonded chains in type I structures extend along the <101> direction (Figure 2c), the increase in *a* and *c* cell parameters, as well as the decrease of β, all correspond to an increase in the overall length of the period of the chain (total length of a cation–anion unit, *d*_chain_, which in type I structures corresponds to the lattice period in the <101> direction, *d*(101) = [*a*^2^ + *c*^2^ + 2*ac* cos β]^1/2^; Scheme 2). Therefore, the increase of *a* and *c* with the size of all three halogens is to be expected, as the increase of the radius of either of the halogens is bound to lead to longer chains. However, the increase of β with the increase of the size (and halogen-bond donor strength) of **X**^**2**^, implies an effect of the halogen bond, rather than simply the radius of the halogen atom on the unit cell.

**Figure 4:**
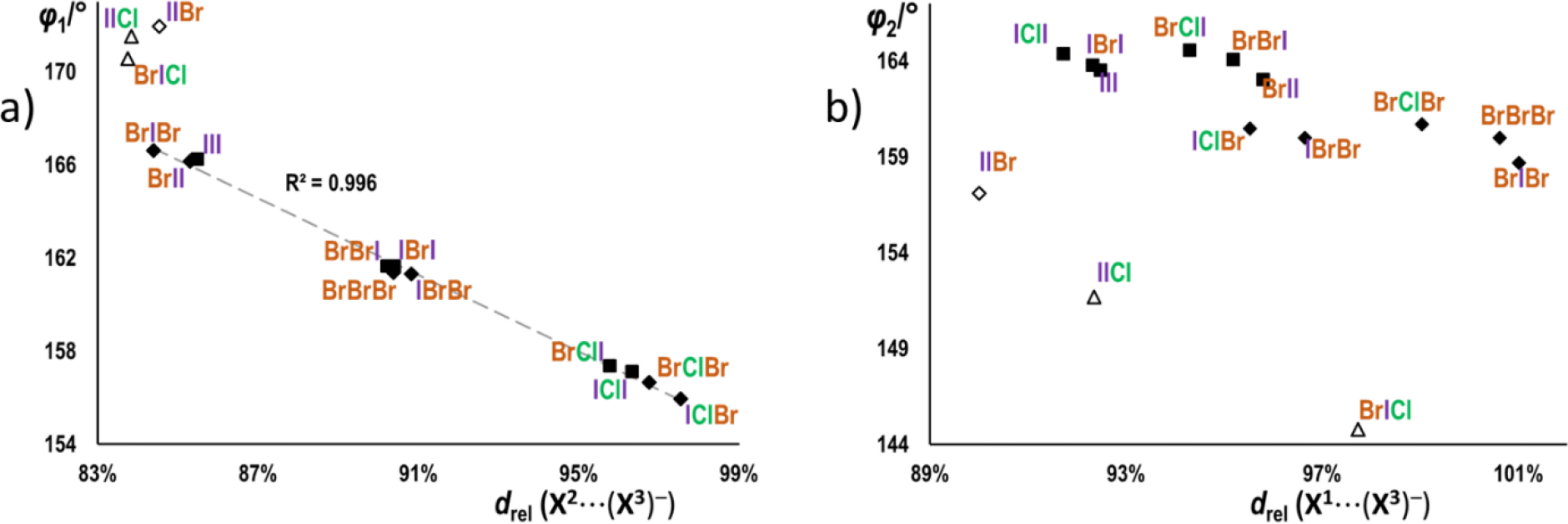
Correlation between the relative halogen bond length (*d*_rel_ = *d*(**X**···(**X**^**3**^)^−^)/[*r*_vdW_(**X**) + *r*_p_((**X**^**3**^)^−^) and the halogen bond angle (φ) for the (a) halogen bond with the donor atom on the pyridine ring (C2–**X**^**2**^···(**X**^**3**^)^−^) and (b) on the benzyl ring (C2–**X**^**1**^···(**X**^**3**^)^−^). Type I structures are represented by black circles, and type II structures are represented with white circles with a black border.

There is quite a significant difference in halogen bond donor and -acceptor properties when Cl/Br/I substitution occurs. Cations and anions are interconnected into chains by two inequivalent halogen bonds, the strength of which should increase with the increase of **X**^**1**^ and **X**^**2**^, (**II**^+^ being the expectedly best donor) and with the decrease of **X**^**3**^ (with Cl^–^ as the best acceptor). In the case of the halogen bond involving the pyridine halogen (**X**^**2**^···(**X**^**3**^)^−^), there is an almost perfectly linear correlation (*R*^2^ = 0.996) between the relative halogen bond length (*d*_rel_ = *d*(**X**···(**X**^**3**^)^−^)/[*r*_vdW_(**X**) + *r*_p_((**X**^**3**^)^−^)] (where is *r*_p_ the Pauling ionic radius^50^)) and the halogen bond angle. Both primarily depend on **X**^**2**^ (angles increasing from ca. 156° for **X**^**2**^ = Cl, over ca. 161° for **X**^**2**^ = Br to ca. 166° for **X**^**2**^ = I, and the relative lengths from ca. 96% for **X**^**2**^ = Cl, over ca. 90% for **X**^**2**^ = Br to ca. 85% for **X**^**2**^ = I). The nature of the acceptor has much less effect on the halogen bond angle, the bonds involving bromide as an acceptor being slightly more contracted than those involving iodide (Figure 4a). The nature of **X**^**1**^ on the other hand has very little effect on either the length or the angle of the **X**^**2**^···(**X**^**3**^)^−^ halogen bond, except in the **X**^**1**^**ClX**^**3**^ series, where **BrClI** and **BrClBr** form somewhat shorter and more linear **X**^**2**^···(**X**^**3**^)^−^ bonds than their respective 4-iodobenzyl analogues (**IClI** and **IClBr**).

The halogen bonds involving the benzyl halogen (**X**^**1**^···(**X**^**3**^)^−^) tend to be somewhat more linear (halogen bond angles mostly in the ca. 159–165° range) but relatively longer (*d*_rel_ = in the 92–101% range). The interdependence of the bond angles versus relative lengths follows the same general trend as in the case of the **X**^**2**^···(**X**^**3**^)^−^ bond, albeit with a considerably larger scatter of the data points (Figure 4b) and dependence on **X**^**3**^ and **X**^**2**^. The structures with the stronger donor atom (**X**^**1**^ = I) generally exhibit relatively shorter and more linear bonds than those with (**X**^**1**^ = Br). Conversely, the structures with the stronger acceptor (**X**^**3**^ = Br) are generally longer and less linear than those with **X**^**3**^ = I. There is also a significant dependence of the **X**^**1**^···(**X**^**3**^)^−^ halogen-bond parameters on **X**^**2**^—within each group of structures which differ only in **X**^**2**^, the relative length increases, and the angle decreases, in the order **X**^**2**^ = Cl < Br < I.

All of these observations, as well as the (almost) perfect linearity of the *d*_rel_(**X**^**2**^···**X**^**3**^)/φ_1_ plot, indicate that the **X**^**2**^···(**X**^**3**^)^−^ halogen bond is the dominant interaction in the crystal structure—the structural differences within the type I series of structures are primarily dictated by the **X**^**2**^···(**X**^**3**^)^−^ halogen bond, while the geometry of the **X**^**1**^···(**X**^**3**^)^−^ halogen bond is modified so that the crystal packing may better accommodate the difference in the **X**^**2**^···(**X**^**3**^)^−^ halogen bond. Thus, **X**^**2**^···(**X**^**3**^)^−^ halogen bonds with the stronger acceptor anion (bromide) tend to be shorter and more linear than those with the iodide, but **X**^**1**^···(**X**^**3**^)^−^ bonds are longer and less linear since within a **X**^**2**^···(**X**^**3**^)^−^···**X**^**1**^ group the stronger acceptor (bromide) is more drawn to the stronger donor (**X**^**2**^), thus elongating and deforming the **X**^**1**^···(**X**^**3**^)^−^ halogen bond. Also, the change of **X**^**2**^ affects the geometry of **X**^**1**^···(**X**^**3**^)^−^ so that the geometry of the **X**^**1**^···(**X**^**3**^)^−^ halogen bond becomes less favorable as the strength of the halogen bond donor of the **X**^**2**^···(**X**^**3**^)^−^ halogen bond increases, while the **X**^**2**^···(**X**^**3**^)^−^ halogen bond is generally independent of **X**^**1**^.

This dominance of the **X**^**2**^···(**X**^**3**^)^−^ halogen bond is also in accord with the calculated electrostatic potential in the σ-holes of the halogens (*V*_max_(X)) which are in all cases higher for the halogen bonded on the pyridine ring than on the halogen on the benzyl ring (Figure 1). It should be noted, however, that the difference in *V*_max_ for **X**^**2**^ = Cl and **X**^**1**^ = I is relatively small (ca. 12%), which indicates that in the case of chloropyridine derivatives the competition between the benzyl-I···(**X**^**3**^)^−^ halogen bond might be able to somewhat compete with the pyridine-Cl···(**X**^**3**^)^−^ halogen bond. This indeed does seem to be the case, seeing that in the **X**^**1**^**ClX**^**3**^ series, the pyridine-Cl···(**X**^**3**^)^−^ bond parameters are apparently more dependent on the nature of **X**^**1**^, being somewhat longer and less linear when **X**^**1**^ = I, as opposed to **X**^**1**^ = Br (in each pair with identical **X**^**3**^).

Seeing that the **X**^**2**^···(**X**^**3**^)^−^ halogen bond has been demonstrated as the dominant interaction in the type I isostructural series, the obvious question which arises is whether, and in what manner, is the isostructurality within the series dependent on this halogen bond. As a unique descriptor for each structure within the isostructural series, we have decided to use the similarity between a given structure and an arbitrarily selected standard “structure”. As the standard, we have selected **III**. This is because we have calculated the *I*_s_ with respect to the entire contents of the unit cell, and, consequently, *I*_s_ for any pair of structures depends on the difference of the unit cell sizes, and therefore a structure with one of the extreme cell volumes (**BrClBr** with the smallest, or **III** with the largest cell volume) is a logical choice as the standard for comparison.

**Figure 5:**
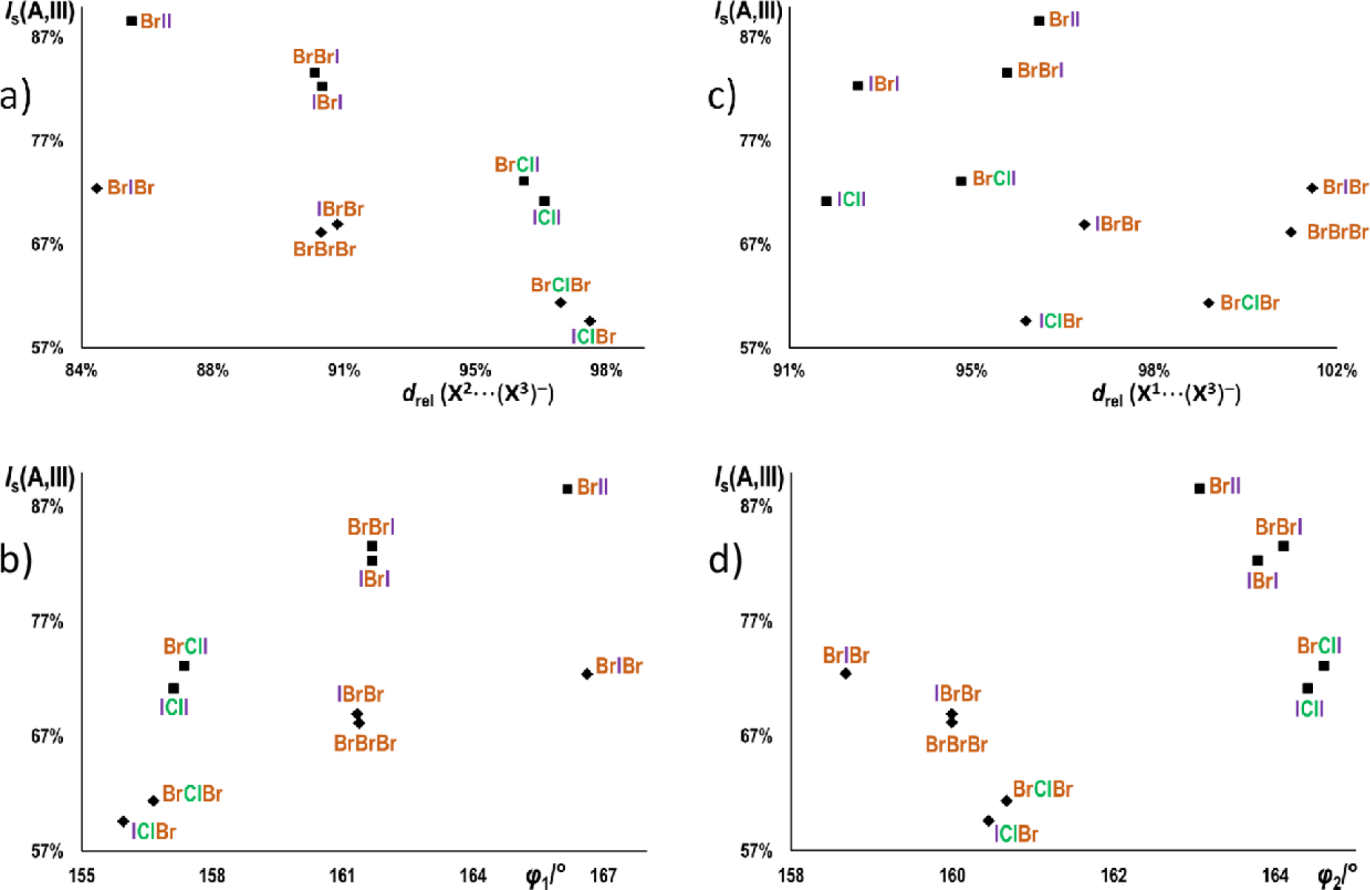
Correlations between the halogen bond parameters and the isostructurality index with respect to **III** (*I*_s_(A,**III**), A ≠ **III**)) for structures of the type I series with (a) relative length of the **X**^**2**^···**X**^**3**^ halogen bond, *d*_rel_(**X**^**2**^···**X**^**3**^) plot; (b) angle of the **X**^**2**^···**X**^**3**^ halogen bond, φ_1_ plot; (c) relative length of the **X**^**1**^···**X**^**3**^ halogen bond, *d*_rel_(**X**^**1**^···**X**^**3**^), (d) angle of the **X**^**1**^···**X**^**3**^ halogen bond, φ_2_. Structures of iodide salts are represented by squares and bromide salts as rhombi.

The correlations between the halogen bond parameters and the isostructurality index with respect to **III** (*I*_s_(A,**III**) for structures of the type I series (A ≠ **III**)) are given in Figure 5. It was noted earlier that the structures are more affected by the exchange of **X**^**3**^ than either **X**^**1**^ or **X**^**2**^. This is once more demonstrated by the plots of *I*_s_(A,**III**) vs the halogen bond lengths and angles, which show that the dominant determinator of the similarity of the structures is not the halogen bond geometry, but rather the anion, with the iodides more similar to **III** than the bromides. However, there is a definite correlation between the *I*_s_(A,**III**) and both relative length and the angle of the (stronger) **X**^**2**^···(**X**^**3**^)^−^ halogen bond: within both the iodide and the bromide series, there is an increase in *I*_s_(A,**III**) as the halogen bond becomes more linear and a decrease as it becomes longer. As the **X**^**2**^···(**X**^**3**^)^−^ halogen bond in **III** is (relatively) shorterst and most linear within the type I structures (*d*_rel_(**X**^**2**^···**X**^**3**^) = 85.49%; φ_1_ = 166.25°), it follows that an increase in similarity of the halogen bond parameters should lead to an increase in the overall similarity of the crystal structures. Conversely, there is no discernible correlation between the *I*_s_(A,**III**) and the relative length of the (weaker) **X**^**1**^···(**X**^**3**^)^−^ halogen bond—the plot of *I*_s_(A,**III**) versus *d*_rel_(**X**^**1**^···**X**^**3**^) reveals only the general trends of *I*_s_(A,**III**) rising with the sizes of all three halogens. However, the angle of the **X**^**2**^···(**X**^**3**^)^−^ halogen bond does show a definite correlation with the *I*_s_(A,**III**)—again the same general trend among the iodides and the bromides—of reduction of the similarity of structures as compared to **III** with the increase of φ_2_. As **III** has one of the smallest φ_2_ angles in the series (φ_2_(**III**) = 163.52°), the decrease of similarity to **III** with the increase of φ_2_ is to be expected. On the other hand, as all the bromides have lower φ_2_ angles than **III**, the increasing similarity in values of φ_2_ is *reducing* the overall similarity of the crystal structures. However, the increase of φ_2_ follows the decrease in the **X**^**2**^···(**X**^**3**^)^−^ halogen bond strength (notice that in both iodides and bromides it increases with the reduction of **X**^**2**^: **X**^**1**^**IX**^**3**^ < **X**^**1**^**BrX**^**3**^ < **X**^**1**^**ClX**^**3**^). It can therefore be concluded that the structural similarity within the type I is primarily determined by the anion and the **X**^**2**^···(**X**^**3**^)^−^ halogen bond, the observed correlation between *I*_s_(A,**III**) and φ_2_ being one of the consequences thereof.

#### Type II Structures

**Figure 6:**
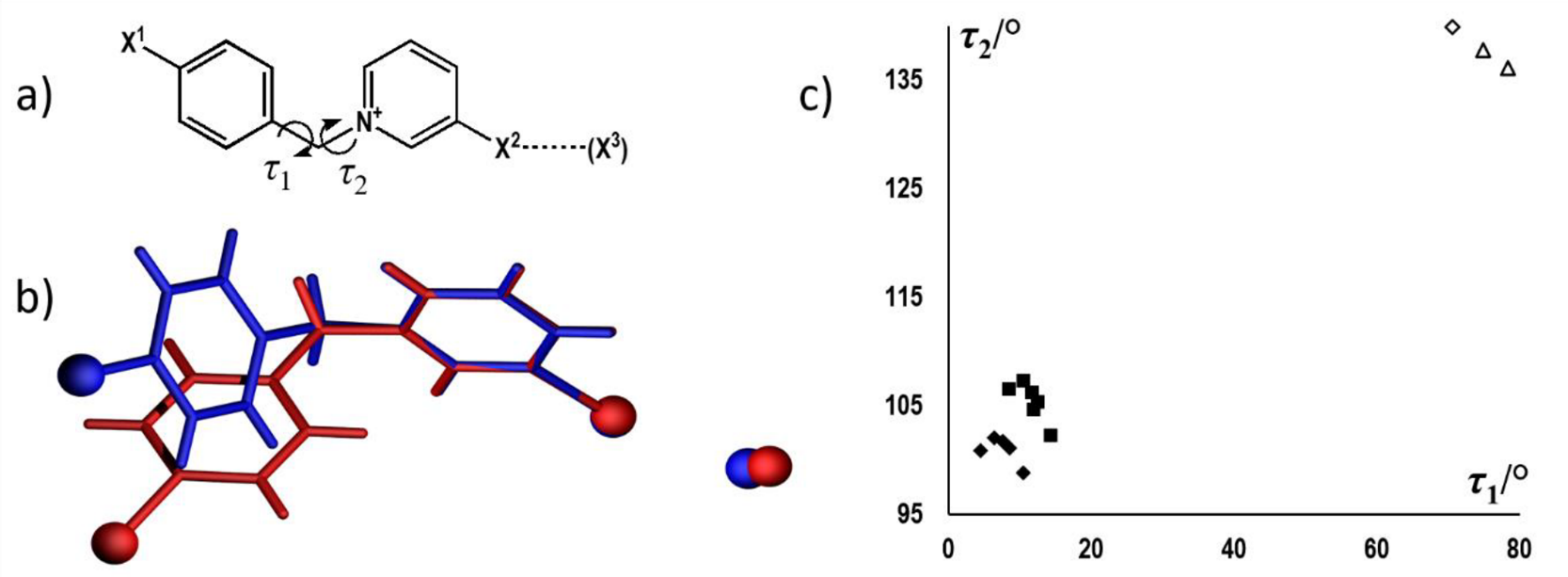
Comparison of conformations of cations in type I and type II structures: (a) τ_1_ and τ_2_ torsion angles, (b) overlay of a cation–anion pair (pyridine ring defined as the anchor) in a type I structure (**BrIBr**, red) and a type II structure (**IIBr**, blue), (c) scatterplot of τ_1_ and τ_2_ torsion angles for type I (black) and type II structures (white with black border). Structures of iodide salts are represented by squares, bromide salts as rhombi, and chloride as triangles.

The two chloride salts which did not crystallize as hydrates (**IICl** and **BrICl**), as well as one of the bromides (**IIBr**), form a second isostructural series (type II). Type II structures are somewhat less closely packed than the type I structures (average KPC 68.9 for type I and 66.9 for type II). They also comprise halogen-bonded chains; however, there are significant differences as compared with Type I structures. One of the differences is the conformation of the cation (Figure 6a,b), which is best described by the two torsion angles about the two single bonds: τ_1_ (C12–C7–C6–N1) and τ_2_ (C1–N1–C6–C7): while in type I structures τ_1_ is generally quite small (in the range 5–12°), in type II structures it increases to above 70°. The values of τ_2_ also increase from 98° to 108° in type I structures (where τ_2_ is somewhat dependent on the counterion, being under 102.1° in all bromides and above 102.2° in all iodides) to 136–140° (Figure 6c). This increase in the torsion angles leads to an increase of the overall length of the hydrocarbon skeleton of the cation (*d*_cat_, measured as the distance between the two halogenated carbon atoms (C2 and C10) on the same cation, see Scheme 2) from ca. 9.5 Å to 10.1 Å in type I to ca. 10.3 to 10.6 Å in type II.

**Figure 7:**
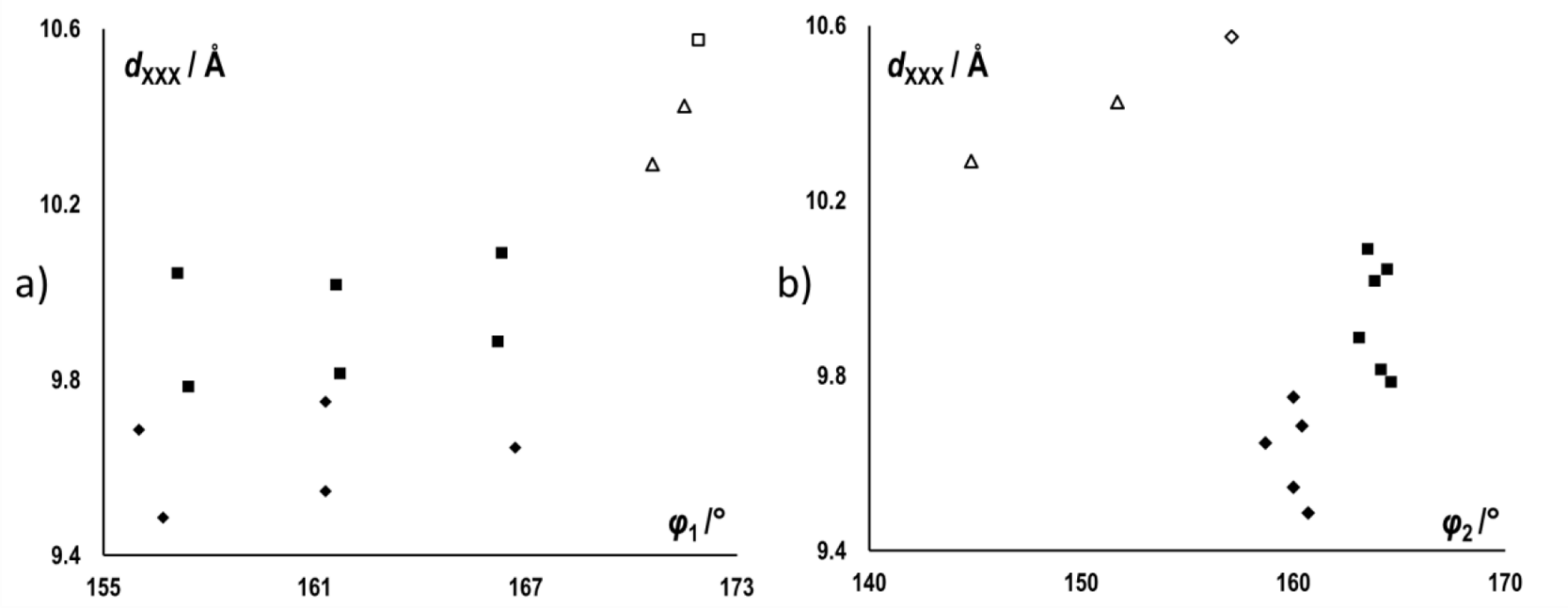
Parameters describing the halogen-bonded chains in type I (black) and type II structures (white with black border): length of the fragment of the halogen-bonded chain which contains both halogen bonds (*d*_XXX_) vs (a) angle of the **X**^**2**^···(**X**^**3**^)^−^ halogen bond (φ_1_) and (b) *s* angle of the **X**^**1**^···(**X**^**3**^)^−^ halogen bond (φ_2_). Structures of iodide salts are represented by squares, bromide salts as rhombi, and chloride as triangles.

The most obvious difference in the halogen-bonded chains in type II structures, as compared to type I, is in the angle between the two halogen bonding contacts formed by the anion (ϑ, see Figure S36 in the Supporting Information), which in type I always remains within the range of ca. 123.8–126.4°, while in type II it takes up values between ca. 150.8 and 153.2° (Figure 7). As ϑ increases, so does the length of the fragment of the halogen-bonded chain which contains both halogen bonds (*d*_XXX_, measured as the distance between the two halogenated carbon atoms (C2 and C10′) on neighboring cations forming halogen bonds with the same anion see Figure S36 in the Supporting Information). Interestingly, although both *d*_cat_ and *d*_XXX_ are considerably longer in type II structures, the overall length of a unit of the halogen-bonded chain (*d*_chain_) differs relatively little between the two types (Figure S36 in the Supporting Information)—bromides of type I and II differ by ca. 0.8 Å in *d*_XXX_ and ca. 0.4 in *d*_cat_, while the difference in *d*_chain_ is only ca. 0.5 Å (much less than *d*_XXX_ + *d*_cat_). This is because the increase in *d*_XXX_ and *d*_cat_ in type II structures is compensated for by changes in halogen bond geometries. The halogen bond involving the pyridine halogen donor (**X**^**2**^···(**X**^**3**^)^−^) in type II structures is both shorter and more linear (Figure 3a) than in type I structures. On the other hand, the halogen bond involving the benzyl halogen donor (**X**^**1**^···(**X**^**3**^)^−^) is generally shorter, but also less linear in type II structures, leading to an overall decrease in the C10···(**X**^**3**^)^−^ distance (Figure 3b).

**Figure 8:**
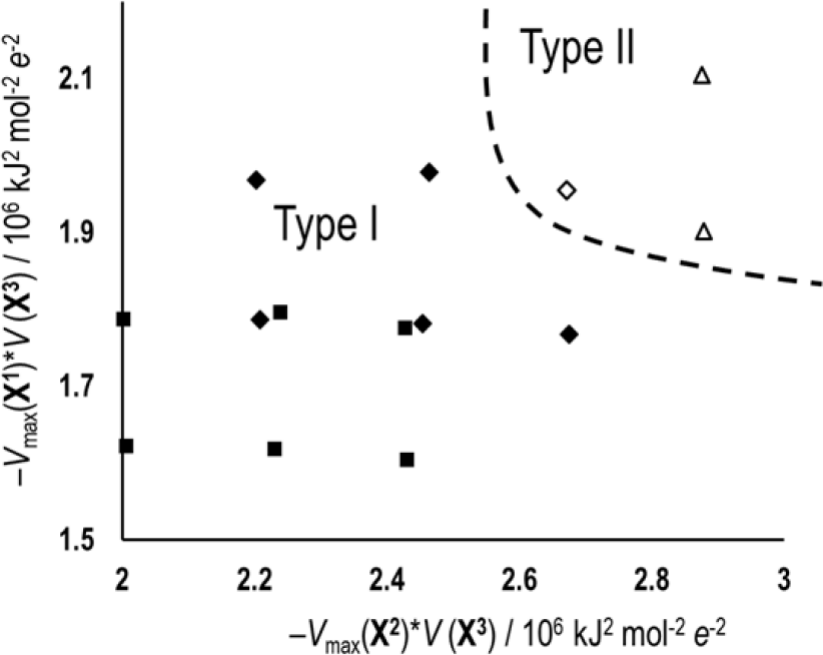
Distribution of the two isostructural series on a plot of the products of the electrostatic potentials of weaker cation donor and halogenide anion acceptor (*V*_max_(**X**^**1**^)*V (**X**^**3**^)) vs products of the electrostatic potentials of stronger cation donor and halogenide anion acceptor (*V*_max_(**X**^**2**^)*V (**X**^**3**^)).

The differences between the halogen bonds in the two structural types indicate the probable cause for the existence of the two types. The main discriminator between the two structural types is the halogen bond, primarily, the dominant **X**^**2**^···(**X**^**3**^)^−^ halogen bond. The increased ϑ in type II structures allows for a closer approach of the donor (**X**^**2**^) to the acceptor ((**X**^**3**^)^−^) and therefore a more favorable geometry leading to a higher bond energy. Importantly, type II is achieved by the combination of the strongest halogen bond donors (iodopyridinium derivatives) and the strongest acceptors (chloride anions). The bromide anion also forms a type II structure with one iodopyridinium donor (**IIBr**) but not the other (**BrIBr** crystallizes as type I), indicating that the benzyl halogen (and therefore the **X**^**1**^···(**X**^**3**^)^−^ halogen bond) is also a factor in discriminating between the two types of structure. This principle is illustrated in Figure 8 where the distribution of the two types is shown on a plot of the products of the electrostatic potentials of cation donors and halogenide anion acceptors (being a convenient measure for the relative potential for halogen bonding in a given donor/acceptor pair within a closely related group).

Another convenient descriptor of the halogen bond strength is the *in vacuo* interaction energy of the ion pairs with the **X**^**2**^···(**X**^**3**^)^−^ (or **X**^**1**^···(**X**^**3**^)^−^) contacts with geometries as found in the crystals (*E*(**X**^**2**^···**X**^**3**^) viz. *E*(**X**^**1**^···**X**^**3**^)). While these energies are by no means realistic estimates of the energies of the corresponding contacts in the solid state, they are indicative of the general trends these energies should follow. When these are calculated for the **X**^**2**^···(**X**^**3**^)^−^ contacts for all 14 structures, they show that **X**^**2**^···(**X**^**3**^)^−^ contacts in geometries corresponding to type II correspond to higher interaction energies than those of type I geometries (Figure S37 in the Supporting Information). This once more suggests that the energy gain due to the more favorable geometry of the **X**^**2**^···(**X**^**3**^)^−^ halogen bond is the main driving force for the formation of type II structures.

### Thermal Properties of Type I Structures

The size of the type I isostructural series has prompted us to investigate whether there is a clear and measurable effect of the halogen bond on the thermal properties of the type I solids. As within the isostructural series the only significant difference is in the halogen bond donors and acceptors, it can be expected that the differences in the halogen bond energies in various crystals will be the dominant cause of differences in their thermal properties. In order to investigate this, we have performed thermal analysis (TG and DSC) for the compounds which have crystallized with type I structures. These have shown that nine of these salts undergo evaporation in the temperature range from 170 to 200 °C without previous thermal events. The evaporation is characterized by a continuous loss of the entire mass of the sample in the TG, with the DSC curves generally exhibiting two endothermic signals (probably corresponding to almost simultaneous melting and evaporation) which are to a larger or lesser degree coalesced into one (Figures S17–S31 in the Supporting Information). In the case of two compounds (**III** and **BrIBr**), the DSC curve is more complex, with additional signals at lower temperatures and also somewhat lower temperatures of the melting/evaporation event (163 and 169 °C, respectively). As their thermal behavior is obviously different from that of the remaining members of the series, they have been excluded from the further analysis of the data.

**Figure 9:**
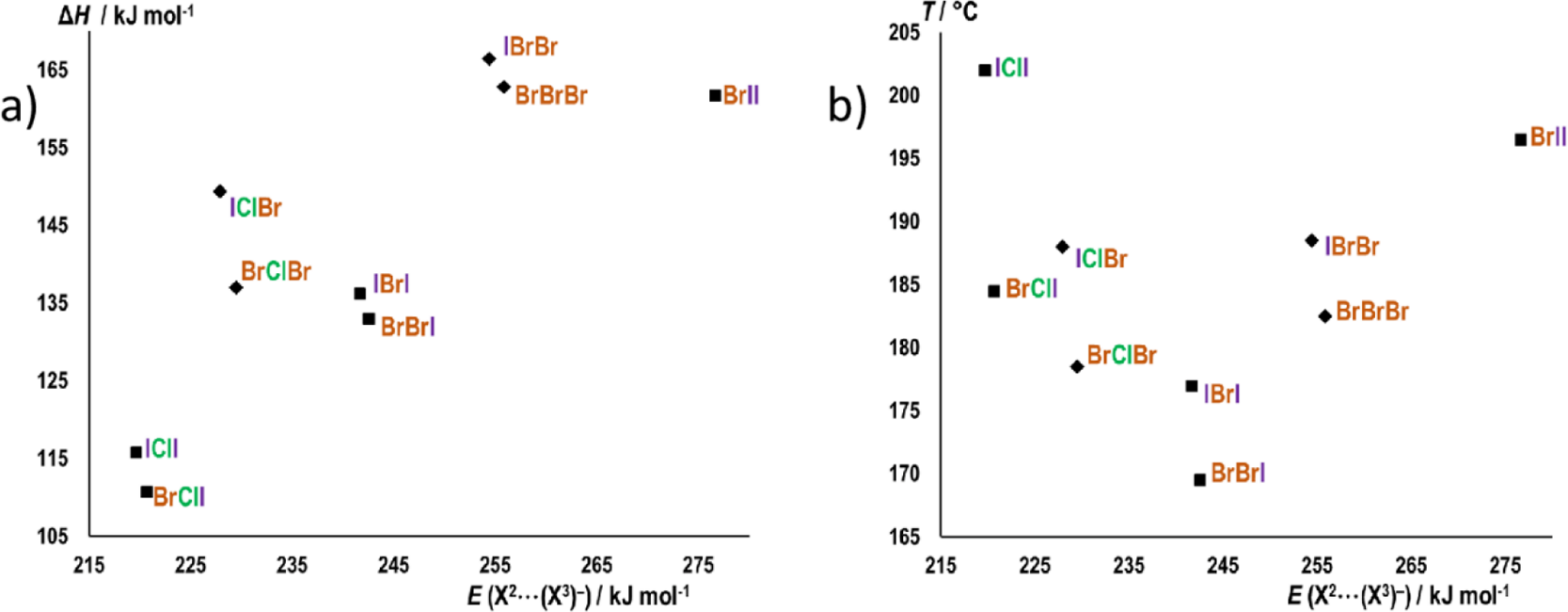
Correlation between the computed **X**^**2**^···(**X**^**3**^)^−^ interaction energies of ion pairs (computed *in vacuo* for geometries as found in the crystals, *E*(**X**^**2**^···(**X**^**3**^)^−^)) and (a) the evaporation enthalpies (**Δ*****H***), (b) onset temperatures (***T***) of the melting/evaporation within the type I structures. Structures of iodide salts are represented by squares and bromide salts as rhombi.

There is a clear linear correlation between the *E*(**X**^**2**^···**X**^**3**^) and the evaporation enthalpies within the type I structures (Figure 9a), which is particularly evident when comparing structures which differ only in **X**^**2**^ (for the **BrII**–**BrBrI**–**BrClI** series, *R*^2^ = 0.997). The differences in evaporation enthalpies between compounds which differ only in **X**^**2**^ are generally similar to the corresponding differences in *E*(**X**^**2**^···**X**^**3**^) (Table S4 in the Supporting Information). As for the weaker, **X**^**1**^···(**X**^**3**^)^−^ halogen bond, there is no apparent correlation between the enthalpies and the gas phase interaction energy *E*(**X**^**1**^···**X**^**3**^) (Figure S32 in the Supporting Information). However, the nature of **X**^**1**^ also slightly affects the evaporation enthalpy, as within each **BrX**^**2**^**X**^**3**^/**IX**^**2**^**X**^**3**^ pair of structures, the **IX**^**2**^**X**^**3**^ analogue has higher evaporation enthalpy (by 3.5–12.5 kJ mol^–1^), indicating the contribution of the **X**^**1**^···(**X**^**3**^)^−^ bond to the total packing energy.

The evaporation enthalpy is also significantly affected by the nature of the halogenide (i.e., halogen bond acceptor)—while following the same trend with respect to *E*(**X**^**2**^···**X**^**3**^), the bromides systematically have higher evaporation enthalpies than the iodides. This cannot be accounted for by a difference in halogen bonding. Rather, the most likely cause for this difference between the iodides and the bromides lies in the difference between the C–H···X^–^ hydrogen bond energies for iodide and bromide. In all structures, the anion, along with the halogen bonds within a halogen-bonded chain, also forms C–H···X^–^ hydrogen bonds with cations from neighboring chains. As bromide is a stronger hydrogen bond acceptor than the iodide, more energy is required in order to sever the C–H···Br^–^ hydrogen bonds upon evaporation, leading to higher overall enthalpies.

Unlike the evaporation enthalpies which are clearly dominated by the contribution of the **X**^**2**^···(**X**^**3**^)^−^ halogen bonds, the onset temperatures of the melting/evaporation show a somewhat more complex behavior (Figure 9b). For stronger **X**^**2**^···(**X**^**3**^)^−^ halogen bonds (with **X**^**2**^ = Br, I), the onset temperatures expectedly increase with the *E*(**X**^**2**^···**X**^**3**^), again with iodobenzyl derivatives at somewhat higher onset temperatures than their bromobenzyl analogues (by 6–7.5 °C). However, in the case of weaker **X**^**2**^···(**X**^**3**^)^−^ halogen bonds (with **X**^**1**^ = Cl), the trend is apparently the opposite—onset temperatures decrease with *E*(**X**^**2**^···**X**^**3**^). The differences in the onset temperatures within the **BrX**^**2**^**X**^**3**^/**IX**^**2**^**X**^**3**^ pairs are also higher (by 7.5–17.5 °C). The latter observation is in line with the larger contribution of the **X**^**1**^···(**X**^**3**^)^−^ halogen bond to the overall packing energy (due to the reduced contribution of **X**^**2**^···(**X**^**3**^)^−^ halogen bond because of relatively lower *V*_max_(Cl)—see discussion above). The increase of the onset temperatures with the decrease of *E*(**X**^**2**^···**X**^**3**^) among the chloropyridine derivatives is however somewhat more difficult to account for. As the evaporation enthalpies change regularly with *E*(**X**^**2**^···**X**^**3**^), the most probable reason for the different trend in melting/evaporation onset temperatures is a different trend in the lattice entropies. It can be expected that within the **X**^**1**^**ClX**^**3**^ series the lattice entropy is higher (due to less constricted thermal motion in less strongly bonded structures), increasing with decreasing *E*(**X**^**2**^···**X**^**3**^). As the lattice entropy increases, the entropy change upon melting/evaporation decreases, which can be expected to cause an increase in the phase transition temperature. However, as only four data points are involved, one cannot exclude the possibility that the apparent trend is merely an artifact of a random distribution.

## Conclusion

*N*-(4-Halogenobenzyl)-3-halogenopyridinium halogenides have proven to be an excellent platform for the study of isostructural halogen exchange, as they fall within two series of isostructural solids. The larger series (type I) comprises 11 structures and allows for an in-depth study of the effect of halogen exchange (and therefore differences in the halogen bonding) on the crystal structure and properties. The halogenide anion acts as an acceptor of two inequivalent halogen bonds with neighboring cations. Of these, the one formed by the halogen on the pyridine ring (which exhibits a more positive σ-hole potential than the halogen on the benzyl ring, even in the case of chloropyridine derivatives) is dominant—it adopts the optimal geometry (within the given structure type), while the other halogen bond distorts in order to accommodate it. This halogen bond is also the reason for the appearance of the second structural type, which allows it to adopt an even more favorable geometry. Also, it is the main cause of the differences in evaporation enthalpies within the type I structures. However, the halogen bond involving the benzyl ring halogen as the donor also has a noticeable effect, both on the structure (within the **IIBr**/**BrIBr** pair the nature of this donor is the discriminating factor of the structural type) and on the properties (within each **BrX**^**2**^**X**^**3**^/**IX**^**2**^**X**^**3**^ pair of structures, the **IX**^**2**^**X**^**3**^ analogue has higher evaporation enthalpy). It can therefore be concluded that although one halogen bond is clearly dominant, the interplay of both is responsible for the structural features of *N*-(4-halogenobenzyl)-3-halogenopyridinium halogenides. Indeed, it can be hypothesized that the fact that such a large number of cation/anion combinations (11) adopt essentially the same structure (type I) is due, on the one hand, to the existence of strongly directing interactions which ensure the same supramolecular topology (chains), and on the other to a sufficient degree of flexibility in the weaker halogen bond (and somewhat in the cation structure), which can adapt in order to compensate for the changes in the atom size and (stronger) halogen bond length and angle.

## Experimental Section

### Synthesis

All solvents (acetone, dichloromethane, ethanol), acids (hydrochloric and hydroiodic), and the ion-exchange resin (Dowex 21K chloride form, 16-30 mesh) were purchased from Sigma-Aldrich Company. 3-Chloropyridine, 3-bromopyridine, and (4-bromobenzyl) bromide were purchased from Acros Organics, while 3-iodopyridine (4-iodobenzyl) bromide was from Apollo Scientific. All the solvents and reagents were used as received.

*N*-(4-Halogenobenzyl)-3-halogenopyridinium bromides were prepared by dissolving equimolar amounts (1 mmol) of corresponding 3-halogenopyridine and (4-halogenobenzyl) bromide in hot acetone (20 mL) whereupon the solutions were left to cool and evaporate.

Iodide and chloride salts of *N*-(4-halogenobenzyl)-3-halogenopyridinium cations were synthesized by ion exchange. *N*-(4-Halogenobenzyl)-3-halogenopyridinium hydroxides were prepared by passing the solutions of *N*-(4-halogenobenzyl)-3-halogenopyridinium bromides in deionized water (*c* = 0.1 mol L^–1^) through the anion exchange column. The ion-exchange resin was regenerated with a 50 mL aqueous solution of sodium hydroxide (*c* = 1 mol L^–1^). Obtained solutions of *N*-(4-halogenobenzyl)-3-halogenopyridinium hydroxides were neutralized with hydroiodic acid (*c* = 1 mol L^–1^). Hydroiodic acid was added dropwise in the obtained solution until neutralization. The same procedure was used to prepare the chloride salts using hydrochloric acid instead of hydroiodic acid.

Single crystals (suitable for single crystal X-ray diffraction experiment) of *N*-(4-halogenobenzyl)-3-halogenopyridinium bromides and iodides were obtained directly from synthesis. In the case of chlorides, single crystals were obtained by recrystallizing the obtained solid product from a mixture of dry ethanol and dry acetone.

### X-ray Diffraction Measurements

All single-crystal X-ray diffraction experiments were performed using an Oxford Diffraction XtaLAB Synergy, Dualflex, HyPix X-ray four-circle diffractometer with mirror-monochromated MoKα (λ = 0.71073 Å) radiation. The data sets were collected using the ω-scan mode over the 2θ range up to 60°. Programs CrysAlis PRO CCD and CrysAlis PRO RED were employed for data collection, cell refinement, and data reduction.^51,52^ The structures were solved by SHELXT^53^ or by direct methods using the SHELXS and refined using SHELXL programs.^54^ The structural refinement was performed on *F*^2^ using all data. The hydrogen atoms were placed in calculated positions and treated as riding on their parent atoms (C–H = 0.95 Å and *U*_iso_(H) = 1.2 *U*_eq_(C) for aromatic hydrogen atoms; C–H = 0.99 Å and *U*_iso_(H) = 1.2 *U*_eq_(C) for methylene hydrogen atoms). In the case of both hydrates, the water hydrogen bonding atoms were located in the electron difference map. All calculations were performed using the WinGX^55^ or Olex2 1.3-ac4^56^ crystallographic suite of programs. A summary of data pertinent to X-ray crystallographic experiments is provided in Table S1 (see Supporting Information). Further details are available from the Cambridge Crystallographic Centre (CCDC 2118712–2118727 contain crystallographic data for this paper). Molecular structures of compounds and their packing diagrams were prepared using Mercury.^57^

### Thermal Analysis

Differential scanning calorimetry (DSC) and thermogravimetric (TG) measurements were performed simultaneously on a Mettler Toledo TGA/DSC 3+ module. Samples were placed in alumina crucibles (40 μL) and heated 25 to 300 °C, at a heating rate of 10 °C min^–1^ under nitrogen flow of 50 mL min^–1^.

Data collection and analysis were performed using the program package STARe Software (Version 15.00, Mettler Toledo, Greifensee, Switzerland).^58^ TG and DSC thermograms of the prepared compounds are shown in Figures S11–S15 in Supporting Information.

### Computational Details

All calculations were performed using the Gaussian 09 software package.^59^ Geometry optimizations of cation molecules for analysis of the molecular electrostatic potential were performed using the b3lyp/dgdzvp level of theory.^60,61^ Harmonic frequency calculations were performed on the optimized geometries to ensure the success of each geometry optimization. Single-point energies of the ion pairs *in vacuo* were determined using the M062X/dgdzvp^62^ lever of theory on geometries obtained by X-ray crystallography. This combination of the functional and the basis set was shown to reproduce experimental halogen bond energies in the gas phase with good accuracy, which are comparable to energies obtained by using larger and more time-consuming triple-ζ basis sets.^63^ Interaction energies between ions were calculated and corrected by basis set superposition errors (BSSE) according to the counterpoise method of Boys and Bernardi.^64,65^ The figures were prepared using GaussView.^66^

## Supporting Information Available

Crystallographic data for all compounds; ORTEP representations of the formula units of the prepared compounds; TG and DSC curves; an overview of supramolecular interactions in studied *N*-(4-halogenobenzyl)-3-halogenopyridinium halogenides; calculated ESP products and halogen bond energies; calculated Kitaigorodsky packing coefficient (KPC) and other parameters describing the halogen-bonded chains; measured melting/sublimation point temperatures and enthalpies, correlation between the computed X^1^···(X^3^)–halogen bond energies and (a) the evaporation enthalpies (Δ*H*), (b) onset temperatures (*T*) of the melting/evaporation within the type I structures; correlation of *I*s and the unit cell parameters of type I structures: (a) *I*s vs *V* plot, (b) *I*s vs *a* plot, (c) *I*s vs *b* plot, (d) *I*s vs *c* plot, (e) *I*s vs β plot, (f) *I*s vs *M*_r_ plot; plot of the distance between the (a) [101] planes and (b) [1̅ 0 1̅] planes vs isostructurality indices (*I*s(A, III)) for the 11 crystals belonging to the structural type I; total electron energies and Cartesian coordinates for optimized structures of *N*-(4-halogenobenzyl)-3-halogenopyridinium cations; single-point electron energies and Cartesian coordinates of *N*-(4-halogenobenzyl)-3-halogenopyridinium halogenides (PDF)

## Accession Codes

CCDC 2118712–2118727 contain the supplementary crystallographic data for this paper. These data can be obtained free of charge via www.ccdc.cam.ac.uk/data_request/cif, or by emailing data_request@ccdc.cam.ac.uk, or by contacting The Cambridge Crystallographic Data Centre, 12 Union Road, Cambridge CB2 1EZ, UK; fax: +44 1223 336033.

## Dedication

Dedicated to Dora Bedeković, in honor of her birth.
