# Supplementary material for: Isostructural Halogen Exchange and Halogen Bonds: The Case of N-(4-Halogenobenzyl)-3-halogenopyridinium Halogenides
Source: Cryst Growth Des. 2022 Jan 5;22(2):1333–44. doi: 10.1021/acs.cgd.1c01285 (PMC8889897; doi:10.1021/acs.cgd.1c01285)
Supplement: Supplementary file 1 — cg1c01285_si_001.pdf [file cg1c01285_si_001.pdf]

**SUPPORTING INFORMATION**

# Isostructural halogen exchange and halogen bond – the case of *N*-(4-halogenobenzyl)-3-halogenopyridinium halogenides

Luka Fotović, Nikola Bedeković and Vladimir Stilinović

*Department of Chemistry, Faculty of Science, University of Zagreb, Horvatovac 102a, HR-10000 Zagreb, Croatia*

Email: vstilinovic@chem.pmf.hr
Fax: +385 1 4606 341
Tel: +385 1 4606 371

## Table of Contents

**Table of Content**


| Item | Page |
|---|---|
| **Table S1** (Crystallographic data) | 3 |
| **Figures S1–S16** (ORTEP representations of the formula units of the prepared compounds) | 7 |
| **Figures S17–S31.** (DSC and TG curves of the prepared compounds) | 15 |
| **Table S2.** Halogen bond lengths, the corresponding relative bond lenght ($d_{rel}$) and angles in studyed *N*-(4-halogenobenzyl)-3-halogenopyridinium halogenides. | 20 |
| **Table S3.** C–H···$(X^3)^-$ hydrogen bond lengths and angles in studyed *N*-(4-halogenobenzyl)-3-halogenopyridinium halogenides. | 21 |
| **Table S4.** Calculated electrostatic potentials products and halogen bond energies of studyed *N*-(4-halogenobenzyl)-3-halogenopyridinium halogenides. | 23 |
| **Table S5.** Calculated Kitaigorodsky packing coefficient (KPC) and other parameters describing the halogen bonded chains (defined on the Figure 7 in the main text) used for comparison of type I and type II structures of studyed *N*-(4-halogenobenzyl)-3-halogenopyridinium halogenides. | 23 |
| **Table S6.** Mesured melting/sublimation point temepratures and enthapyes of studyed *N*-(4-halogenobenzyl)-3-halogenopyridinium halogenides. | 24 |
| **Figure S32.** Correlation between the computed $\mathbf{X^1}$···$(\mathbf{X^3})^-$ halogen bond energies and: a) the evaporation enthalpies (**Δ*H***), b) onset temperatures (**T**) of the melting/evaporation within the type I structures. | 24 |

| | |
|---|---|
| **Figure S33.** Correlation of $I_s$ and the unit cell parameters of type I structures; a) $I_s$ *vs.* $V$ plot, b) $I_s$ *vs.* $a$ plot, c) $I_s$ *vs.* $b$ plot, d) $I_s$ *vs.* $c$ plot, e) $I_s$ *vs.* $\beta$ plot, f) $I_s$ *vs.* $M_r$ plot. Structures of iodide salts are represented by squares, bromide salts as rhombi and chloride as triangles. | 25 |
| **Figure S34.** Plot of the distance between the a) [101] planes and b) [-10-1] planes *vs* isostructurality indices ($I_s$(A,III)) for the 11 crystals belonging to the structural type I. | 25 |
| **Figure S35.** Halogen bonded chains within the unit cell in type I structures (in BrII; a), b) and c)) and type II structures (in IICl; d), e) and f)). Both structures viewed along: a), d) the crystallographic *c* axis; b), e) the crystallographic *a* axis; c), f) the crystallographic *b* axis. | 26 |
| **Figure S36.** Parameters describing the halogen bonded chains in type I (black) and type II structures (white with black border): a) total length of a unit of the halogen bonded chain ($d_{chain}$) *vs* angle between the two halogen bonds ($\theta$) and b) length of the fragment of the halogen bonded chain which contains both halogen bonds ($d_{XXX}$) *vs* the length of the hydrocarbon skeleton of the cation ($d_{cat}$). Structures of iodide salts are represented by squares, bromide salts as rhombi and chloride as triangles. | 26 |
| **Figure S37.** Distribution of the two isostructural series on a plot of the energies of ion pairs (computed *in vacuo* for geometries as found in the crystals, $E(\mathbf{X}^2\cdots(\mathbf{X}^3)^-)$) *vs* the Kitaigorodsky packing coefficient (KPC). | 27 |
| Total electron energies and Cartesian coordinates for optimised structures of *N*-(4-halogeno)benzyl-(3-halogeno)pyridinium cations | 28 |
| Single point electron energies and Cartesian coordinates of *N*-(4-halogeno)benzyl-(3-halogeno)pyridinium halogenides | 34 |

**Table S1.** Crystal data and refinement details for the prepared salts.

| | **(*N*-4Ito-3Ipy)**I | **(*N*-4Ito-3Brpy)**I | **(*N*-4Ito-3Clpy)**I | **(*N*-4Brto-3Ipy)**I |
|---|---|---|---|---|
| Molecular formula | $C_{12}H_{10}NI_3$ | $C_{12}H_{10}NI_2Br$ | $C_{12}H_{10}NI_2Cl$ | $C_{12}H_{10}NI_2Br$ |
| $M_r$ | 548.91 | 501.93 | 457.48 | 501.93 |
| Crystal system | monoclinic | monoclinic | monoclinic | monoclinic |
| Space group | $P\,2_1/c$ | $P\,2_1/c$ | $P\,2_1/c$ | $P\,2_1/c$ |
| Crystal data: | | | | |
| $a$ / Å | 12.2504(7) | 12.2416(5) | 12.1910(2) | 12.0195(4) |
| $b$ / Å | 8.8065(4) | 8.5540(3) | 8.4755(2) | 8.7462(3) |
| $c$ / Å | 13.6992(7) | 13.6601(5) | 13.6350(3) | 13.6013(4) |
| $\alpha$ / ° | 90 | 90 | 90 | 90 |
| $\beta$ / ° | 100.560(5) | 100.298(4) | 99.747(2) | 100.933(3) |
| $\gamma$ / ° | 90 | 90 | 90 | 90 |
| $V$ / Å$^3$ | 1452.88(13) | 1407.37 | 1388.5 | 1403.88 |
| $Z$ | 4 | 4 | 4 | 4 |
| $D_{calc}$ / g cm$^{-3}$ | 2.51 | 2.37 | 2.19 | 2.37 |
| $\lambda$(Mo$K_\alpha$) / Å | 0.71073 | 0.71073 | 0.71073 | 0.71073 |
| $T$ / K | 170 | 170 | 170 | 170 |
| Crystal size / mm$^3$ | 0.10 x 0.16 x 0.18 | 0.12 x 0.16 x 0.18 | 0.16 x 0.14 x 0.05 | 0.18 x 0.14 x 0.12 |
| $\mu$ / mm$^{-1}$ | 6.430 | 7.283 | 4.969 | 7.301 |
| $F$(000) | 992 | 920 | 848 | 920 |
| Refl. collected/unique | 17640 / 4896 | 14899 / 3743 | 24533 / 3024 | 13069 / 3056 |
| Data/restraints/ parameters | 145 | 145 | 145 | 145 |
| $\Delta\rho_{max}$ , $\Delta\rho_{min}$ / e Å$^{-3}$ | 0.899; –1.070 | 1.972; –1.420 | 0.551; –0.761 | 0.746; –0.807 |
| $R[F^2 > 4\sigma(F^2)]$ | 0.033 | 0.041 | 0.020 | 0.025 |
| w$R(F^2)$ | 0.064 | 0.104 | 0.045 | 0.058 |
| Goodness-of-fit, $S$ | 1.021 | 1.029 | 1.071 | 1.025 |

**Table S1.** Continued.

| | **(*N*-4Brto-3Brpy)**I | **(*N*-4Brto-3Clpy)**I | **(*N*-4Ito-3Ipy)**Br | **(*N*-4Ito-3Brpy)**Br |
|---|---|---|---|---|
| Molecular formula | $C_{12}H_{10}NBr_2I$ | $C_{12}H_{10}NI_2Cl$ | $C_{12}H_{10}NI_2Br$ | $C_{12}H_{10}NBr_2I$ |
| $M_r$ | 454.93 | 410.47 | 501.93 | 454.93 |
| Crystal system | monoclinic | monoclinic | monoclinic | monoclinic |
| Space group | $P\,2_1/c$ | $P\,2_1/c$ | $P\,2_1/c$ | $P\,2_1/c$ |
| Crystal data: | | | | |
| $a$ / Å | 11.9930(5) | 11.9546(4) | 6.2000(2) | 12.1206(4) |
| $b$ / Å | 8.4851(3) | 8.3900(2) | 7.7212(2) | 8.4319(3) |
| $c$ / Å | 13.5746(5) | 13.5493(4) | 30.2458(10) | 13.2541(5) |
| $\alpha$ / ° | 90 | 90 | 90 | 90 |
| $\beta$ / ° | 100.486(4) | 99.933(3) | 95.026(3) | 100.610(3) |
| $\gamma$ / ° | 90 | 90 | 90 | 90 |
| $V$ / Å$^3$ | 1359.11 | 1338.61 | 1442.34 | 1331.41 |
| $Z$ | 4 | 4 | 4 | 4 |
| $D_{calc}$ / g cm$^{-3}$ | 2.22 | 2.04 | 2.31 | 2.27 |
| $\lambda$(Mo$K_\alpha$) / Å | 0.71073 | 0.71073 | 0.71073 | 0.71073 |
| $T$ / K | 170 | 170 | 170 | 170 |
| Crystal size / mm$^3$ | 0.18 x 0.05 x 0.03 | 0.21 x 0.16 x 0.11 | 0.25 x 0.15 x 0.04 | 0.18 x 0.15 x 0.14 |
| $\mu$ / mm$^{-1}$ | 8.214 | 5.550 | 7.106 | 8.380 |
| $F$(000) | 848 | 776 | 920 | 848 |
| Refl. collected/unique | 13731 / 2965 | 11210 / 2927 | 12841 / 2535 | 14904 / 3877 |
| Data/restraints/ parameters | 145 | 145 | 146 | 145 |
| $\Delta\rho_{max}$ , $\Delta\rho_{min}$ / e Å$^{-3}$ | 0.656; –0.650 | 0.864;-1.856 | 0.991; –0.935 | 0.885; –0.684 |
| $R[F^2 > 4\sigma(F^2)]$ | 0.026 | 0.038 | 0.038 | 0.028 |
| w$R(F^2)$ | 0.056 | 0.095 | 0.098 | 0.049 |
| Goodness-of-fit, $S$ | 1.065 | 1.056 | 1.062 | 1.050 |

**Table S1.** Continued.

| | **(*N*-4Ito-3Clpy)**Br | **(*N*-4Brto-3Ipy)**Br | **(*N*-4Brto-3Brpy)**Br | **(*N*-4Brto-3Clpy)**Br |
|---|---|---|---|---|
| Molecular formula | $C_{12}H_{10}NClBrI$ | $C_{12}H_{10}NBr_2Cl$ | $C_{12}H_{10}NBr_3$ | $C_{12}H_{10}NBr_2Cl$ |
| $M_r$ | 410.47 | 454.93 | 407.94 | 363.48 |
| Crystal system | monoclinic | monoclinic | monoclinic | monoclinic |
| Space group | $P\ 2_1/c$ | $P\ 2_1/c$ | $P\ 2_1/c$ | $P\ 2_1/c$ |
| Crystal data: | | | | |
| $a$ / Å | 12.0340(3) | 11.9724(4) | 11.8880(5) | 11.7965(4) |
| $b$ / Å | 8.3269(2) | 8.6064(3) | 8.3115(3) | 8.1983(3) |
| $c$ / Å | 13.2332(4) | 13.1674(5) | 13.1785(5) | 13.1751(5) |
| $\alpha$ / ° | 90 | 90 | 90 | 90 |
| $\beta$ / ° | 100.330(2) | 101.046(3) | 100.798(4) | 100.464(4) |
| $\gamma$ / ° | 90 | 90 | 90 | 90 |
| $V$ / Å$^3$ | 1304.55 | 1331.62 | 1279.07 | 1252.99 |
| $Z$ | 4 | 4 | 4 | 4 |
| $D_{calc}$ / g cm$^{-3}$ | 2.09 | 2.27 | 2.12 | 1.93 |
| $\lambda$(Mo$K_\alpha$) / Å | 0.71073 | 0.71073 | 0.71073 | 0.71073 |
| $T$ / K | 170 | 170 | 170 | 170 |
| Crystal size / mm$^3$ | 0.16 x 0.05 x 0.03 | 0.25 x 0.15 x 0.08 | 0.15 x 0.15 x 0.20 | 0.18 x 0.10 x 0.08 |
| $\mu$ / mm$^{-1}$ | 5.694 | 8.379 | 9.433 | 6.653 |
| $F$(000) | 776 | 848 | 776 | 704 |
| Refl. collected/unique | 28117 / 2846 | 14035 / 2893 | 15264 / 2784 | 14381 / 3970 |
| Data/restraints/ parameters | 145 | 145 | 145 | 145 |
| $\Delta\rho_{max}$ , $\Delta\rho_{min}$ / e Å$^{-3}$ | 1.038; –1.118 | 1.356; –0.977 | 0.504; –0.416 | 0.571; –0.413 |
| $R[F^2 > 4\sigma(F^2)]$ | 0.032 | 0.035 | 0.028 | 0.031 |
| w$R(F^2)$ | 0.079 | 0.084 | 0.059 | 0.058 |
| Goodness-of-fit, $S$ | 1.053 | 1.064 | 1.026 | 1.026 |

**Table S1.** Continued.

| | **(*N*-4Ito-3Ipy)**Cl | **(*N*-4Ito-3Brpy)**Cl · $H_2O$ | **(*N*-4Brto-3Ipy)**Cl | **(*N*-4Brto-3Brpy)**Cl · 1.5 $H_2O$ |
|---|---|---|---|---|
| Molecular formula | $C_{12}H_{10}NI_2Cl$ | $C_{12}H_{12}NOIBrCl$ | $C_{12}H_{10}NIBrCl$ | $C_{12}H_{13}O_{1.5}NBr_2Cl$ |
| $M_r$ | 457.48 | 856.97 | 410.47 | 363.48 |
| Crystal system | monoclinic | monoclinic | monoclinic | monoclinic |
| Space group | $P\,2_1/c$ | $I\,2/a$ | $P\,2_1/c$ | $P\,2_1/c$ |
| Crystal data: | | | | |
| $a$ / Å | 6.2399(2) | 12.5603(3) | 6.3220(2) | 15.4052(7) |
| $b$ / Å | 7.3769(3) | 15.6490(4) | 7.1428(2) | 16.1870(6) |
| $c$ / Å | 30.162(1) | 14.9991(4) | 29.7356(8) | 12.3295(6) |
| $\alpha$ / ° | 90 | 90 | 90 | 90 |
| $\beta$ / ° | 92.095(3) | 107.007(2) | 90.541(3) | 110.208(5) |
| $\gamma$ / ° | 90 | 90 | 90 | 90 |
| $V$ / Å$^3$ | 1387.46 | 2819.24 | 1342.7 | 2885.28 |
| $Z$ | 4 | 4 | 4 | 4 |
| $D_{calc}$ / g cm$^{-3}$ | 2.19 | 2.02 | 2.03 | 1.80 |
| $\lambda$(Mo$K_\alpha$) / Å | 0.71073 | 0.71073 | 0.71073 | 0.71073 |
| $T$ / K | 170 | 170 | 170 | 170 |
| Crystal size / mm$^3$ | 0.20 x 0.08 x 0.05 | 0.14 x 0.04 x 0.03 | 0.18 x 0.06 x 0.04 | 0.33 x 0.24 x 0.22 |
| $\mu$ / mm$^{-1}$ | 4.700 | 5.280 | 5.533 | 5.793 |
| $F$(000) | 848 | 1632 | 776 | 1528 |
| Refl. collected/unique | 16589 / 4377 | 16010 / 3760 | 16673 / 3899 | 25700 / 6291 |
| Data/restraints/ parameters | 145 | 172 | 145 | 325 |
| $\Delta\rho_{max}$ , $\Delta\rho_{min}$ / e Å$^{-3}$ | 0.908; –0.792 | 0.504; –0.929 | 0.662; –0.486 | 0.779; –1.021 |
| $R[F^2 > 4\sigma(F^2)]$ | 0.030 | 0.022 | 0.028 | 0.042 |
| w$R(F^2)$ | 0.064 | 0.054 | 0.053 | 0.101 |
| Goodness-of-fit, $S$ | 0.977 | 1.049 | 1.040 | 1.020 |

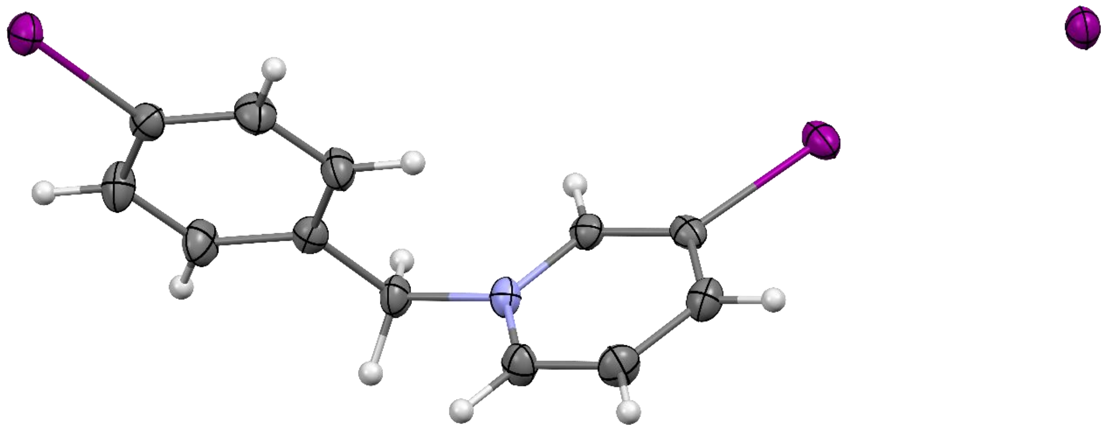

**Figure S1.** Molecular structure of **(*N*-4Ito-3Ipy)**I showing the atom-labelling scheme. Displacement ellipsoids are drawn at the 50 % probability level, and H atoms are shown as small spheres of arbitrary radius.

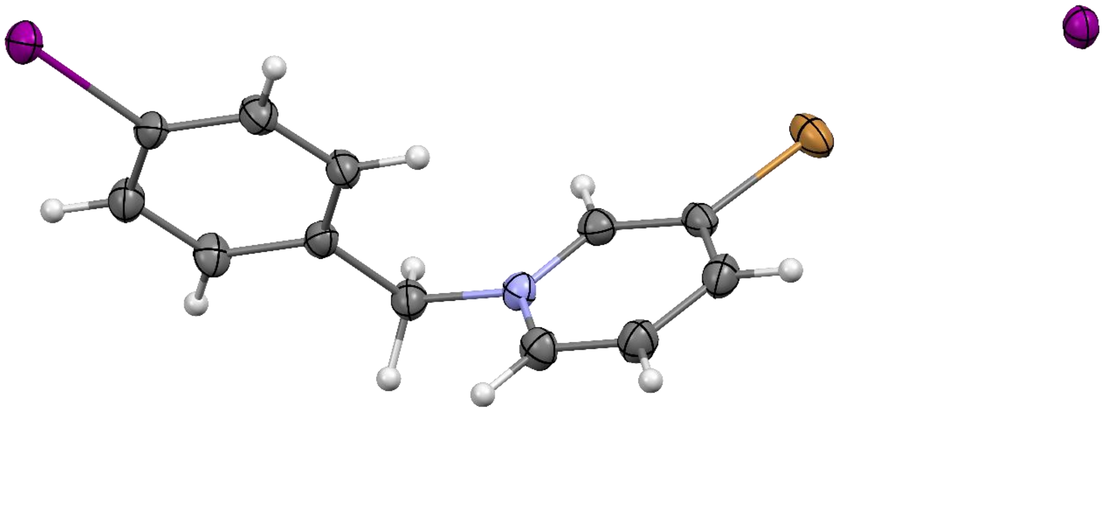

**Figure S2.** Molecular structure of **(*N*-4Ito-3Brpy)**I showing the atom-labelling scheme. Displacement ellipsoids are drawn at the 50 % probability level, and H atoms are shown as small spheres of arbitrary radius.

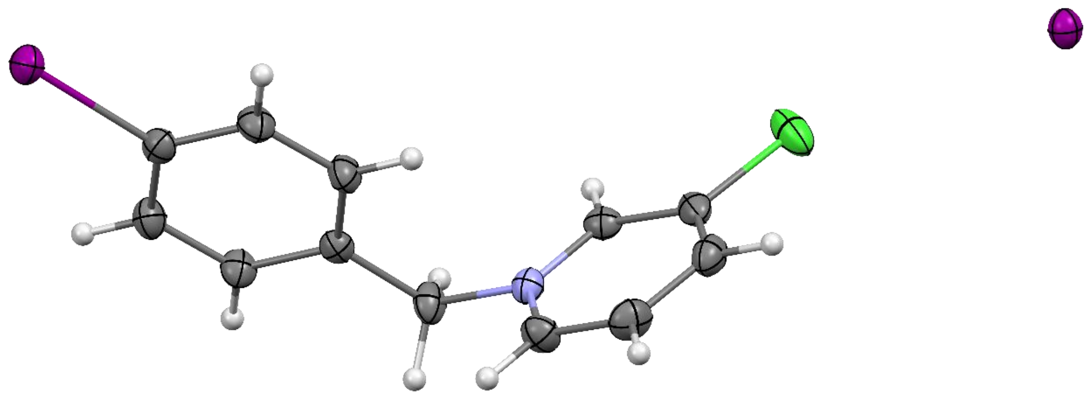

**Figure S3.** Molecular structure of **(*N*-4Ito-3Clpy)**I showing the atom-labelling scheme. Displacement ellipsoids are drawn at the 50 % probability level, and H atoms are shown as small spheres of arbitrary radius.

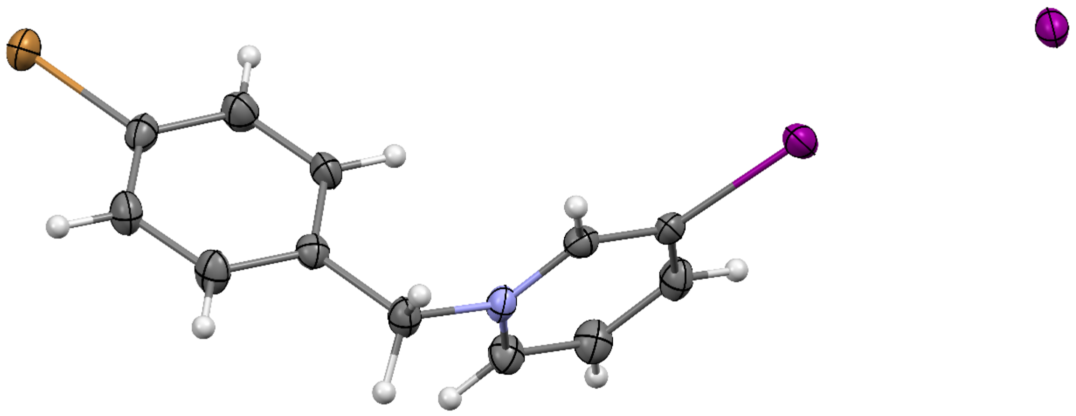

**Figure S4.** Molecular structure of **(*N*-4Brto-3Ipy)**I showing the atom-labelling scheme. Displacement ellipsoids are drawn at the 50 % probability level, and H atoms are shown as small spheres of arbitrary radius.

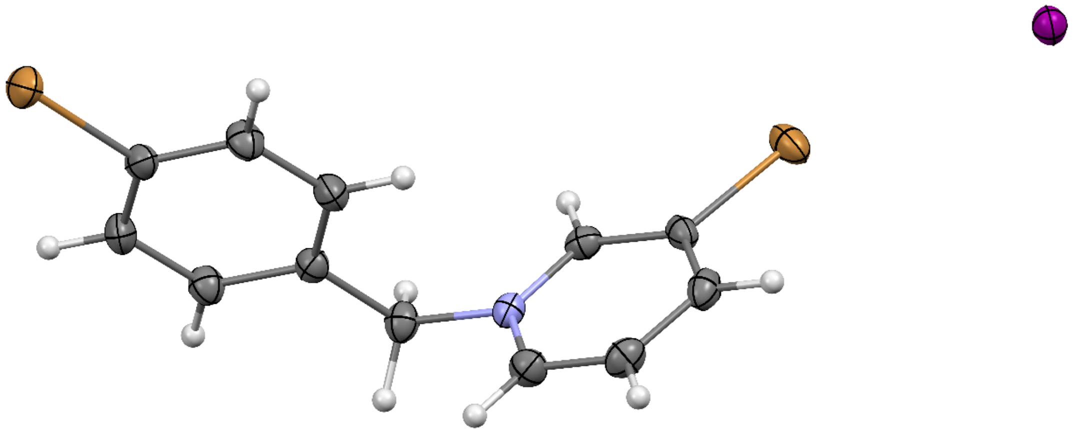

**Figure S5.** Molecular structure of **(*N*-4Brto-3Brpy)**I showing the atom-labelling scheme. Displacement ellipsoids are drawn at the 50 % probability level, and H atoms are shown as small spheres of arbitrary radius.

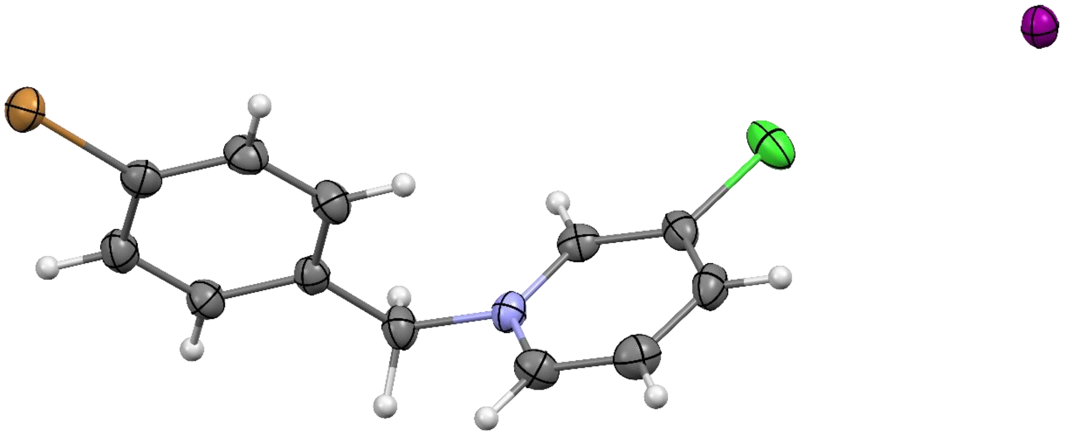

**Figure S6.** Molecular structure of **(*N*-4Brto-3Clpy)**I showing the atom-labelling scheme. Displacement ellipsoids are drawn at the 50 % probability level, and H atoms are shown as small spheres of arbitrary radius.

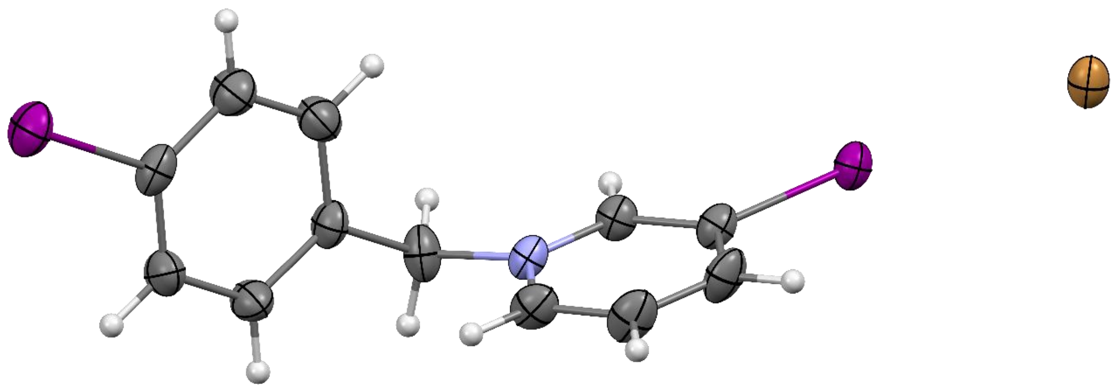

**Figure S7.** Molecular structure of **(*N*-4Ito-3Ipy)**Br showing the atom-labelling scheme. Displacement ellipsoids are drawn at the 50 % probability level, and H atoms are shown as small spheres of arbitrary radius.

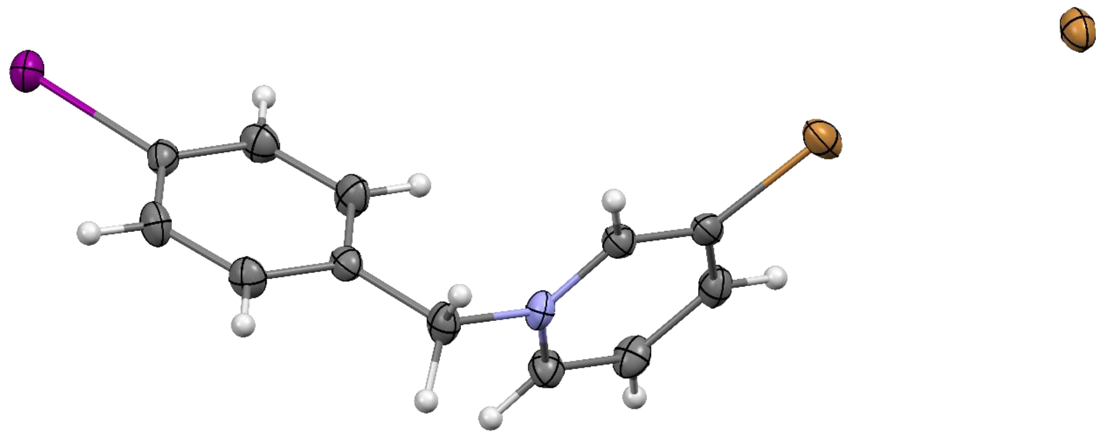

**Figure S8.** Molecular structure of **(*N*-4Ito-3Brpy)**Br showing the atom-labelling scheme. Displacement ellipsoids are drawn at the 50 % probability level, and H atoms are shown as small spheres of arbitrary radius.

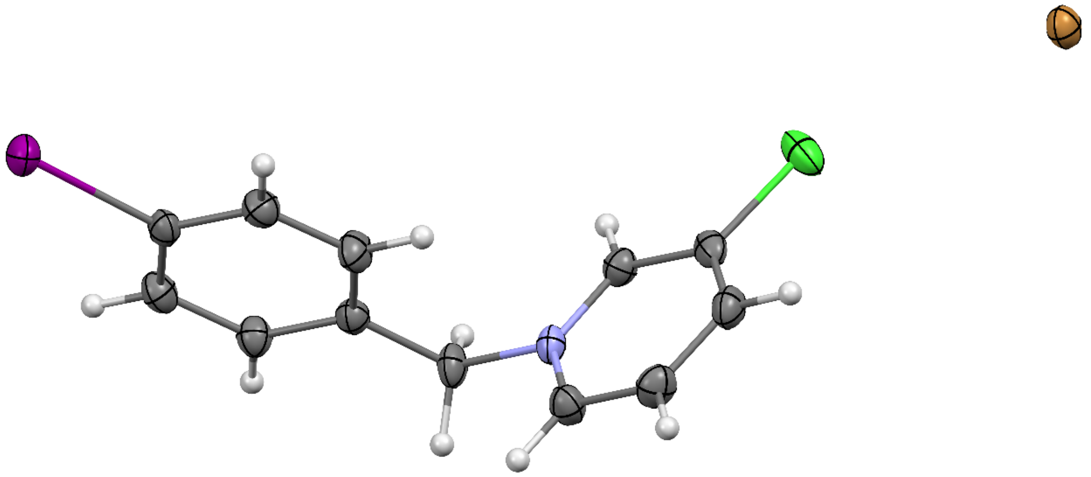

**Figure S9.** Molecular structure of **(*N*-4Ito-3Clpy)**Br showing the atom-labelling scheme. Displacement ellipsoids are drawn at the 50 % probability level, and H atoms are shown as small spheres of arbitrary radius.

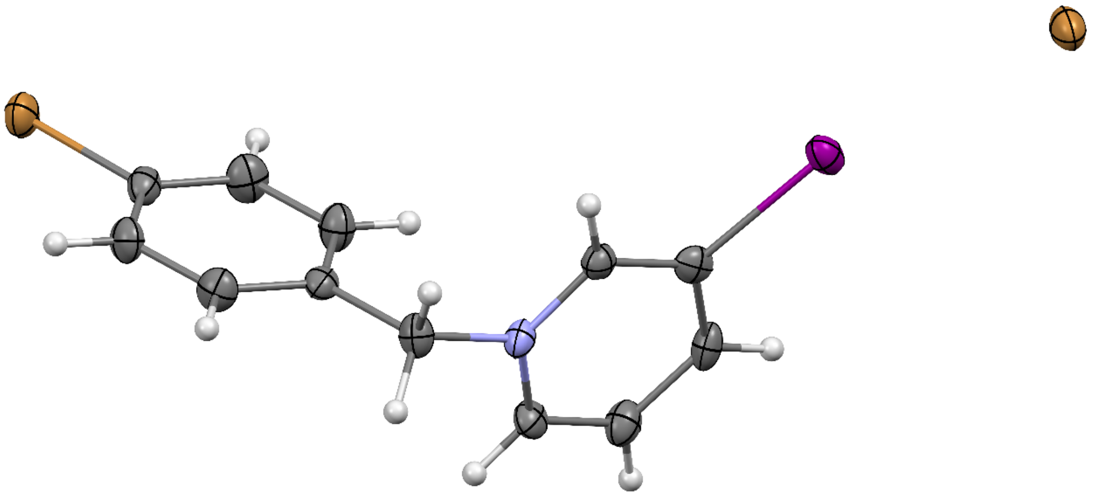

**Figure S10.** Molecular structure of **(*N*-4Brto-3Ipy)**Br showing the atom-labelling scheme. Displacement ellipsoids are drawn at the 50 % probability level, and H atoms are shown as small spheres of arbitrary radius.

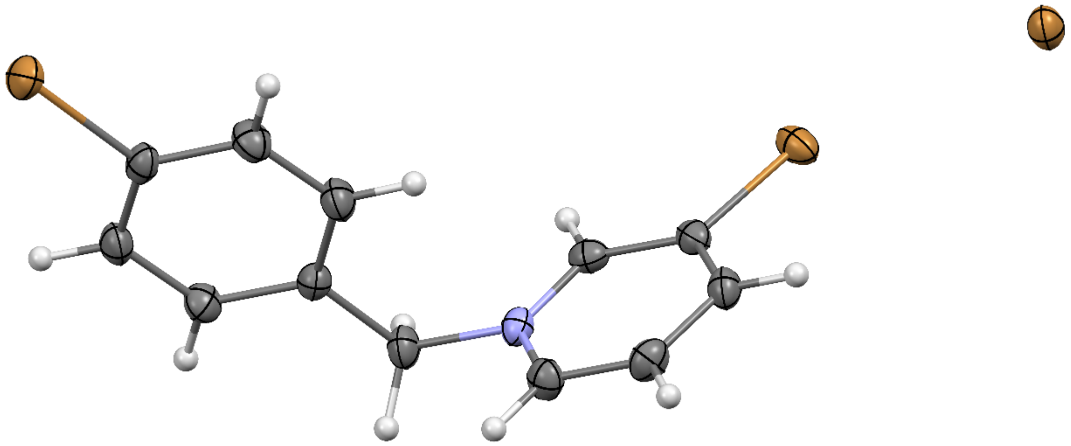

**Figure S11.** Molecular structure of **(*N*-4Brto-3Brpy)**Br showing the atom-labelling scheme. Displacement ellipsoids are drawn at the 50 % probability level, and H atoms are shown as small spheres of arbitrary radius.

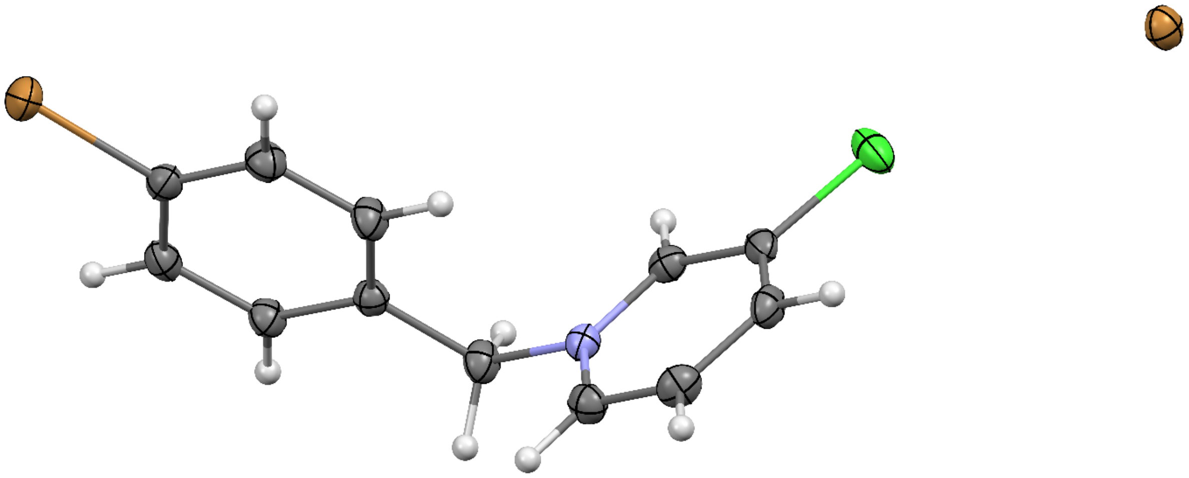

**Figure S12.** Molecular structure of **(*N*-4Brto-3Clpy)**Br showing the atom-labelling scheme. Displacement ellipsoids are drawn at the 50 % probability level, and H atoms are shown as small spheres of arbitrary radius.

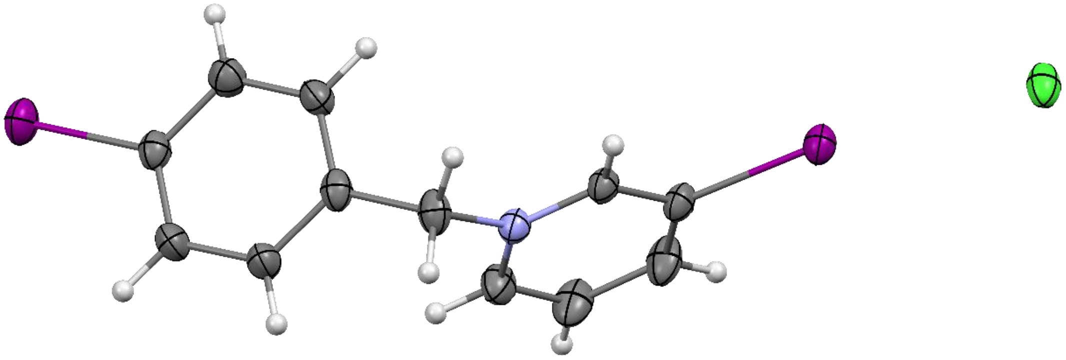

**Figure S13.** Molecular structure of **(*N*-4Ito-3Ipy)**Cl showing the atom-labelling scheme. Displacement ellipsoids are drawn at the 50 % probability level, and H atoms are shown as small spheres of arbitrary radius.

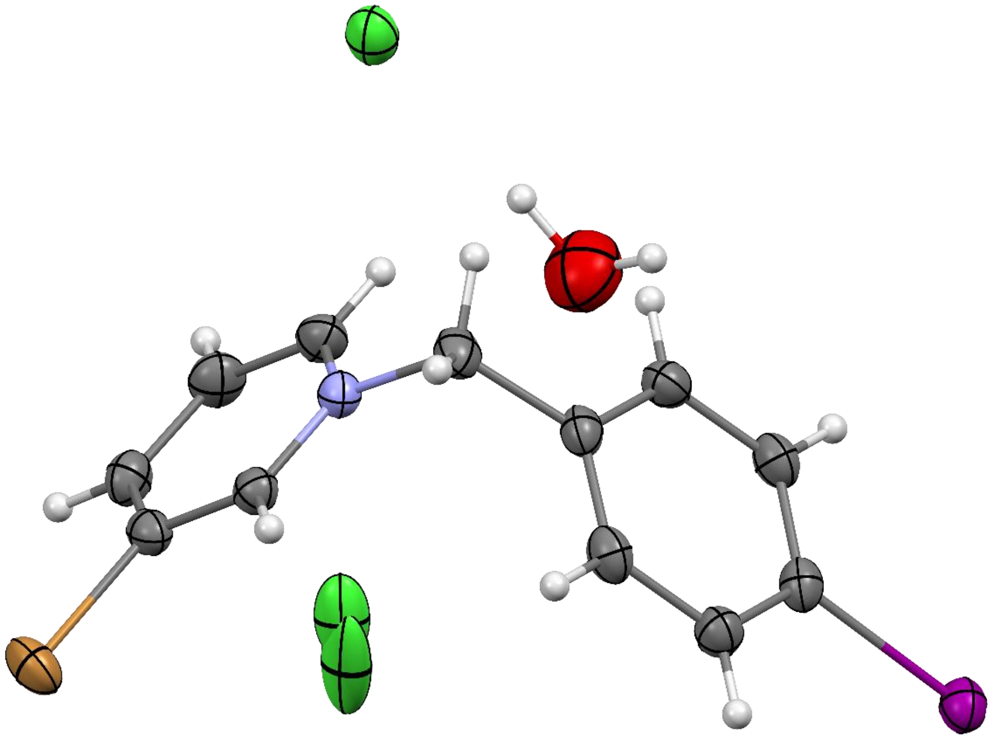

**Figure S14.** Molecular structure of **(*N*-4Ito-3Brpy)**Cl showing the atom-labelling scheme. Displacement ellipsoids are drawn at the 50 % probability level, and H atoms are shown as small spheres of arbitrary radius.

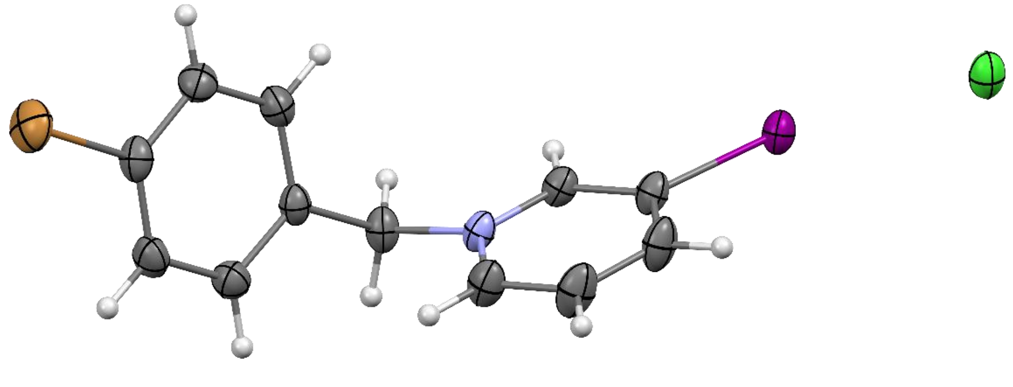

**Figure S15.** Molecular structure of **(*N*-4Brto-3Ipy)Cl** showing the atom-labelling scheme. Displacement ellipsoids are drawn at the 50 % probability level, and H atoms are shown as small spheres of arbitrary radius.

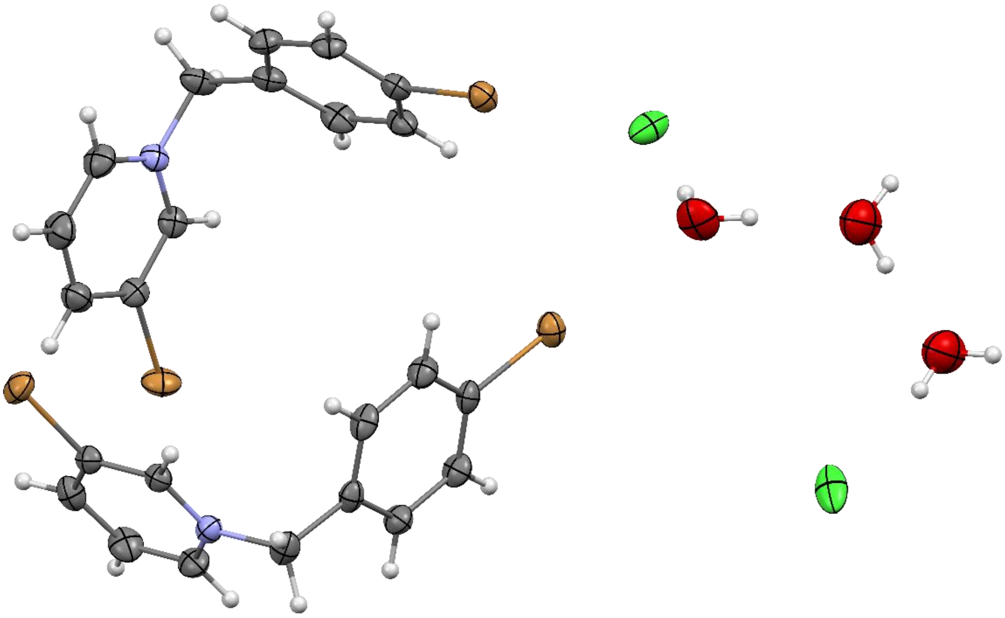

**Figure S16.** Molecular structure of **(*N*-4Brto-3Brpy)**Cl showing the atom-labelling scheme. Displacement ellipsoids are drawn at the 50 % probability level, and H atoms are shown as small spheres of arbitrary radius.

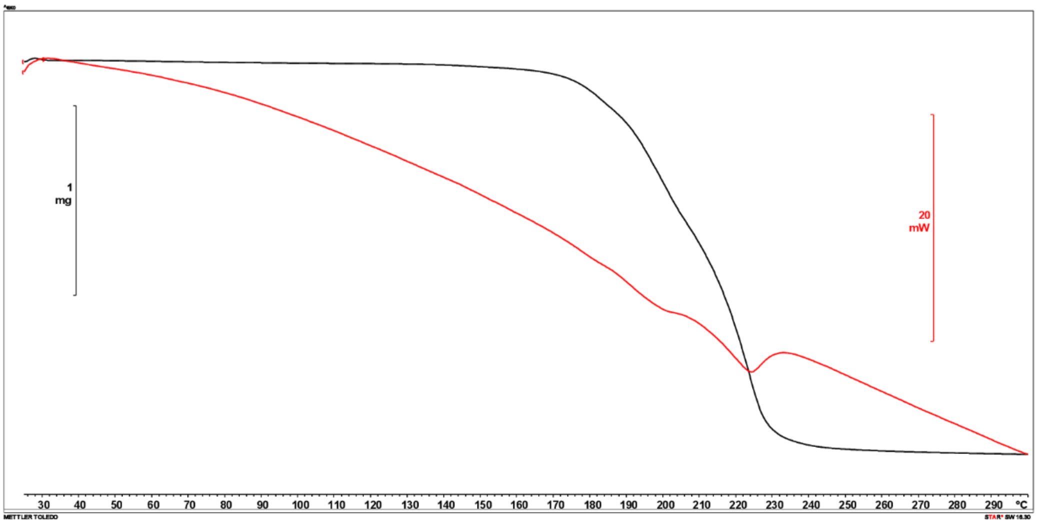


**Figure S17.** TG (black) and DSC (red) thermograms of **(*N*-4Ito-3Ipy)**I.

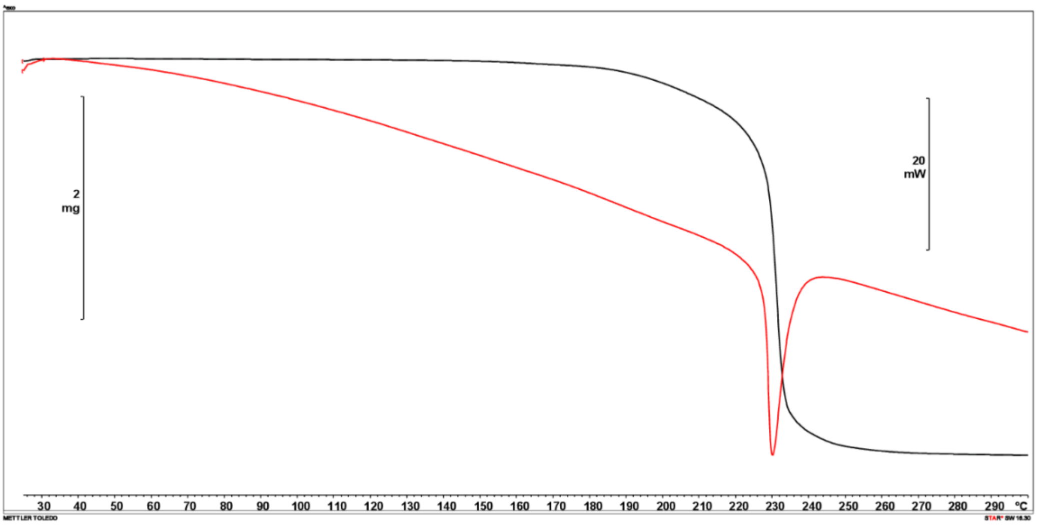


**Figure S18.** TG (black) and DSC (red) thermograms of **(*N*-4Ito-3Brpy)**I.

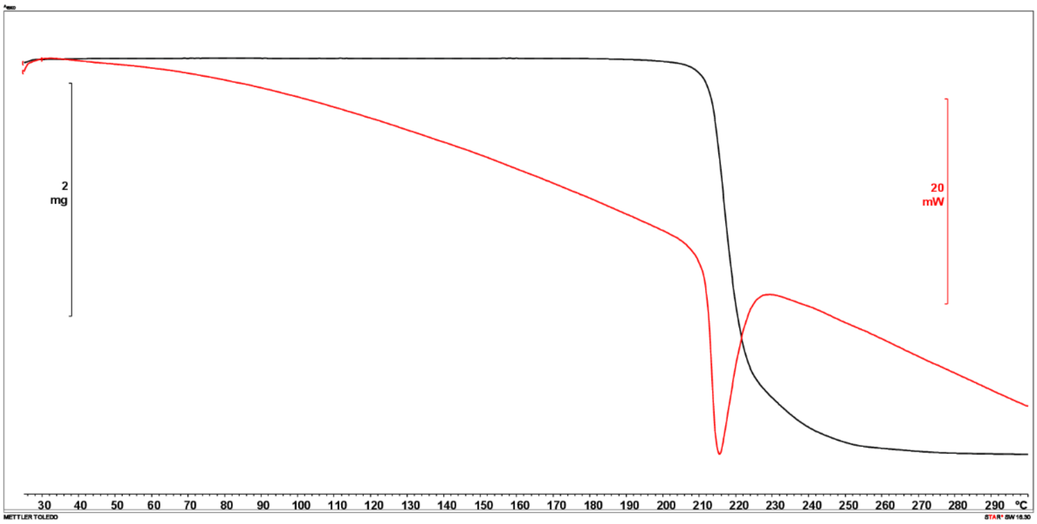


**Figure S19.** TG (black) and DSC (red) thermograms of **(*N*-4Ito-3Clpy)**I.

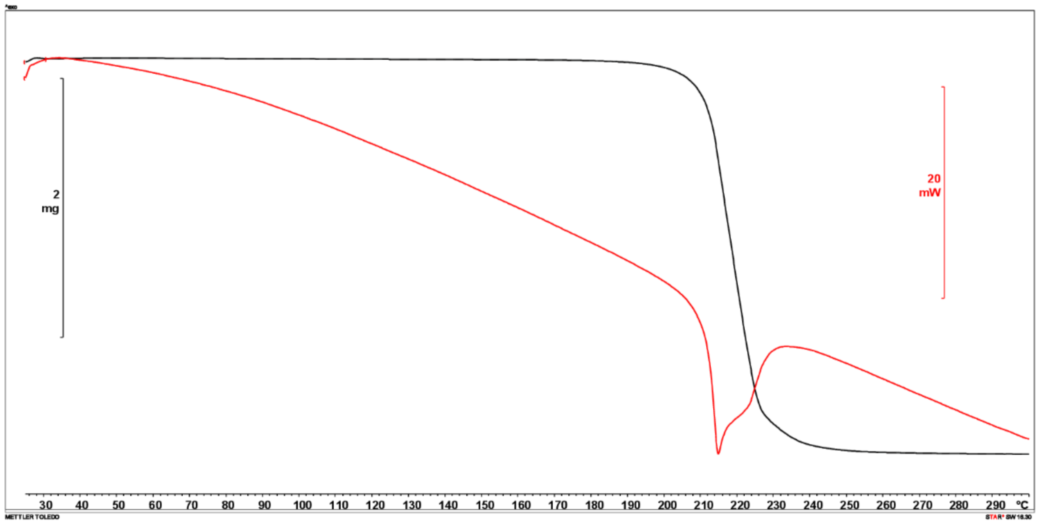


**Figure S20.** TG (black) and DSC (red) thermograms of **(*N*-4Brto-3Ipy)**I.

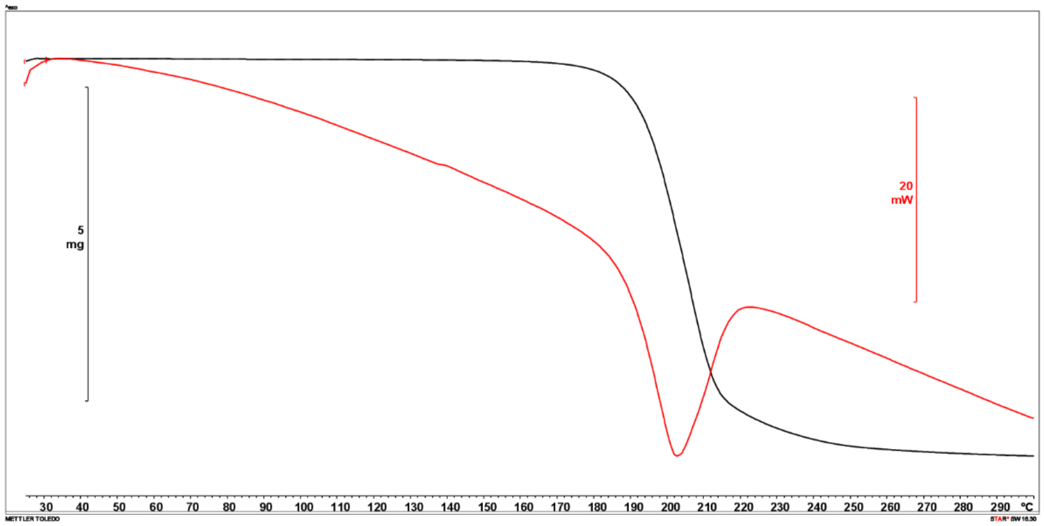


**Figure S21.** TG (black) and DSC (red) thermograms of **(*N*-4Brto-3Brpy)**I.

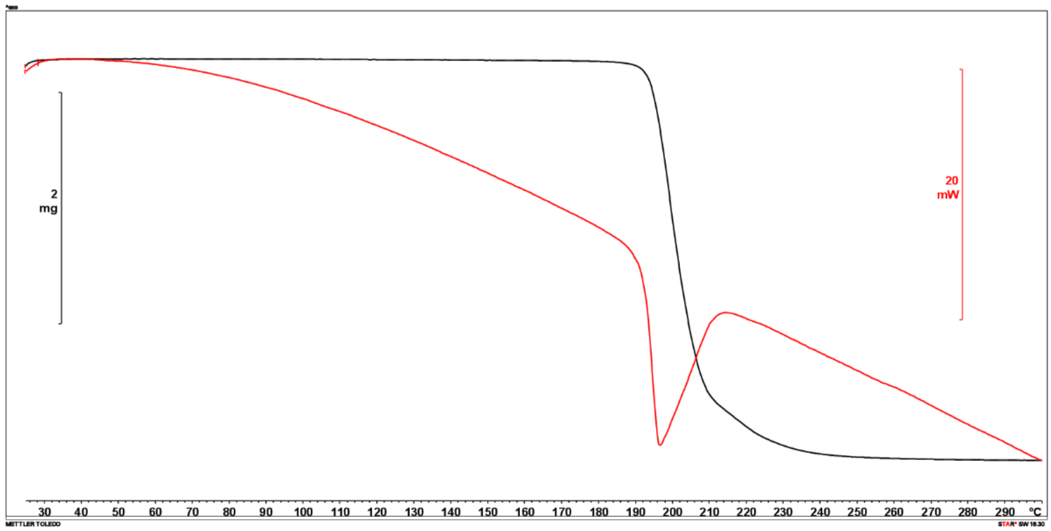


**Figure S22.** TG (black) and DSC (red) thermograms of **(*N*-4Brto-3Clpy)**I.

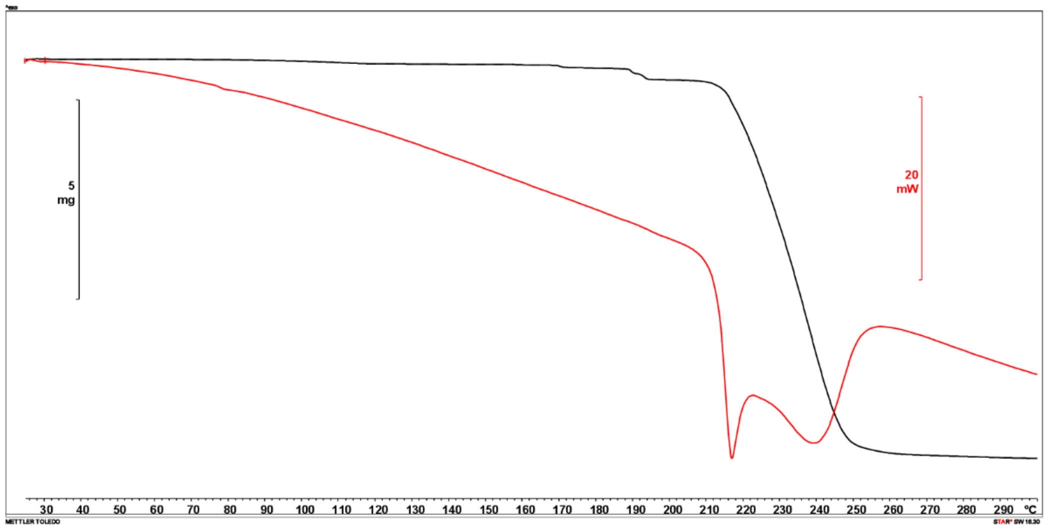


**Figure S23.** TG (black) and DSC (red) thermograms of **(*N*-4Ito-3Ipy)**Br.

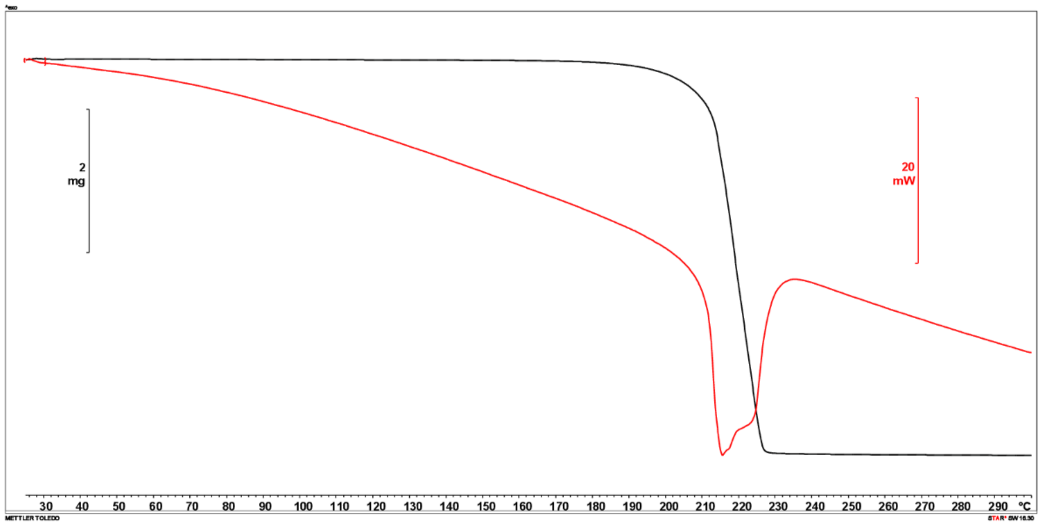


**Figure S24.** TG (black) and DSC (red) thermograms of **(*N*-4Ito-3Brpy)**Br.

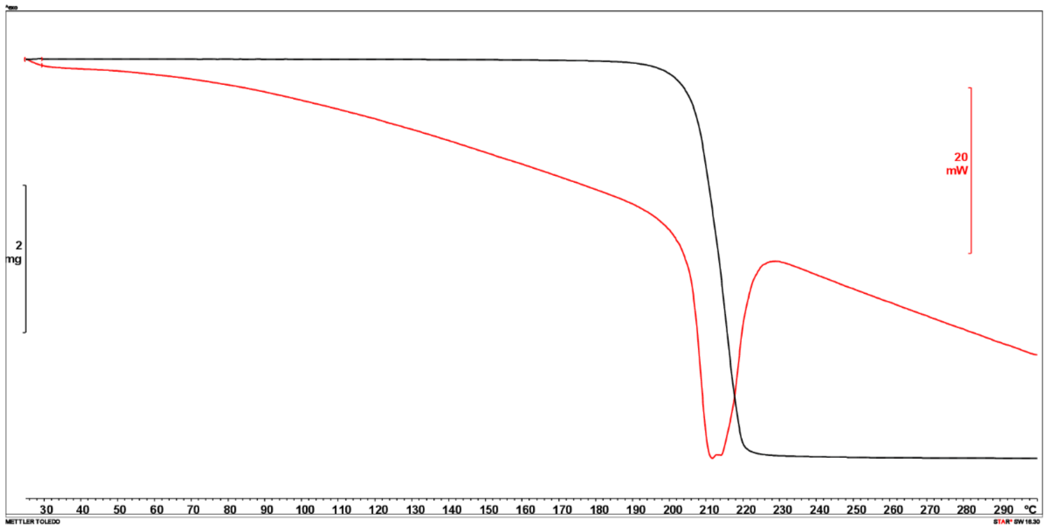


**Figure S25.** TG (black) and DSC (red) thermograms of **(*N*-4Ito-3Clpy)**Br.

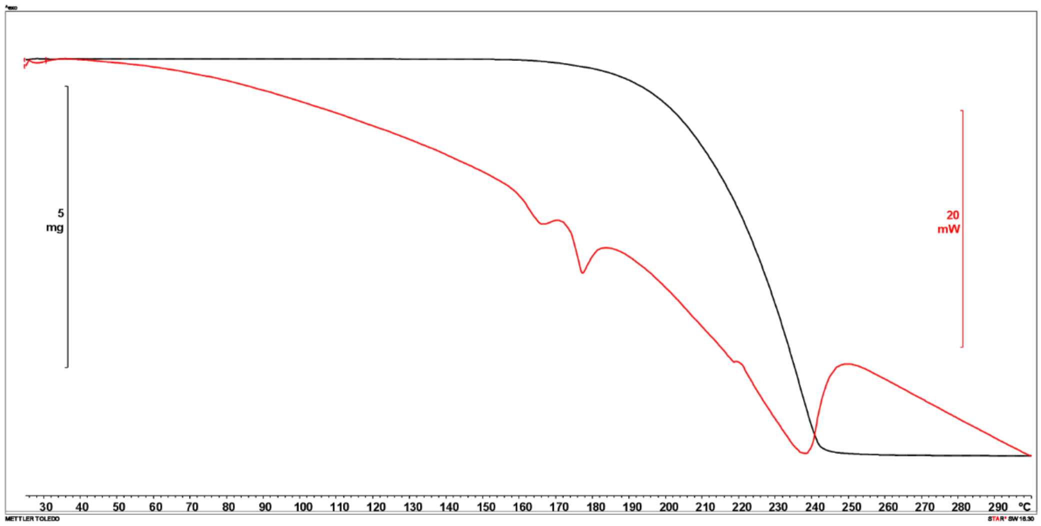


**Figure S26.** TG (black) and DSC (red) thermograms of **(*N*-4Brto-3Ipy)**Br.

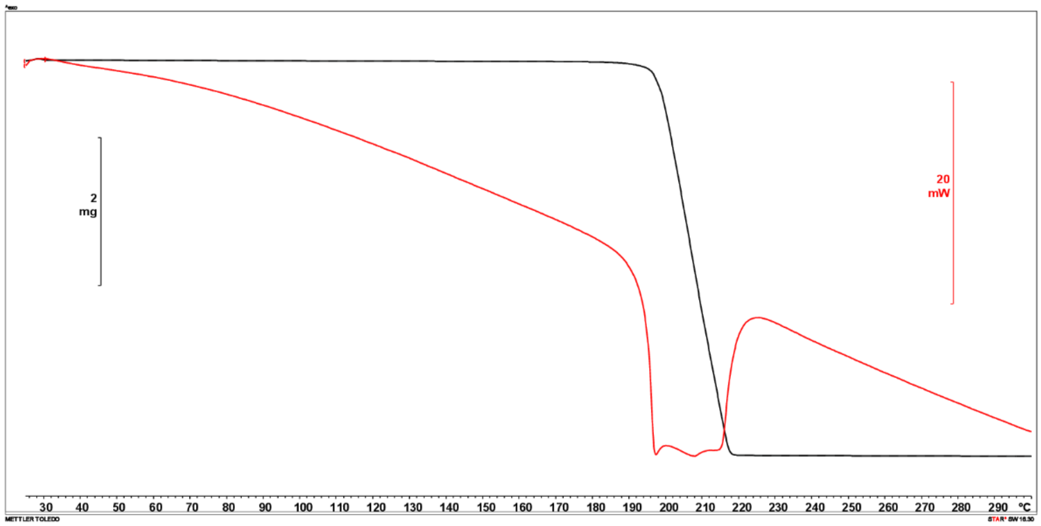


**Figure S27.** TG (black) and DSC (red) thermograms of **(*N*-4Brto-3Brpy)**Br.

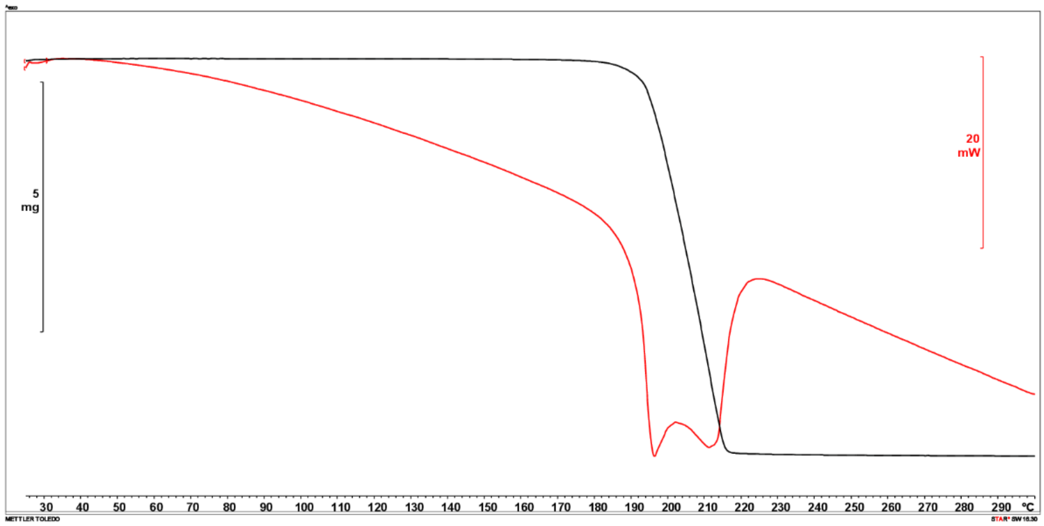


**Figure S28.** TG (black) and DSC (red) thermograms of **(*N*-4Brto-3Clpy)**Br.

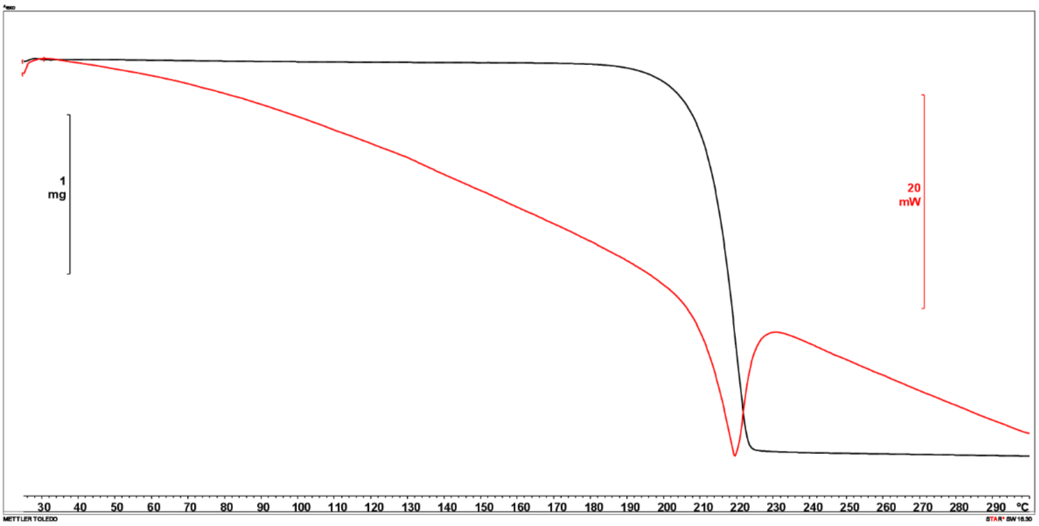


**Figure S29.** TG (black) and DSC (red) thermograms of **(*N*-4Ito-3Ipy)**Cl.

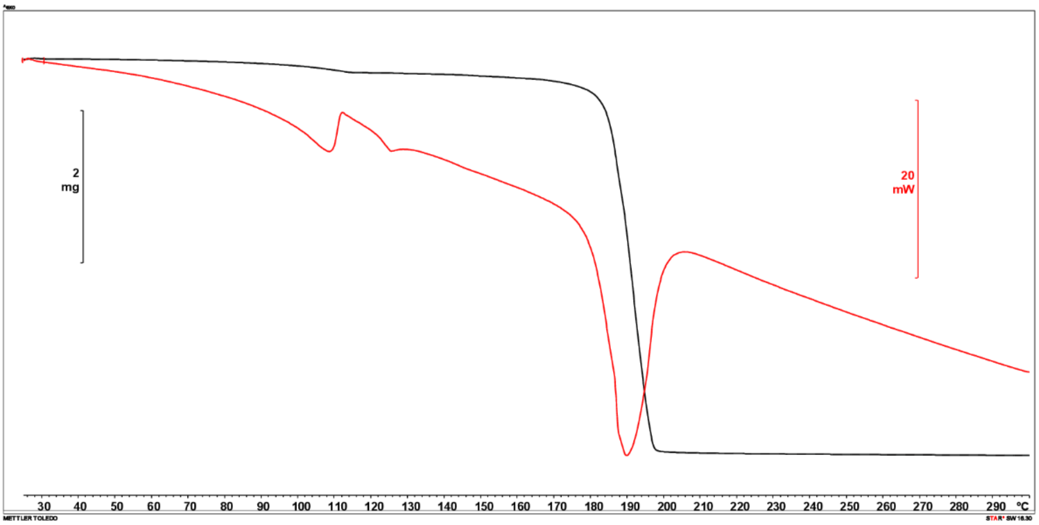


**Figure S30.** TG (black) and DSC (red) thermograms of **(*N*-4Ito-3Brpy)**Cl $H_2O$.

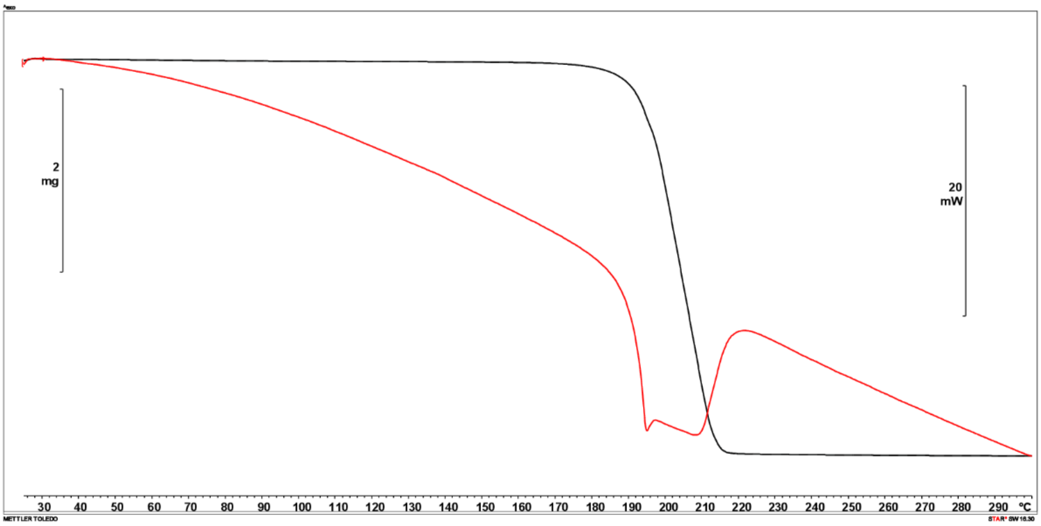


**Figure S31.** TG (black) and DSC (red) thermograms of **(*N*-4Brto-3Ipy)**Cl · $H_2O$.

**Table S2.** Halogen bond lengths, the corresponding relative bond lenght (*d*$_{rel}$) and angles in studyed *N*-(4-halogenobenzyl)-3-halogenopyridinium halogenides.

| | salt | *d* ($X^2\cdots(X^3)^-$) / Å | *d*$_{rel}$ * ($(X^2\cdots(X^3)^-)$) / % | $\varphi_1$ (C2–$X^2\cdots(X^3)^-$ / ° | *d* ($X^1\cdots(X^3)^-$) / Å | *d*$_{rel}$ ($(X^1\cdots(X^3)^-)$) / % | $\varphi_2$ (C10–$X^1\cdots(X^3)^-$ / ° |
|---|---|---|---|---|---|---|---|
| Type I | **III** | 3.539(4) | 85.49% | 166.25(8) | 3.828(4) | 92.47% | 163.52(1) |
| | **IBrI** | 3.625(6) | 90.40% | 161.66(2) | 3.822(5) | 92.31% | 163.78(1) |
| | **IClI** | 3.766(8) | 96.33% | 157.11(9) | 3.797(3) | 91.71% | 164.40(7) |
| | **BrII** | 3.532(4) | 85.31% | 166.16(9) | 3.841(4) | 95.78% | 163.06(1) |
| | **BrBrI** | 3.617(4) | 90.21% | 161.66(9) | 3.816(4) | 95.17% | 164.10(9) |
| | **BrClI** | 3.745(1) | 95.78% | 157.36(1) | 3.781(4) | 94.29% | 164.60(1) |
| | **IBrBr** | 3.451(4) | 90.82% | 161.33(8) | 3.799(3) | 96.66% | 159.99(7) |
| | **IClBr** | 3.609(9) | 97.55% | 155.95(1) | 3.755(4) | 95.54% | 160.45(9) |
| | **BrIBr** | 3.317(5) | 84.40% | 166.62(1) | 3.839(6) | 101.02% | 158.68(1) |
| | **BrBrBr** | 3.434(4) | 90.38% | 161.38(1) | 3.824(5) | 100.62% | 159.99(1) |
| | **BrClBr** | 3.580(6) | 96.76% | 156.65(7) | 3.764(3) | 99.04% | 160.67(6) |
| Type II | **IIBr** | 3.322(8) | 84.53% | 171.95(1) | 3.537(8) | 90.00% | 157.10(1) |
| | **IICl** | 3.177(9) | 83.84% | 171.51(8) | 3.500(9) | 92.35% | 151.69(8) |
| | **BrICl** | 3.174(8) | 83.75% | 170.57(7) | 3.577(8) | 97.74% | 144.77(7) |

* $d_{rel} = 100 * d\ (X^1/X^2\cdots(X^3)^-)/[r_{ion}(X^3)^- + r_{vdw}(X^1/X^2)])$

**Table S3.** C–H···($X^3$)$^-$ hydrogen bond lengths and angles in studyed *N*-(4-halogenobenzyl)-3-halogenopyridinium halogenides.

| Salt | Carbon | Hydrogen | Anion (X$^-$) | *d* (C–H···X$^-$) / Å | ∠ (C–H··· X$^-$) / ° |
|---|---|---|---|---|---|
| **III** | C5 | H5 | I3 | 3.854(4) | 109.5 |
| | C6 | H6A | I3 | 4.054(3) | 147.5 |
| | C6 | H6B | I3 | 4.159(4) | 150.4 |
| | C1 | H1 | I3 | 4.024(4) | 133.7 |
| | C3 | H3 | I3 | 3.885(4) | 146.4 |
| | C11 | H11 | I3 | 3.902(4) | 124 |
| | C12 | H12 | I3 | 3.954(4) | 121 |
| **IBrI** | C5 | H5 | I2 | 3.768(5) | 110.2 |
| | C6 | H6A | I2 | 3.991(5) | 147.4 |
| | C6 | H6B | I2 | 4.031(6) | 146.9 |
| | C1 | H1 | I2 | 3.898(6) | 130.9 |
| | C3 | H3 | I2 | 3.847(5) | 147.9 |
| | C11 | H11 | I2 | 3.879(6) | 126.8 |
| | C12 | H12 | I2 | 3.939(6) | 122.3 |
| **IClI** | C5 | H5 | I2 | 3.722(3) | 111.3 |
| | C6 | H6A | I2 | 3.965(3) | 147.9 |
| | C6 | H6B | I2 | 3.992(2) | 144.4 |
| | C1 | H1 | I2 | 3.789(3) | 127.8 |
| | C3 | H3 | I2 | 3.839(3) | 146.7 |
| | C11 | H11 | I2 | 3.871(3) | 126.6 |
| | C12 | H12 | I2 | 3.928(3) | 122.7 |
| **BrII** | C5 | H5 | I2 | 3.802(4) | 109 |
| | C6 | H6A | I2 | 4.015(3) | 145.9 |
| | C6 | H6B | I2 | 4.160(4) | 151.5 |
| | C1 | H1 | I2 | 4.009(4) | 133.3 |
| | C3 | H3 | I2 | 3.861(4) | 148.7 |
| | C11 | H11 | I2 | 3.903(4) | 120.8 |
| | C12 | H12 | I2 | 3.934(3) | 119.3 |
| **BrBrI** | C5 | H5 | I1 | 3.720(4) | 108.9 |
| | C6 | H6A | I1 | 3.951(3) | 146.4 |
| | C6 | H6B | I1 | 4.040(4) | 147.4 |
| | C1 | H1 | I1 | 3.867(3) | 130.4 |
| | C3 | H3 | I1 | 3.825(3) | 148.8 |
| | C11 | H11 | I1 | 3.886(3) | 122.9 |
| | C12 | H12 | I1 | 3.909(4) | 121.9 |
| **BrClI** | C5 | H5 | I1 | 3.675(4) | 109.4 |
| | C6 | H6A | I1 | 3.928(4) | 146.3 |
| | C6 | H6B | I1 | 3.995(4) | 144.4 |
| | C1 | H1 | I1 | 3.776(4) | 127.4 |

| | | | | | |
|---|---|---|---|---|---|
| | C3 | H3 | I1 | 3.812(4) | 148.8 |
| | C11 | H11 | I1 | 3.872(4) | 123.4 |
| | C12 | H12 | I1 | 3.897(4) | 122.2 |
| **IBrBr** | C5 | H5 | Br2 | 3.606(3) | 112.5 |
| | C6 | H6A | Br2 | 3.928(2) | 149.8 |
| | C6 | H6B | Br2 | 3.787(3) | 147.8 |
| | C1 | H1 | Br2 | 3.772(3) | 130.9 |
| | C3 | H3 | Br2 | 3.608(3) | 144.9 |
| | C11 | H11 | Br2 | 3.714(3) | 124.8 |
| | C12 | H12 | Br2 | 3.761(3) | 121.6 |
| **IClBr** | C5 | H5 | Br1 | 3.571(4) | 114.4 |
| | C6 | H6A | Br1 | 3.768(3) | 148.5 |
| | C6 | H6B | Br1 | 3.847(4) | 146.3 |
| | C1 | H1 | Br1 | 3.658(4) | 126.4 |
| | C3 | H3 | Br1 | 3.589(4) | 144.2 |
| | C11 | H11 | Br1 | 3.694(4) | 125.7 |
| | C12 | H12 | Br1 | 3.751(4) | 122.1 |
| **BrIBr** | C5 | H5 | Br2 | 3.658(4) | 109.1 |
| | C6 | H6A | Br2 | 3.830(4) | 145 |
| | C6 | H6B | Br2 | 4.118(5) | 154.1 |
| | C1 | H1 | Br2 | 3.955(4) | 133 |
| | C3 | H3 | Br2 | 3.623(4) | 146.9 |
| | C11 | H11 | Br2 | 3.736(5) | 119 |
| | C12 | H12 | Br2 | 3.744(6) | 119.2 |
| **BrBrBr** | C5 | H5 | Br3 | 3.574(3) | 109.5 |
| | C6 | H6A | Br3 | 3.762(3) | 145.5 |
| | C6 | H6B | Br3 | 3.907(4) | 149.4 |
| | C1 | H1 | Br3 | 3.756(4) | 129.6 |
| | C3 | H3 | Br3 | 3.591(3) | 147.2 |
| | C11 | H11 | Br3 | 3.712(3) | 121.3 |
| | C12 | H12 | Br3 | 3.723(3) | 121.3 |
| **BrClBr** | C5 | H5 | Br2 | 3.535(2) | 111.9 |
| | C6 | H6A | Br2 | 3.737(2) | 146.3 |
| | C6 | H6B | Br2 | 3.826(2) | 146.4 |
| | C1 | H1 | Br2 | 3.636(2) | 126.8 |
| | C3 | H3 | Br2 | 3.575(2) | 147.1 |
| | C11 | H11 | Br2 | 3.693(2) | 122.2 |
| | C12 | H12 | Br2 | 3.712(2) | 122 |

**Table S4.** Calculated electrostatic potentials products and halogen bond energies of studyed *N*-(4-halogenobenzyl)-3-halogenopyridinium halogenides.

| | salt | $-V_{max}(\mathbf{X}^1)*V(\mathbf{X}^3)$ / $10^6$ kJ$^2$ mol$^{-2}$ $e^{-2}$ | $-V_{max}(\mathbf{X}^2)*V(\mathbf{X}^3)$ / $10^6$ kJ$^2$ mol$^{-2}$ $e^{-2}$ | $E$ ($\mathbf{X}^1\cdots(\mathbf{X}^3)^-$) / kJ mol$^{-1}$ | $E$ ($\mathbf{X}^2\cdots(\mathbf{X}^3)^-$) / kJ mol$^{-1}$ |
|---|---|---|---|---|---|
| Type I | **III** | 1.775661 | 2.427205 | -227.7 | -275.2 |
| | **IBrI** | 1.796023 | 2.237738 | -186.9 | -241.6 |
| | **IClI** | 1.78728 | 2.001153 | -189.0 | -219.6 |
| | **BrII** | 1.604469 | 2.429482 | -111.4 | -276.6 |
| | **BrBrI** | 1.618 | 2.229389 | -173.9 | -242.5 |
| | **BrClI** | 1.621825 | 2.006072 | -175.9 | -220.6 |
| | **IBrBr** | 1.977307 | 2.463606 | -183.4 | -254.4 |
| | **IClBr** | 1.967681 | 2.203141 | -174.2 | -227.9 |
| | **BrIBr** | 1.766418 | 2.674704 | -105.0 | -297.4 |
| | **BrBrBr** | 1.781315 | 2.454414 | -97.4 | -255.8 |
| | **BrClBr** | 1.785525 | 2.208557 | -205.1 | -229.5 |
| Type II | **IIBr** | 1.954889 | 2.672197 | -201.3 | -295.4 |
| | **IICl** | 2.103998 | 2.876019 | -204.4 | -311.58 |
| | **BrICl** | 1.901151 | 2.878717 | -184.6 | -313.1 |

**Table S5.** Calculated Kitaigorodsky packing coefficient (KPC) and other parameters describing the halogen bonded chains (defined on the Figure 7 in the main text) used for comparison of type I and type II structures of studyed *N*-(4-halogenobenzyl)-3-halogenopyridinium halogenides.

| | salt | **KPC** | $\tau_1$ / ° | $\tau_2$ / ° | $d_{cat}$ / Å | $d_{XXX}$ / Å | $d_{chain}$ / Å | $\Theta$ / ° |
|---|---|---|---|---|---|---|---|---|
| Type I | **III** | 67.4 | 11.87 | 104.65 | 6.847 | 10.09 | 16.62 | 124.39 |
| | **IBrI** | 67.7 | 10.49 | 107.28 | 6.894 | 10.017 | 16.633 | 125.41 |
| | **IClI** | 67.5 | 8.51 | 106.5 | 6.917 | 10.044 | 16.681 | 126.38 |
| | **BrII** | 67.9 | 14.32 | 102.24 | 6.795 | 9.888 | 16.356 | 124.05 |
| | **BrBrI** | 68.3 | 12.52 | 105.36 | 6.864 | 9.816 | 16.401 | 125.23 |
| | **BrClI** | 68.1 | 11.67 | 106.16 | 6.905 | 9.786 | 16.453 | 126.41 |
| | **IBrBr** | 69.8 | 6.41 | 102.05 | 6.802 | 9.751 | 16.23 | 124.48 |
| | **IClBr** | 70 | 4.5 | 100.84 | 6.798 | 9.686 | 16.312 | 124.42 |
| | **BrIBr** | 69.6 | 10.45 | 98.75 | 6.747 | 9.646 | 16.012 | 124.25 |
| | **BrBrBr** | 70.8 | 8.56 | 101.1 | 6.791 | 9.546 | 16.01 | 123.76 |
| | **BrClBr** | 70.9 | 7.6 | 101.71 | 6.8 | 9.487 | 16.009 | 123.96 |
| Type II | **IIBr** | 65.8 | 70.64 | 139.85 | 7.208 | 10.575 | 16.887 | 150.76 |
| | **IICl** | 67.4 | 74.97 | 137.78 | 7.178 | 10.425 | 16.581 | 153.2 |
| | **BrICl** | 67.7 | 78.43 | 136.11 | 7.143 | 10.291 | 16.203 | 152.04 |

**Table S6.** Mesured melting/sublimation point temepratures and enthapies of studyed *N*-(4-halogenobenzyl)-3-halogenopyridinium halogenides.

| | salt | T / °C | $\Delta H$ / kJ mol$^{-1}$ |
|---|---|---|---|
| Type I | **III** | 162.5 | -154.82 |
| | **IBrI** | 177 | -155.12 |
| | **IClI** | 202 | -115.79 |
| | **BrII** | 196.5 | -161.76 |
| | **BrBrI** | 169.5 | -132.96 |
| | **BrClI** | 184.5 | -110.74 |
| | **IBrBr** | 188.5 | -172.09 |
| | **IClBr** | 188 | -149.32 |
| | **BrIBr** | 169 | -120.7 |
| | **BrBrBr** | 182.5 | -162.84 |
| | **BrClBr** | 178.5 | -132.07 |
| Type II | **IIBr** | 204 | -156.92 |
| | **IICl** | 184.5 | -152.71 |
| | **BrICl** | 177.5 | -149.72 |

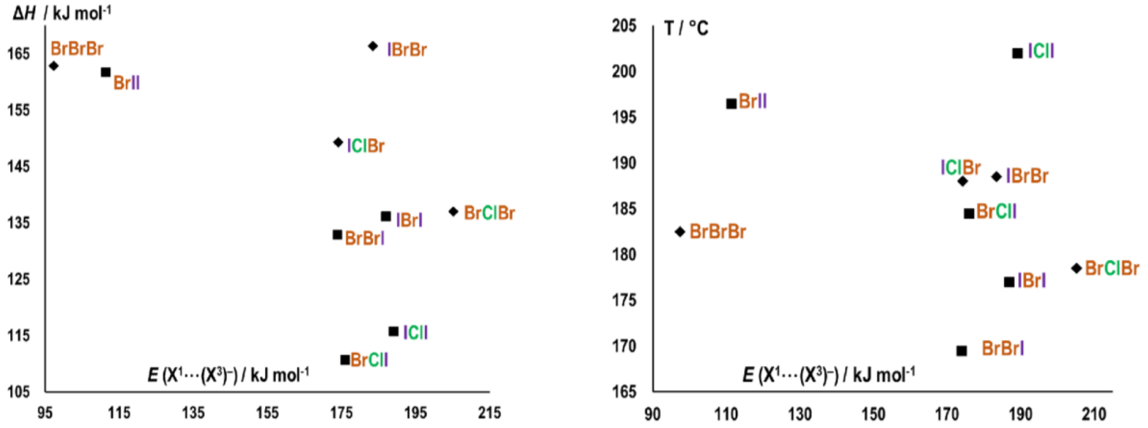


**Figure S32.** Correlation between the computed $\mathbf{X^1 \cdots (X^3)^-}$ halogen bond energies and: a) the evaporation enthalpies (**$\Delta H$**), b) onset temperatures (**T**) of the melting/evaporation within the type I structures.

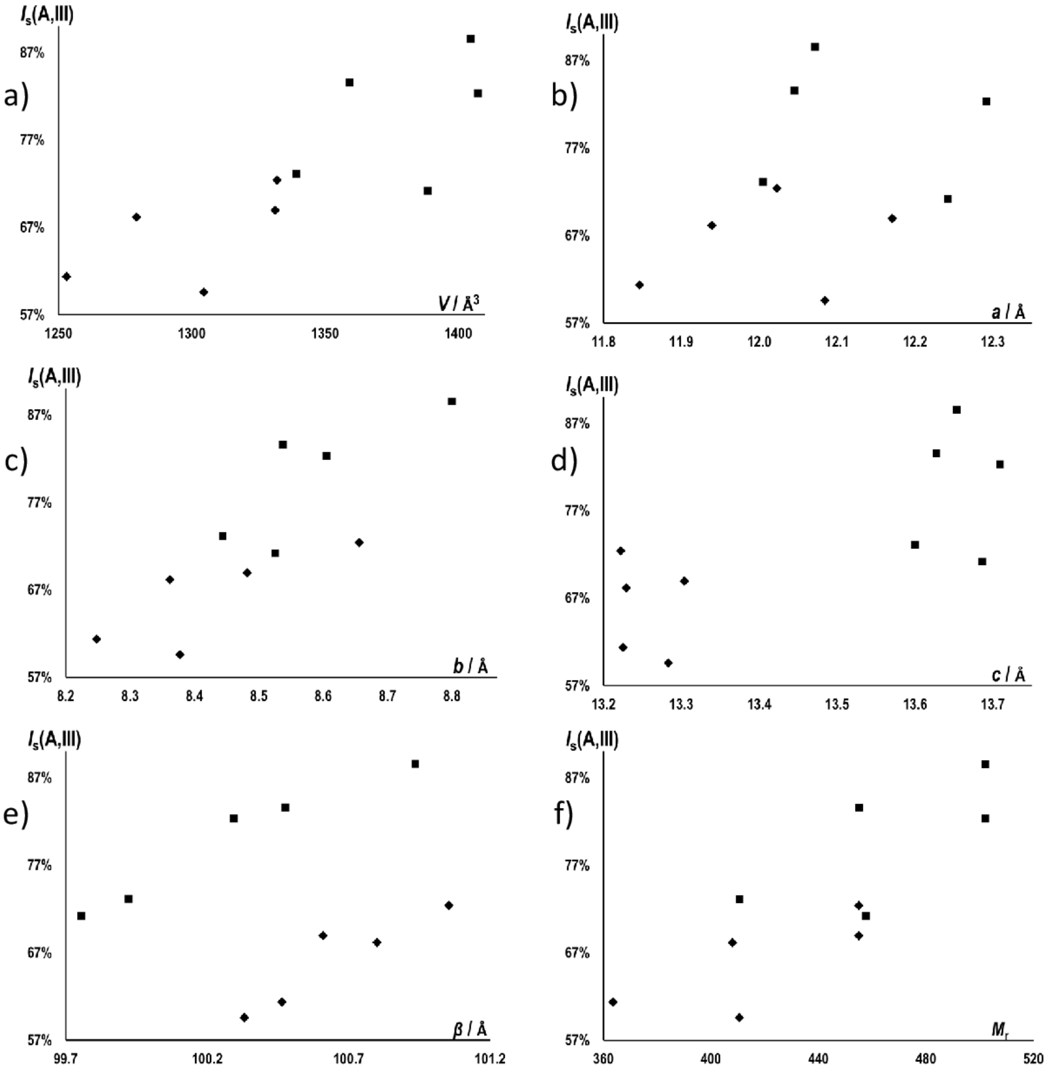


**Figure S33.** Correlation of $I_s$ and the unit cell parameters of type I structures; a) $I_s$ *vs.* $V$ plot, b) $I_s$ *vs.* $a$ plot, c) $I_s$ *vs.* $b$ plot, d) $I_s$ *vs.* $c$ plot, e) $I_s$ *vs.* $\beta$ plot, f) $I_s$ *vs.* $M_r$ plot. Structures of iodide salts are represented by squares, bromide salts as rhombi and chloride as triangles.

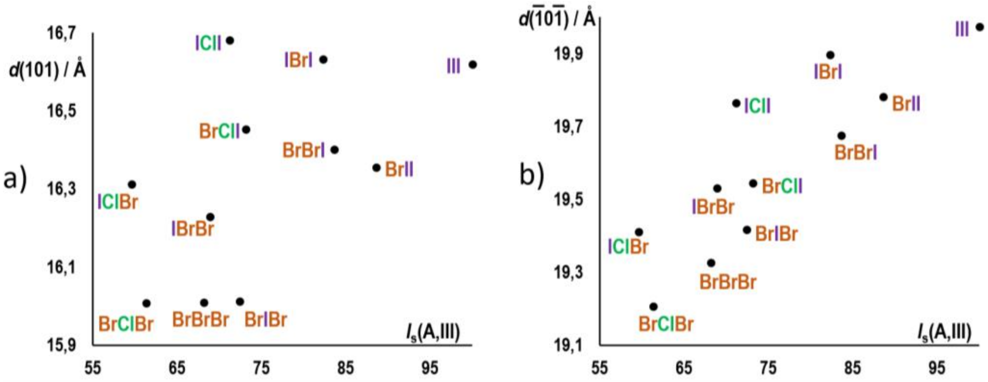


**Figure S34.** Plot of the distance between the a) [101] planes and b) [-10-1] planes *vs* isostructurality indices ($I_s$(A,III)) for the 11 crystals belonging to the structural type I.

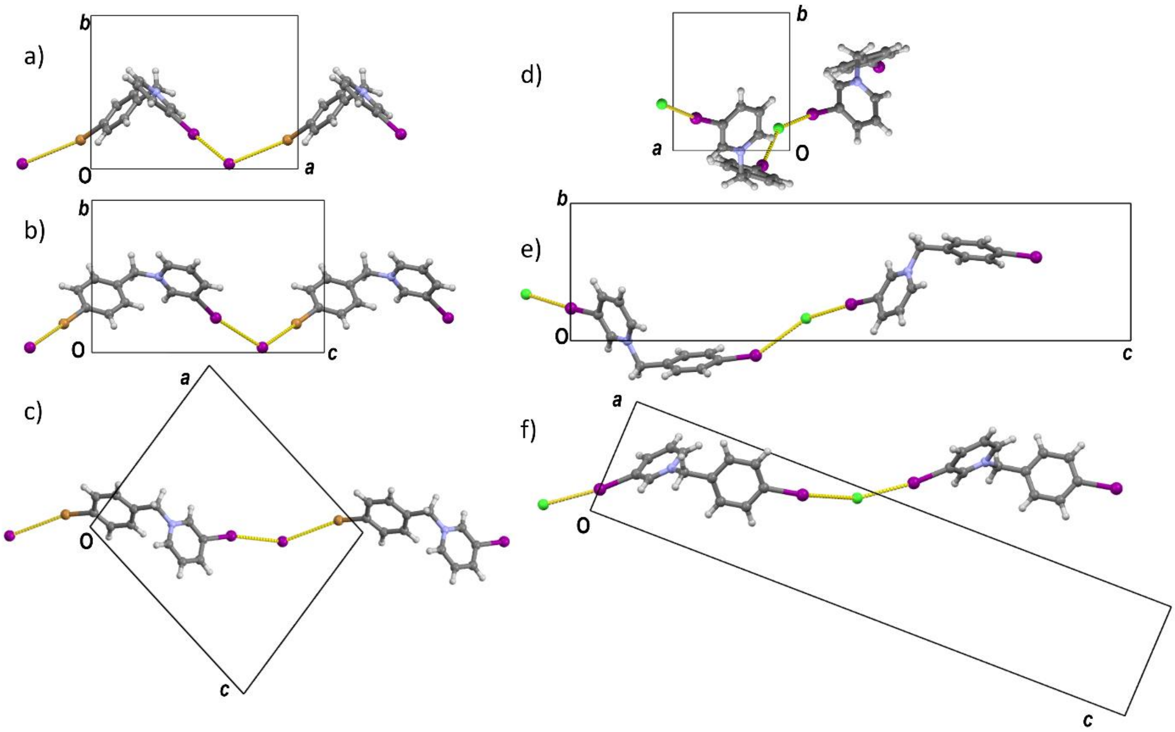


**Figure S35.** Halogen bonded chains within the unit cell in type I structures (in **BrII**; a), b) and c)) and type II structures (in **IICl**; d), e) and f)). Both structures viewed along: a), d) the crystallographic *c* axis; b), e) the crystallographic *a* axis; c), f) the crystallographic *b* axis.

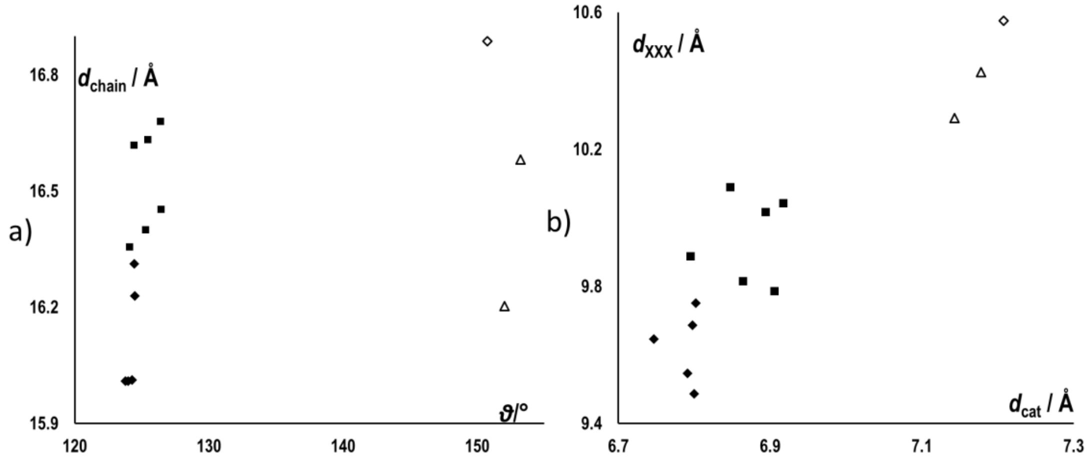


**Figure S36.** Parameters describing the halogen bonded chains in type I (black) and type II structures (white with black border): a) total length of a unit of the halogen bonded chain ($d_{chain}$) *vs* angle between the two halogen bonds ($\theta$) and b) length of the fragment of the halogen bonded chain which contains both halogen bonds ($d_{XXX}$) *vs* the length of the hydrocarbon skeleton of the cation ($d_{cat}$). Structures of iodide salts are represented by squares, bromide salts as rhombi and chloride as triangles.

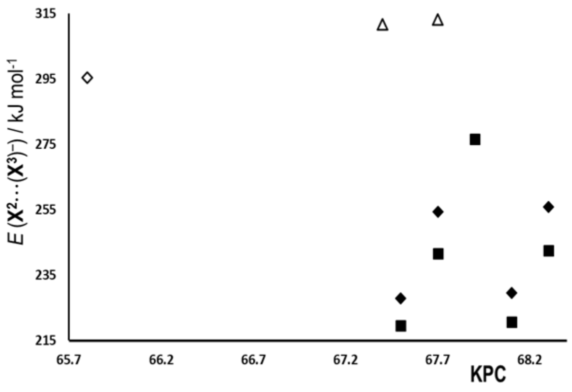


**Figure S37.** Distribution of the two isostructural series on a plot of the energies of ion pairs (computed *in vacuo* for geometries as found in the crystals, $E(\mathbf{X^2}\cdots(\mathbf{X^3})^-)$) *vs* the Kitaigorodsky packing coefficient (KPC).

[*N*-4Brto-3Brpy]$^+$

$E$ = -5565.22500872 a.u.

| Symbol | X | Y | Z |
|---|---|---|---|
| C | 2.0126880 | 2.0344010 | 0.3321070 |
| C | 1.0481700 | 1.0378520 | 0.4326950 |
| C | 2.6883160 | -0.6235250 | 0.1220260 |
| C | 3.6876020 | 0.3363990 | 0.0126070 |
| C | 3.3473210 | 1.6925030 | 0.1187460 |
| H | 1.7089390 | 3.0724870 | 0.4195340 |
| H | -0.0024370 | 1.2471740 | 0.5920860 |
| H | 2.8948440 | -1.6859950 | 0.0504630 |
| H | 4.1112140 | 2.4599690 | 0.0342150 |
| C | 0.3686320 | -1.3826390 | 0.4815890 |
| H | 0.6515120 | -2.1332120 | -0.2590650 |
| H | 0.5381220 | -1.7981090 | 1.4779840 |
| N | 1.3981530 | -0.2622040 | 0.3261380 |
| C | -1.0578030 | -0.9474410 | 0.3017970 |
| C | -1.8712930 | -0.6970650 | 1.4183510 |
| C | -1.6058030 | -0.8158330 | -0.9855850 |
| C | -3.2046910 | -0.3116890 | 1.2588840 |
| H | -1.4739610 | -0.8153670 | 2.4246360 |
| C | -2.9346540 | -0.4281120 | -1.1580240 |
| H | -1.0001210 | -1.0263750 | -1.8650780 |
| C | -3.7277830 | -0.1752210 | -0.0314400 |
| H | -3.8297440 | -0.1287360 | 2.1270470 |
| H | -3.3535180 | -0.3350400 | -2.1549120 |
| Br | 5.4717180 | -0.2130070 | -0.2807380 |
| Br | -5.5441790 | 0.3461740 | -0.2607590 |

[*N*-4Brto-3Ipy]$^{+}$

*E* = -10011.39653730 a.u.

| Symbol | X | Y | Z |
|---|---|---|---|
| C | 1.3513900 | 2.0918800 | 0.2895820 |
| C | 0.3995170 | 1.0832580 | 0.3854030 |
| C | 2.0773200 | -0.5558660 | 0.1687360 |
| C | 3.0707280 | 0.4123110 | 0.0659570 |
| C | 2.6970030 | 1.7648590 | 0.1275380 |
| H | 1.0295650 | 3.1270030 | 0.3408340 |
| H | -0.6585610 | 1.2792480 | 0.5072450 |
| H | 2.2935490 | -1.6182890 | 0.1318310 |
| H | 3.4444600 | 2.5488010 | 0.0486310 |
| C | -0.2393610 | -1.3483390 | 0.4662840 |
| H | 0.0536530 | -2.0875490 | -0.2819330 |
| H | -0.0643780 | -1.7716390 | 1.4583570 |
| N | 0.7743420 | -0.2124890 | 0.3227830 |
| C | -1.6720680 | -0.9324320 | 0.2912200 |
| C | -2.4828190 | -0.6840610 | 1.4103060 |
| C | -2.2278770 | -0.8139590 | -0.9940050 |
| C | -3.8207140 | -0.3131030 | 1.2552330 |
| H | -2.0791660 | -0.7917430 | 2.4152460 |
| C | -3.5616070 | -0.4408380 | -1.1622710 |
| H | -1.6240690 | -1.0229290 | -1.8751490 |
| C | -4.3514550 | -0.1891280 | -0.0333130 |
| H | -4.4433370 | -0.1312470 | 2.1253790 |
| H | -3.9861130 | -0.3576330 | -2.1576400 |
| Br | -6.1739340 | 0.3142260 | -0.2561460 |
| I | 5.0804030 | -0.1723020 | -0.1817200 |

[*N*-4Ito-3Clpy]$^+$

*E* = -7897.86946764 a.u.

| Symbol | X | Y | Z |
|---|---|---|---|
| C | 2.4191230 | 0.8847070 | 0.6913780 |
| C | 3.9930770 | -0.6895760 | -0.0761340 |
| C | 4.9817460 | 0.2869750 | -0.1475120 |
| C | 4.6704680 | 1.6041380 | 0.2174560 |
| H | 3.0932530 | 2.9005700 | 0.9310720 |
| H | 1.3953280 | 1.0550460 | 1.0020020 |
| H | 4.1856150 | -1.7229900 | -0.3437700 |
| H | 5.4293310 | 2.3798240 | 0.1664110 |
| C | 1.7216190 | -1.5098220 | 0.4482670 |
| H | 1.9998490 | -2.2241130 | -0.3288090 |
| H | 1.8997510 | -1.9718450 | 1.4225530 |
| N | 2.7416980 | -0.3783980 | 0.3357340 |
| C | 0.2934040 | -1.0651380 | 0.2999630 |
| C | -0.5378090 | -0.9545800 | 1.4249190 |
| C | -0.2374590 | -0.7849700 | -0.9703360 |
| C | -1.8729180 | -0.5626840 | 1.2905080 |
| H | -0.1547820 | -1.1895790 | 2.4162620 |
| C | -1.5666950 | -0.3873740 | -1.1142910 |
| H | 0.3814520 | -0.8852030 | -1.8603140 |
| C | -2.3858030 | -0.2742670 | 0.0194230 |
| H | -2.5047330 | -0.4912900 | 2.1701900 |
| H | -1.9631150 | -0.1791220 | -2.1029440 |
| Cl | 6.5619210 | -0.1487920 | -0.6867300 |
| I | -4.4079740 | 0.3181480 | -0.1980180 |

[*N*-4Brto-3Clpy]$^+$

*E* = -3551.69949856 a.u.

| Symbol | X | Y | Z |
|---|---|---|---|
| C | 2.7863240 | 1.9451420 | 0.4114470 |
| C | 1.8073660 | 0.9648560 | 0.5272800 |
| C | 3.3758160 | -0.7104100 | -0.0001180 |
| C | 4.3883570 | 0.2345330 | -0.1313170 |
| C | 4.0920340 | 1.5889080 | 0.0777870 |
| H | 2.5166200 | 2.9824320 | 0.5807080 |
| H | 0.7761420 | 1.1860540 | 0.7743910 |
| H | 3.5554680 | -1.7700140 | -0.1476590 |
| H | 4.8698350 | 2.3407450 | -0.0221140 |
| C | 1.0688340 | -1.4331750 | 0.5042940 |
| H | 1.3356640 | -2.2047820 | -0.2202500 |
| H | 1.2331730 | -1.8303720 | 1.5090760 |
| N | 2.1155060 | -0.3342650 | 0.3213210 |
| C | -0.3494380 | -0.9729780 | 0.3175360 |
| C | -1.1701450 | -0.7289400 | 1.4298680 |
| C | -0.8825250 | -0.8133740 | -0.9729850 |
| C | -2.4968270 | -0.3230310 | 1.2635170 |
| H | -0.7849090 | -0.8694270 | 2.4380170 |
| C | -2.2038320 | -0.4043380 | -1.1520550 |
| H | -0.2715160 | -1.0187980 | -1.8500500 |
| C | -3.0050140 | -0.1587420 | -0.0292910 |
| H | -3.1278690 | -0.1453860 | 2.1284320 |
| H | -2.6113540 | -0.2893440 | -2.1513570 |
| Br | -4.8118830 | 0.3903590 | -0.2682970 |
| Cl | 5.9794350 | -0.2843830 | -0.5514030 |

[*N*-4Ito-3Brpy]$^+$

$E$ = -10011.39498850 a.u.

| Symbol | X | Y | Z |
|---|---|---|---|
| C | 1.6789060 | 0.9827850 | 0.6493090 |
| C | 3.3127410 | -0.6295950 | 0.1218560 |
| C | 4.2942630 | 0.3501340 | 0.0222320 |
| C | 3.9482530 | 1.6898420 | 0.2467470 |
| H | 2.3178700 | 3.0218260 | 0.7450790 |
| H | 0.6370580 | 1.1655030 | 0.8830400 |
| H | 3.5257350 | -1.6809600 | -0.0382170 |
| H | 4.6980990 | 2.4720310 | 0.1721430 |
| C | 1.0233070 | -1.4380630 | 0.5756820 |
| H | 1.3220750 | -2.1837070 | -0.1635160 |
| H | 1.1905090 | -1.8528450 | 1.5728700 |
| N | 2.0342970 | -0.3018680 | 0.4283490 |
| C | -0.4074780 | -1.0200270 | 0.3848680 |
| C | -1.2477000 | -0.8325680 | 1.4930680 |
| C | -0.9319720 | -0.8408100 | -0.9061480 |
| C | -2.5847980 | -0.4632470 | 1.3213580 |
| H | -0.8693870 | -0.9885760 | 2.5016650 |
| C | -2.2638080 | -0.4674100 | -1.0879480 |
| H | -0.3058090 | -1.0019850 | -1.7819230 |
| C | -3.0913750 | -0.2762470 | 0.0288090 |
| H | -3.2231940 | -0.3312000 | 2.1891620 |
| H | -2.6553530 | -0.3383370 | -2.0919620 |
| I | -5.1171930 | 0.2802330 | -0.2438060 |
| Br | 6.0628010 | -0.1523420 | -0.4157350 |

[*N*-4Ito-3Ipy]$^+$

$E$ = -14357.56648510 a.u.

| Symbol | X | Y | Z |
|---|---|---|---|
| C | 2.0390280 | 1.6983160 | -1.5055230 |
| C | 1.0992570 | 1.4500630 | -0.5152400 |
| C | 2.6514260 | 0.1312900 | 0.6698940 |
| C | 3.6311050 | 0.3421030 | -0.2983400 |
| C | 3.3164860 | 1.1431450 | -1.4057180 |
| H | 1.7654870 | 2.3214760 | -2.3508830 |
| H | 0.0881180 | 1.8388310 | -0.5460400 |
| H | 2.8296090 | -0.4715130 | 1.5535880 |
| H | 4.0543750 | 1.3300540 | -2.1805190 |
| C | 0.4033600 | 0.4616410 | 1.6643430 |
| H | 0.7535610 | -0.4160470 | 2.2106480 |
| H | 0.4886230 | 1.3314730 | 2.3204690 |
| N | 1.4186080 | 0.6777600 | 0.5488510 |
| C | -1.0055320 | 0.2801670 | 1.1667360 |
| C | -1.9576100 | 1.2934350 | 1.3500550 |
| C | -1.3943730 | -0.9151970 | 0.5393820 |
| C | -3.2733610 | 1.1263140 | 0.9073160 |
| H | -1.6855990 | 2.2186730 | 1.8548050 |
| C | -2.7029670 | -1.0897910 | 0.0896930 |
| H | -0.6794490 | -1.7252920 | 0.4053890 |
| C | -3.6439600 | -0.0644580 | 0.2712000 |
| H | -3.9990760 | 1.9178900 | 1.0645400 |
| H | -2.9898910 | -2.0211830 | -0.3883200 |
| I | 5.5296070 | -0.5459600 | -0.0708670 |

(*N*-4Brto-3Brpy)Cl halogen-bonded complex with benzyl fragment

*E* = - 6125.13798437 a.u.

| Symbol | X | Y | Z |
|---|---|---|---|
| Br | 7.1850000 | 6.0892000 | 7.8476000 |
| Br | 3.5913000 | -1.4703000 | 4.8890000 |
| N | 4.5680000 | -0.0353000 | 8.5715000 |
| C | 3.9624000 | -0.2459000 | 7.3947000 |
| H | 3.1980000 | 0.2639000 | 7.1549000 |
| C | 6.1836000 | 4.5741000 | 8.4291000 |
| C | 6.6219000 | 3.8307000 | 9.5110000 |
| H | 7.4002000 | 4.0888000 | 9.9905000 |
| C | 4.4498000 | -1.1990000 | 6.5350000 |
| C | 4.7687000 | 2.3277000 | 9.1801000 |
| C | 5.9070000 | 2.7048000 | 9.8836000 |
| H | 6.1963000 | 2.1862000 | 10.6251000 |
| C | 5.0347000 | 4.2442000 | 7.7419000 |
| H | 4.7374000 | 4.7776000 | 7.0147000 |
| C | 4.0626000 | 1.0392000 | 9.4913000 |
| H | 3.0864000 | 1.1535000 | 9.3700000 |
| H | 4.2303000 | 0.7826000 | 10.4325000 |
| C | 4.3229000 | 3.1209000 | 8.1272000 |
| H | 3.5215000 | 2.8919000 | 7.6711000 |
| C | 5.6670000 | -0.7317000 | 8.9244000 |
| H | 6.0873000 | -0.5576000 | 9.7580000 |
| C | 5.5681000 | -1.9441000 | 6.8787000 |
| H | 5.9030000 | -2.6122000 | 6.2920000 |
| C | 6.1845000 | -1.6948000 | 8.0878000 |
| H | 6.9589000 | -2.1839000 | 8.3408000 |
| Cl | 8.3186000 | 8.7746000 | 6.4869000 |

(*N*-4Brto-3Clpy)I halogen-bonded complex with pyridyl fragment

*E* = - 10471.73564560 a.u.

| Symbol | X | Y | Z |
|---|---|---|---|
| Br | 5.1798000 | 6.7598000 | 8.2923000 |
| Cl | 0.1188000 | 6.5104000 | -0.1251000 |
| N | 1.6030000 | 4.3681000 | 2.8229000 |
| C | 0.8522000 | 5.2360000 | 2.1195000 |
| H | 0.1426000 | 5.7049000 | 2.5419000 |
| C | 2.3061000 | 4.7609000 | 5.1867000 |
| C | 4.0213000 | 5.9226000 | 7.0339000 |
| C | 3.0885000 | 4.9951000 | 7.4664000 |
| H | 3.0333000 | 4.7583000 | 8.3844000 |
| C | 3.2529000 | 5.6943000 | 4.7769000 |
| H | 3.3086000 | 5.9398000 | 3.8607000 |
| C | 1.1187000 | 5.4383000 | 0.7875000 |
| C | 2.2327000 | 4.4151000 | 6.5361000 |
| H | 1.5913000 | 3.7762000 | 6.8227000 |
| C | 1.3083000 | 4.1213000 | 4.2550000 |
| H | 1.2977000 | 3.1448000 | 4.4177000 |
| H | 0.4054000 | 4.4712000 | 4.4621000 |
| C | 2.6239000 | 3.6966000 | 2.2490000 |
| H | 3.1369000 | 3.0832000 | 2.7618000 |
| C | 2.1680000 | 4.7802000 | 0.1735000 |
| H | 2.3664000 | 4.9301000 | -0.7435000 |
| C | 2.9227000 | 3.9006000 | 0.9236000 |
| H | 3.6476000 | 3.4352000 | 0.5233000 |
| C | 4.1151000 | 6.2667000 | 5.6979000 |
| H | 4.7684000 | 6.8946000 | 5.4125000 |
| I | -1.4247000 | 8.0491000 | -3.1718000 |

(*N*-4Ito-3Clpy)Br halogen-bonded complex with pyridyl fragment

*E* = -10471.72014600 a.u.

| Symbol | X | Y | Z |
|---|---|---|---|
| I | 4.5226000 | 6.7852000 | 4.7802000 |
| Cl | 9.5711000 | 6.5354000 | 13.2043000 |
| N | 8.2032000 | 4.2235000 | 10.3421000 |
| C | 5.7939000 | 5.8065000 | 6.1501000 |
| C | 6.5138000 | 5.4784000 | 8.4062000 |
| H | 6.4236000 | 5.6704000 | 9.3319000 |
| C | 7.1890000 | 3.5440000 | 10.9345000 |
| H | 6.7057000 | 2.8891000 | 10.4442000 |
| C | 5.6522000 | 6.0655000 | 7.4949000 |
| H | 4.9645000 | 6.6467000 | 7.7980000 |
| C | 6.7860000 | 4.9388000 | 5.6918000 |
| H | 6.8850000 | 4.7615000 | 4.7634000 |
| C | 7.5654000 | 4.7439000 | 12.9563000 |
| H | 7.3421000 | 4.9401000 | 13.8590000 |
| C | 7.6253000 | 4.3409000 | 6.6200000 |
| H | 8.2915000 | 3.7340000 | 6.3188000 |
| C | 8.4926000 | 3.9371000 | 8.9126000 |
| H | 8.4628000 | 2.9585000 | 8.7664000 |
| H | 9.4087000 | 4.2470000 | 8.6997000 |
| C | 8.9113000 | 5.1453000 | 11.0061000 |
| H | 9.6101000 | 5.6183000 | 10.5704000 |
| C | 7.5171000 | 4.6049000 | 7.9727000 |
| C | 6.8602000 | 3.8055000 | 12.2428000 |
| H | 6.1444000 | 3.3358000 | 12.6540000 |
| C | 8.6175000 | 5.3993000 | 12.3210000 |
| Br | 11.0479000 | 8.0204000 | 16.1443000 |

(*N*-4Ito-3Clpy)I halogen-bonded complex with pyridyl fragment

*E* = -14818.29185610 a.u.

| Symbol | X | Y | Z |
|---|---|---|---|
| I | 4.5420000 | 6.8394000 | 5.0403000 |
| Cl | 9.7928000 | 6.5880000 | 13.5746000 |
| N | 8.3159000 | 4.4324000 | 10.6317000 |
| C | 5.8506000 | 5.9273000 | 6.4217000 |
| C | 7.6018000 | 4.8104000 | 8.2708000 |
| C | 6.8226000 | 5.0451000 | 5.9920000 |
| C | 9.0683000 | 5.3053000 | 11.3335000 |
| C | 6.6074000 | 5.6909000 | 8.6840000 |
| C | 7.6959000 | 4.4892000 | 6.9189000 |
| C | 8.7912000 | 5.5163000 | 12.6685000 |
| C | 5.7256000 | 6.2460000 | 7.7638000 |
| C | 8.6071000 | 4.1824000 | 9.2073000 |
| C | 6.9804000 | 3.9832000 | 12.5280000 |
| C | 7.2872000 | 3.7722000 | 11.2116000 |
| C | 7.7374000 | 4.8773000 | 13.2805000 |
| H | 5.0418000 | 6.8384000 | 8.0534000 |
| H | 6.5309000 | 5.9155000 | 9.6039000 |
| H | 8.3667000 | 3.8824000 | 6.6268000 |
| H | 6.8930000 | 4.8205000 | 5.0717000 |
| H | 9.7820000 | 5.7697000 | 10.9126000 |
| H | 6.7751000 | 3.1569000 | 10.7011000 |
| H | 6.2534000 | 3.5213000 | 12.9274000 |
| H | 7.5317000 | 5.0417000 | 14.1932000 |
| H | 9.5065000 | 4.5366000 | 8.9966000 |
| H | 8.6230000 | 3.2044000 | 9.0507000 |
| I | 11.3565000 | 8.1160000 | 16.6416000 |

(*N*-4Ito-3Clpy)Br halogen-bonded complex with benzoyl fragment

*E* = -10471.58460360 a.u.

| Symbol | X | Y | Z |
|---|---|---|---|
| I | 4.5226000 | 6.7852000 | 4.7802000 |
| Cl | 9.5711000 | 6.5354000 | 13.2043000 |
| N | 8.2032000 | 4.2235000 | 10.3421000 |
| C | 5.7939000 | 5.8065000 | 6.1501000 |
| C | 6.5138000 | 5.4784000 | 8.4062000 |
| H | 6.4236000 | 5.6704000 | 9.3319000 |
| C | 7.1890000 | 3.5440000 | 10.9345000 |
| H | 6.7057000 | 2.8891000 | 10.4442000 |
| C | 5.6522000 | 6.0655000 | 7.4949000 |
| H | 4.9645000 | 6.6467000 | 7.7980000 |
| C | 6.7860000 | 4.9388000 | 5.6918000 |
| H | 6.8850000 | 4.7615000 | 4.7634000 |
| C | 7.5654000 | 4.7439000 | 12.9563000 |
| H | 7.3421000 | 4.9401000 | 13.8590000 |
| C | 7.6253000 | 4.3409000 | 6.6200000 |
| H | 8.2915000 | 3.7340000 | 6.3188000 |
| C | 8.4926000 | 3.9371000 | 8.9126000 |
| H | 8.4628000 | 2.9585000 | 8.7664000 |
| H | 9.4087000 | 4.2470000 | 8.6997000 |
| C | 8.9113000 | 5.1453000 | 11.0061000 |
| H | 9.6101000 | 5.6183000 | 10.5704000 |
| C | 7.5171000 | 4.6049000 | 7.9727000 |
| C | 6.8602000 | 3.8055000 | 12.2428000 |
| H | 6.1444000 | 3.3358000 | 12.6540000 |
| C | 8.6175000 | 5.3993000 | 12.3210000 |
| Br | 1.3867000 | 8.0204000 | 3.1255000 |

(*N*-4Ito-3Clpy)I halogen-bonded complex with benzoyl fragment

*E* = -14818.28036720 a.u.

| Symbol | X | Y | Z |
|---|---|---|---|
| I | 4.5420000 | 6.8394000 | 5.0403000 |
| Cl | 9.7928000 | 6.5880000 | 13.5746000 |
| N | 8.3159000 | 4.4324000 | 10.6317000 |
| C | 5.8506000 | 5.9273000 | 6.4217000 |
| C | 7.6018000 | 4.8104000 | 8.2708000 |
| C | 6.8226000 | 5.0451000 | 5.9920000 |
| C | 9.0683000 | 5.3053000 | 11.3335000 |
| C | 6.6074000 | 5.6909000 | 8.6840000 |
| C | 7.6959000 | 4.4892000 | 6.9189000 |
| C | 8.7912000 | 5.5163000 | 12.6685000 |
| C | 5.7256000 | 6.2460000 | 7.7638000 |
| C | 8.6071000 | 4.1824000 | 9.2073000 |
| C | 6.9804000 | 3.9832000 | 12.5280000 |
| C | 7.2872000 | 3.7722000 | 11.2116000 |
| C | 7.7374000 | 4.8773000 | 13.2805000 |
| H | 5.0418000 | 6.8384000 | 8.0534000 |
| H | 6.5309000 | 5.9155000 | 9.6039000 |
| H | 8.3667000 | 3.8824000 | 6.6268000 |
| H | 6.8930000 | 4.8205000 | 5.0717000 |
| H | 9.7820000 | 5.7697000 | 10.9126000 |
| H | 6.7751000 | 3.1569000 | 10.7011000 |
| H | 6.2534000 | 3.5213000 | 12.9274000 |
| H | 7.5317000 | 5.0417000 | 14.1932000 |
| H | 9.5065000 | 4.5366000 | 8.9966000 |
| H | 8.6230000 | 3.2044000 | 9.0507000 |
| I | 1.4750000 | 8.1160000 | 3.2026000 |

(*N*-4Brto-3Ipy)Cl halogen-bonded complex with benzoyl fragment

*E* = -10471.69057290 a.u.

| Symbol | X | Y | Z |
|---|---|---|---|
| I | 14.2085000 | 1.8931000 | 4.9025000 |
| Br | 24.4912000 | 4.7188000 | 3.5253000 |
| N | 17.6973000 | 3.8283000 | 3.2327000 |
| C | 15.9552000 | 2.3144000 | 3.8012000 |
| C | 22.5990000 | 4.9183000 | 3.4863000 |
| C | 16.6248000 | 3.5081000 | 3.9991000 |
| H | 16.3318000 | 4.1074000 | 4.6753000 |
| C | 21.9909000 | 5.3543000 | 2.3395000 |
| H | 22.5064000 | 5.5888000 | 1.5765000 |
| C | 19.8358000 | 5.0985000 | 3.4350000 |
| C | 21.8628000 | 4.5938000 | 4.6232000 |
| H | 22.3046000 | 4.3106000 | 5.4154000 |
| C | 20.5958000 | 5.4465000 | 2.3141000 |
| H | 20.1595000 | 5.7486000 | 1.5262000 |
| C | 17.5131000 | 1.8005000 | 2.0419000 |
| H | 17.8251000 | 1.2137000 | 1.3631000 |
| C | 20.4775000 | 4.6875000 | 4.5898000 |
| H | 19.9691000 | 4.4680000 | 5.3616000 |
| C | 16.4231000 | 1.4503000 | 2.8257000 |
| H | 15.9953000 | 0.6128000 | 2.6921000 |
| C | 18.3256000 | 5.1821000 | 3.4046000 |
| H | 18.0038000 | 5.5869000 | 4.2492000 |
| H | 18.0453000 | 5.7697000 | 2.6590000 |
| C | 18.1376000 | 3.0099000 | 2.2615000 |
| H | 18.8793000 | 3.2654000 | 1.7254000 |
| Cl | 27.2087000 | 2.4372000 | 3.0143000 |

(*N*-4Brto-3Ipy)I halogen-bonded complex with benzoyl fragment

*E* = -16931.65939460 a.u.

| Symbol | X | Y | Z |
|---|---|---|---|
| I | 4.5858000 | 1.9837000 | 7.1239000 |
| Br | -0.2833000 | 1.6498000 | -1.6045000 |
| N | 3.0299000 | 4.3509000 | 3.8616000 |
| C | 3.4165000 | 3.2319000 | 5.9048000 |
| C | 3.7526000 | 3.4795000 | 4.5946000 |
| H | 4.4955000 | 3.0348000 | 4.2046000 |
| C | 1.6575000 | 3.5845000 | -0.7827000 |
| H | 1.6916000 | 3.8228000 | -1.7023000 |
| C | 0.8104000 | 2.5748000 | -0.3366000 |
| C | 2.3181000 | 3.8784000 | 6.4631000 |
| H | 2.0668000 | 3.7129000 | 7.3640000 |
| C | 1.5986000 | 4.7630000 | 5.6885000 |
| H | 0.8457000 | 5.2095000 | 6.0564000 |
| C | 2.4173000 | 3.8784000 | 1.4879000 |
| C | 2.4514000 | 4.2328000 | 0.1483000 |
| H | 3.0289000 | 4.9318000 | -0.1352000 |
| C | 3.3593000 | 4.6037000 | 2.4429000 |
| H | 4.2883000 | 4.3095000 | 2.2682000 |
| H | 3.3118000 | 5.5773000 | 2.2678000 |
| C | 0.7590000 | 2.2091000 | 0.9870000 |
| H | 0.1745000 | 1.5161000 | 1.2714000 |
| C | 1.5677000 | 2.8592000 | 1.9033000 |
| H | 1.5423000 | 2.6088000 | 2.8199000 |
| C | 1.9660000 | 5.0001000 | 4.3902000 |
| H | 1.4747000 | 5.6190000 | 3.8626000 |
| I | -3.2992000 | 0.2859000 | -3.5538000 |

(*N*-4Brto-3Ipy)Br halogen-bonded complex with pyridyl fragment

*E* = -12585.31172930 a.u.

| Symbol | X | Y | Z |
|---|---|---|---|
| I | 4.5795000 | 1.9112000 | 6.9973000 |
| Br | -0.1900000 | 1.5846000 | -1.6781000 |
| N | 3.0309000 | 4.3564000 | 3.7970000 |
| C | 3.4267000 | 3.1920000 | 5.8034000 |
| C | 2.3426000 | 3.8486000 | 6.4007000 |
| H | 2.1010000 | 3.6719000 | 7.3021000 |
| C | 1.7309000 | 3.5319000 | -0.8558000 |
| H | 1.7894000 | 3.7442000 | -1.7797000 |
| C | 1.9882000 | 5.0182000 | 4.3477000 |
| H | 1.5067000 | 5.6593000 | 3.8390000 |
| C | 3.7496000 | 3.4519000 | 4.4938000 |
| H | 4.4750000 | 2.9975000 | 4.0815000 |
| C | 2.5108000 | 4.2067000 | 0.0853000 |
| H | 3.0954000 | 4.8983000 | -0.2030000 |
| C | 3.3496000 | 4.6284000 | 2.3800000 |
| H | 4.2885000 | 4.3682000 | 2.2048000 |
| H | 3.2686000 | 5.6007000 | 2.2123000 |
| C | 2.4470000 | 3.8874000 | 1.4234000 |
| C | 1.6327000 | 4.7609000 | 5.6405000 |
| H | 0.8897000 | 5.2128000 | 6.0230000 |
| C | 1.5766000 | 2.8882000 | 1.8461000 |
| H | 1.5193000 | 2.6652000 | 2.7680000 |
| C | 0.8736000 | 2.5483000 | -0.4085000 |
| C | 0.7905000 | 2.2161000 | 0.9179000 |
| H | 0.1984000 | 1.5293000 | 1.2019000 |
| Br | 6.1119000 | 0.3031000 | 9.4611000 |

(*N*-4Brto-3Ipy)Cl halogen-bonded complex with pyridyl fragment

*E* = -10471.74034470 a.u.

| Symbol | X | Y | Z |
|---|---|---|---|
| N | 17.6973000 | 3.8283000 | 3.2327000 |
| C | 15.9552000 | 2.3144000 | 3.8012000 |
| C | 22.5990000 | 4.9183000 | 3.4863000 |
| C | 16.6248000 | 3.5081000 | 3.9991000 |
| H | 16.3318000 | 4.1074000 | 4.6753000 |
| C | 21.9909000 | 5.3543000 | 2.3395000 |
| H | 22.5064000 | 5.5888000 | 1.5765000 |
| C | 19.8358000 | 5.0985000 | 3.4350000 |
| C | 21.8628000 | 4.5938000 | 4.6232000 |
| H | 22.3046000 | 4.3106000 | 5.4154000 |
| C | 20.5958000 | 5.4465000 | 2.3141000 |
| H | 20.1595000 | 5.7486000 | 1.5262000 |
| C | 17.5131000 | 1.8005000 | 2.0419000 |
| H | 17.8251000 | 1.2137000 | 1.3631000 |
| C | 20.4775000 | 4.6875000 | 4.5898000 |
| H | 19.9691000 | 4.4680000 | 5.3616000 |
| C | 16.4231000 | 1.4503000 | 2.8257000 |
| H | 15.9953000 | 0.6128000 | 2.6921000 |
| C | 18.3256000 | 5.1821000 | 3.4046000 |
| H | 18.0038000 | 5.5869000 | 4.2492000 |
| H | 18.0453000 | 5.7697000 | 2.6590000 |
| C | 18.1376000 | 3.0099000 | 2.2615000 |
| H | 18.8793000 | 3.2654000 | 1.7254000 |
| Cl | 11.3568000 | 1.1366000 | 6.1072000 |

(*N*-4Brto-3Ipy)I halogen-bonded complex with pyridyl fragment

*E* = -16931.87931610 a.u.

| Symbol | X | Y | Z |
|---|---|---|---|
| I | 6.1417000 | 0.2859000 | 9.8025000 |
| I | 4.5858000 | 1.9837000 | 7.1239000 |
| Br | -0.2833000 | 1.6498000 | -1.6045000 |
| N | 3.0299000 | 4.3509000 | 3.8616000 |
| C | 3.4165000 | 3.2319000 | 5.9048000 |
| C | 3.7526000 | 3.4795000 | 4.5946000 |
| H | 4.4955000 | 3.0348000 | 4.2046000 |
| C | 1.6575000 | 3.5845000 | -0.7827000 |
| H | 1.6916000 | 3.8228000 | -1.7023000 |
| C | 0.8104000 | 2.5748000 | -0.3366000 |
| C | 2.3181000 | 3.8784000 | 6.4631000 |
| H | 2.0668000 | 3.7129000 | 7.3640000 |
| C | 1.5986000 | 4.7630000 | 5.6885000 |
| H | 0.8457000 | 5.2095000 | 6.0564000 |
| C | 2.4173000 | 3.8784000 | 1.4879000 |
| C | 2.4514000 | 4.2328000 | 0.1483000 |
| H | 3.0289000 | 4.9318000 | -0.1352000 |
| C | 3.3593000 | 4.6037000 | 2.4429000 |
| H | 4.2883000 | 4.3095000 | 2.2682000 |
| H | 3.3118000 | 5.5773000 | 2.2678000 |
| C | 0.7590000 | 2.2091000 | 0.9870000 |
| H | 0.1745000 | 1.5161000 | 1.2714000 |
| C | 1.5677000 | 2.8592000 | 1.9033000 |
| H | 1.5423000 | 2.6088000 | 2.8199000 |
| C | 1.9660000 | 5.0001000 | 4.3902000 |
| H | 1.4747000 | 5.6190000 | 3.8626000 |

(*N*-4Ito-3Ipy)Br halogen-bonded complex with benzoyl fragment

*E* = -16931.83529440 a.u.

| Symbol | X | Y | Z |
|---|---|---|---|
| I | -0.0275000 | 2.0829000 | 14.9982000 |
| I | 4.2677000 | 4.3528000 | 5.1286000 |
| N | 2.6001000 | 3.6614000 | 11.9864000 |
| C | 1.6116000 | 3.4745000 | 12.8701000 |
| H | 0.9625000 | 4.1568000 | 12.9943000 |
| C | 1.5176000 | 2.3148000 | 13.6014000 |
| C | 2.7158000 | 4.9856000 | 11.2847000 |
| H | 1.8526000 | 5.4652000 | 11.3549000 |
| H | 3.4039000 | 5.5334000 | 11.7400000 |
| C | 3.5385000 | 2.7071000 | 11.7779000 |
| H | 4.2354000 | 2.8557000 | 11.1494000 |
| C | 4.6577000 | 5.1454000 | 8.0407000 |
| H | 5.5131000 | 5.4266000 | 7.7373000 |
| C | 3.4842000 | 1.5249000 | 12.4730000 |
| H | 4.1378000 | 0.8519000 | 12.3262000 |
| C | 2.4619000 | 1.3226000 | 13.3956000 |
| H | 2.4123000 | 0.5078000 | 13.8816000 |
| C | 4.3286000 | 5.2466000 | 9.3865000 |
| H | 4.9633000 | 5.5929000 | 10.0024000 |
| C | 3.0810000 | 4.8458000 | 9.8385000 |
| C | 3.7457000 | 4.6381000 | 7.1518000 |
| C | 2.1578000 | 4.3308000 | 8.9087000 |
| H | 1.2994000 | 4.0513000 | 9.2034000 |
| C | 2.4873000 | 4.2297000 | 7.5800000 |
| H | 1.8600000 | 3.8819000 | 6.9571000 |
| Br | 5.2337000 | 2.5644000 | 2.2341000 |

(*N*-4Ito-3Ipy)Cl halogen-bonded complex with benzoyl fragment

*E* = -14818.25586440 a.u.

| Symbol | X | Y | Z |
|---|---|---|---|
| I | 0.6805000 | 5.4235000 | 15.0403000 |
| I | 4.5939000 | 2.8936000 | 5.1042000 |
| N | 3.1729000 | 3.6685000 | 11.9937000 |
| C | 2.1613000 | 5.0923000 | 13.5825000 |
| C | 4.1351000 | 2.6358000 | 7.1337000 |
| C | 2.1893000 | 3.9075000 | 12.8865000 |
| C | 4.1486000 | 4.5663000 | 11.7788000 |
| C | 5.0968000 | 2.1762000 | 8.0165000 |
| C | 4.7925000 | 2.0906000 | 9.3741000 |
| C | 4.1563000 | 5.7680000 | 12.4540000 |
| C | 3.5428000 | 2.4454000 | 9.8470000 |
| C | 2.8622000 | 2.9847000 | 7.5930000 |
| C | 3.1455000 | 6.0395000 | 13.3583000 |
| C | 3.2294000 | 2.3252000 | 11.3186000 |
| C | 2.5745000 | 2.8822000 | 8.9413000 |
| H | 1.7075000 | 3.1116000 | 9.2551000 |
| H | 2.1999000 | 3.2901000 | 6.9839000 |
| H | 5.9551000 | 1.9209000 | 7.7003000 |
| H | 5.4541000 | 1.7845000 | 9.9833000 |
| H | 1.5150000 | 3.2554000 | 13.0339000 |
| H | 4.8396000 | 4.3716000 | 11.1564000 |
| H | 4.8471000 | 6.4017000 | 12.3003000 |
| H | 3.1273000 | 6.8686000 | 13.8215000 |
| H | 3.9236000 | 1.7705000 | 11.7550000 |
| H | 2.3586000 | 1.8671000 | 11.4304000 |
| Cl | 5.5608000 | 4.8837000 | 2.3921000 |

(*N*-4Ito-3Ipy)I halogen-bonded complex with benzoyl fragment

*E* = -21277.49045570 a.u.

| Symbol | X | Y | Z |
|---|---|---|---|
| I | 4.9584000 | 6.8123000 | 6.2629000 |
| I | 10.0797000 | 7.1548000 | 15.1430000 |
| N | 6.5400000 | 4.4843000 | 9.5375000 |
| C | 6.1411000 | 5.5904000 | 7.4851000 |
| C | 5.8066000 | 5.3420000 | 8.7995000 |
| H | 5.0559000 | 5.7772000 | 9.1869000 |
| C | 7.1691000 | 4.9220000 | 11.9063000 |
| C | 7.2655000 | 4.9660000 | 6.9275000 |
| H | 7.5224000 | 5.1385000 | 6.0298000 |
| C | 8.8352000 | 6.1742000 | 13.7527000 |
| C | 8.0765000 | 5.8924000 | 11.4875000 |
| H | 8.1283000 | 6.1337000 | 10.5698000 |
| C | 6.2071000 | 4.2245000 | 10.9556000 |
| H | 6.2348000 | 3.2490000 | 11.1232000 |
| H | 5.2863000 | 4.5384000 | 11.1372000 |
| C | 7.9311000 | 5.2099000 | 14.1863000 |
| H | 7.8798000 | 4.9761000 | 15.1054000 |
| C | 7.9896000 | 4.0933000 | 7.7153000 |
| H | 8.7527000 | 3.6570000 | 7.3545000 |
| C | 7.1050000 | 4.5926000 | 13.2557000 |
| H | 6.4845000 | 3.9346000 | 13.5472000 |
| C | 8.9125000 | 6.5089000 | 12.4248000 |
| H | 9.5373000 | 7.1643000 | 12.1404000 |
| C | 7.6211000 | 3.8484000 | 9.0149000 |
| H | 8.1191000 | 3.2386000 | 9.5465000 |
| I | 13.1239000 | 8.4911000 | 17.0414000 |

(*N*-4Ito-3Ipy)Br halogen-bonded complex with pyridyl fragment

*E* = -16931.87125520 a.u.

| Symbol | X | Y | Z |
|---|---|---|---|
| I | -0.0275000 | 2.0829000 | 14.9982000 |
| I | 4.2677000 | 4.3528000 | 5.1286000 |
| N | 2.6001000 | 3.6614000 | 11.9864000 |
| C | 1.6116000 | 3.4745000 | 12.8701000 |
| H | 0.9625000 | 4.1568000 | 12.9943000 |
| C | 1.5176000 | 2.3148000 | 13.6014000 |
| C | 2.7158000 | 4.9856000 | 11.2847000 |
| H | 1.8526000 | 5.4652000 | 11.3549000 |
| H | 3.4039000 | 5.5334000 | 11.7400000 |
| C | 3.5385000 | 2.7071000 | 11.7779000 |
| H | 4.2354000 | 2.8557000 | 11.1494000 |
| C | 4.6577000 | 5.1454000 | 8.0407000 |
| H | 5.5131000 | 5.4266000 | 7.7373000 |
| C | 3.4842000 | 1.5249000 | 12.4730000 |
| H | 4.1378000 | 0.8519000 | 12.3262000 |
| C | 2.4619000 | 1.3226000 | 13.3956000 |
| H | 2.4123000 | 0.5078000 | 13.8816000 |
| C | 4.3286000 | 5.2466000 | 9.3865000 |
| H | 4.9633000 | 5.5929000 | 10.0024000 |
| C | 3.0810000 | 4.8458000 | 9.8385000 |
| C | 3.7457000 | 4.6381000 | 7.1518000 |
| C | 2.1578000 | 4.3308000 | 8.9087000 |
| H | 1.2994000 | 4.0513000 | 9.2034000 |
| C | 2.4873000 | 4.2297000 | 7.5800000 |
| H | 1.8600000 | 3.8819000 | 6.9571000 |
| Br | -2.2912000 | 1.2962000 | 17.2989000 |

(*N*-4Ito-3Ipy)Cl halogen-bonded complex with pyridyl fragment

*E* = -14818.29697400 a.u.

| Symbol | X | Y | Z |
|---|---|---|---|
| I | 0.6805000 | 5.4235000 | 15.0403000 |
| I | 4.5939000 | 2.8936000 | 5.1042000 |
| N | 3.1729000 | 3.6685000 | 11.9937000 |
| C | 2.1613000 | 5.0923000 | 13.5825000 |
| C | 4.1351000 | 2.6358000 | 7.1337000 |
| C | 2.1893000 | 3.9075000 | 12.8865000 |
| C | 4.1486000 | 4.5663000 | 11.7788000 |
| C | 5.0968000 | 2.1762000 | 8.0165000 |
| C | 4.7925000 | 2.0906000 | 9.3741000 |
| C | 4.1563000 | 5.7680000 | 12.4540000 |
| C | 3.5428000 | 2.4454000 | 9.8470000 |
| C | 2.8622000 | 2.9847000 | 7.5930000 |
| C | 3.1455000 | 6.0395000 | 13.3583000 |
| C | 3.2294000 | 2.3252000 | 11.3186000 |
| C | 2.5745000 | 2.8822000 | 8.9413000 |
| H | 1.7075000 | 3.1116000 | 9.2551000 |
| H | 2.1999000 | 3.2901000 | 6.9839000 |
| H | 5.9551000 | 1.9209000 | 7.7003000 |
| H | 5.4541000 | 1.7845000 | 9.9833000 |
| H | 1.5150000 | 3.2554000 | 13.0339000 |
| H | 4.8396000 | 4.3716000 | 11.1564000 |
| H | 4.8471000 | 6.4017000 | 12.3003000 |
| H | 3.1273000 | 6.8686000 | 13.8215000 |
| H | 3.9236000 | 1.7705000 | 11.7550000 |
| H | 2.3586000 | 1.8671000 | 11.4304000 |
| Cl | -1.2304000 | 6.1817000 | 17.4630000 |

(*N*-4Ito-3Ipy)I halogen-bonded complex with pyridyl fragment

*E* = -21278.43556160 a.u.

| Symbol | X | Y | Z |
|---|---|---|---|
| I | 4.9584000 | 6.8123000 | 6.2629000 |
| I | 10.0797000 | 7.1548000 | 15.1430000 |
| N | 6.5400000 | 4.4843000 | 9.5375000 |
| C | 6.1411000 | 5.5904000 | 7.4851000 |
| C | 5.8066000 | 5.3420000 | 8.7995000 |
| H | 5.0559000 | 5.7772000 | 9.1869000 |
| C | 7.1691000 | 4.9220000 | 11.9063000 |
| C | 7.2655000 | 4.9660000 | 6.9275000 |
| H | 7.5224000 | 5.1385000 | 6.0298000 |
| C | 8.8352000 | 6.1742000 | 13.7527000 |
| C | 8.0765000 | 5.8924000 | 11.4875000 |
| H | 8.1283000 | 6.1337000 | 10.5698000 |
| C | 6.2071000 | 4.2245000 | 10.9556000 |
| H | 6.2348000 | 3.2490000 | 11.1232000 |
| H | 5.2863000 | 4.5384000 | 11.1372000 |
| C | 7.9311000 | 5.2099000 | 14.1863000 |
| H | 7.8798000 | 4.9761000 | 15.1054000 |
| C | 7.9896000 | 4.0933000 | 7.7153000 |
| H | 8.7527000 | 3.6570000 | 7.3545000 |
| C | 7.1050000 | 4.5926000 | 13.2557000 |
| H | 6.4845000 | 3.9346000 | 13.5472000 |
| C | 8.9125000 | 6.5089000 | 12.4248000 |
| H | 9.5373000 | 7.1643000 | 12.1404000 |
| C | 7.6211000 | 3.8484000 | 9.0149000 |
| H | 8.1191000 | 3.2386000 | 9.5465000 |
| I | 3.3840000 | 8.4911000 | 3.5742000 |

(*N*-4Brto-3Brpy)I halogen-bonded complex with benzoyl fragment

*E* = -12585.28082150 a.u.

| Symbol | X | Y | Z |
|---|---|---|---|
| Br | 4.6445000 | 6.5674000 | 6.9652000 |
| Br | -0.3304000 | 6.8556000 | -1.6104000 |
| N | 3.1395000 | 4.3488000 | 3.8704000 |
| C | 0.8062000 | 5.9800000 | -0.3444000 |
| C | 2.0910000 | 3.6944000 | 4.4097000 |
| H | 1.5913000 | 3.0822000 | 3.8824000 |
| C | 1.7128000 | 5.0413000 | -0.7783000 |
| H | 1.7676000 | 4.8107000 | -1.6983000 |
| C | 3.5688000 | 5.4258000 | 5.9144000 |
| C | 2.4956000 | 4.7901000 | 6.4964000 |
| H | 2.2713000 | 4.9463000 | 7.4061000 |
| C | 2.4737000 | 4.7740000 | 1.4966000 |
| C | 3.8813000 | 5.2119000 | 4.5940000 |
| H | 4.6132000 | 5.6672000 | 4.1952000 |
| C | 0.7175000 | 6.3331000 | 0.9786000 |
| H | 0.0864000 | 6.9866000 | 1.2589000 |
| C | 1.5568000 | 5.7262000 | 1.9078000 |
| H | 1.5011000 | 5.9661000 | 2.8250000 |
| C | 3.4540000 | 4.1061000 | 2.4418000 |
| H | 3.4508000 | 3.1310000 | 2.2723000 |
| H | 4.3649000 | 4.4434000 | 2.2508000 |
| C | 2.5464000 | 4.4379000 | 0.1495000 |
| H | 3.1748000 | 3.7870000 | -0.1387000 |
| C | 1.7461000 | 3.9117000 | 5.7168000 |
| H | 0.9973000 | 3.4638000 | 6.0921000 |
| I | -3.3538000 | 8.1787000 | -3.5285000 |

(*N*-4Brto-3Brpy)Br halogen-bonded complex with pyridyl fragment

*E* = -8238.73873758 a.u.

| Symbol | X | Y | Z |
|---|---|---|---|
| Br | 6.0393000 | 8.0449000 | 9.5205000 |
| Br | 4.5825000 | 6.4817000 | 6.8318000 |
| Br | -0.2333000 | 6.7640000 | -1.6715000 |
| N | 3.1489000 | 4.1452000 | 3.7922000 |
| C | 3.8620000 | 5.0470000 | 4.4910000 |
| H | 4.5834000 | 5.5095000 | 4.0799000 |
| C | 2.4781000 | 4.6447000 | 6.4044000 |
| H | 2.2452000 | 4.8217000 | 7.3087000 |
| C | 3.5369000 | 5.2956000 | 5.8089000 |
| C | 2.1075000 | 3.4777000 | 4.3460000 |
| H | 1.6195000 | 2.8421000 | 3.8359000 |
| C | 3.4660000 | 3.8742000 | 2.3665000 |
| H | 3.4216000 | 2.8985000 | 2.2035000 |
| H | 4.3900000 | 4.1728000 | 2.1764000 |
| C | 1.8018000 | 4.9174000 | -0.8570000 |
| H | 1.8806000 | 4.7198000 | -1.7830000 |
| C | 1.7618000 | 3.7279000 | 5.6535000 |
| H | 1.0268000 | 3.2689000 | 6.0446000 |
| C | 0.8732000 | 5.8400000 | -0.4143000 |
| C | 2.6134000 | 4.2865000 | 0.0673000 |
| H | 3.2500000 | 3.6461000 | -0.2288000 |
| C | 1.5784000 | 5.5150000 | 1.8474000 |
| H | 1.5078000 | 5.7303000 | 2.7697000 |
| C | 0.7439000 | 6.1417000 | 0.9243000 |
| H | 0.0939000 | 6.7706000 | 1.2145000 |
| C | 2.5123000 | 4.5782000 | 1.4267000 |

(*N*-4Brto-3Brpy)I halogen-bonded complex with pyridyl fragment

*E* = -12585.30704250 a.u.

| Symbol | X | Y | Z |
|---|---|---|---|
| I | 6.1723000 | 8.1787000 | 9.8222000 |
| Br | 4.6445000 | 6.5674000 | 6.9652000 |
| Br | -0.3304000 | 6.8556000 | -1.6104000 |
| N | 3.1395000 | 4.3488000 | 3.8704000 |
| C | 0.8062000 | 5.9800000 | -0.3444000 |
| C | 2.0910000 | 3.6944000 | 4.4097000 |
| H | 1.5913000 | 3.0822000 | 3.8824000 |
| C | 1.7128000 | 5.0413000 | -0.7783000 |
| H | 1.7676000 | 4.8107000 | -1.6983000 |
| C | 3.5688000 | 5.4258000 | 5.9144000 |
| C | 2.4956000 | 4.7901000 | 6.4964000 |
| H | 2.2713000 | 4.9463000 | 7.4061000 |
| C | 2.4737000 | 4.7740000 | 1.4966000 |
| C | 3.8813000 | 5.2119000 | 4.5940000 |
| H | 4.6132000 | 5.6672000 | 4.1952000 |
| C | 0.7175000 | 6.3331000 | 0.9786000 |
| H | 0.0864000 | 6.9866000 | 1.2589000 |
| C | 1.5568000 | 5.7262000 | 1.9078000 |
| H | 1.5011000 | 5.9661000 | 2.8250000 |
| C | 3.4540000 | 4.1061000 | 2.4418000 |
| H | 3.4508000 | 3.1310000 | 2.2723000 |
| H | 4.3649000 | 4.4434000 | 2.2508000 |
| C | 2.5464000 | 4.4379000 | 0.1495000 |
| H | 3.1748000 | 3.7870000 | -0.1387000 |
| C | 1.7461000 | 3.9117000 | 5.7168000 |
| H | 0.9973000 | 3.4638000 | 6.0921000 |

(*N*-4Ito-3Brpy)Br halogen-bonded complex with pyridyl fragment

*E* = -12585.29404750 a.u.

| Symbol | X | Y | Z |
|---|---|---|---|
| I | -0.2734000 | 1.5581000 | -1.7324000 |
| Br | 4.7500000 | 1.8527000 | 6.8895000 |
| N | 3.3147000 | 4.1848000 | 3.8469000 |
| C | 0.9812000 | 2.5549000 | -0.3596000 |
| C | 2.6270000 | 3.6712000 | 6.4616000 |
| H | 2.3873000 | 3.4865000 | 7.3629000 |
| C | 3.7016000 | 3.0338000 | 5.8559000 |
| C | 4.0341000 | 3.2876000 | 4.5466000 |
| H | 4.7637000 | 2.8350000 | 4.1397000 |
| C | 1.9437000 | 3.4394000 | -0.8064000 |
| H | 2.0389000 | 3.6247000 | -1.7336000 |
| C | 2.2749000 | 4.8424000 | 4.4137000 |
| H | 1.7929000 | 5.4866000 | 3.9084000 |
| C | 0.8504000 | 2.2682000 | 0.9797000 |
| H | 0.1828000 | 1.6601000 | 1.2736000 |
| C | 3.6276000 | 4.4596000 | 2.4335000 |
| H | 4.5502000 | 4.1554000 | 2.2419000 |
| H | 3.5926000 | 5.4368000 | 2.2790000 |
| C | 2.7721000 | 4.0524000 | 0.1290000 |
| H | 3.4233000 | 4.6782000 | -0.1658000 |
| C | 1.9162000 | 4.5870000 | 5.7008000 |
| H | 1.1725000 | 5.0403000 | 6.0817000 |
| C | 1.7013000 | 2.8719000 | 1.9046000 |
| H | 1.6193000 | 2.6675000 | 2.8281000 |
| C | 2.6683000 | 3.7699000 | 1.4786000 |
| Br | 6.2393000 | 0.3228000 | 9.6006000 |

(*N*-4Ito-3Brpy)I halogen-bonded complex with pyridyl fragment

$E$ = -16931.86466560 a.u.

| Symbol | X | Y | Z |
|---|---|---|---|
| I | 6.3643000 | 8.2211000 | 9.8948000 |
| I | -0.3663000 | 6.9207000 | -1.6760000 |
| Br | 4.8144000 | 6.6237000 | 7.0331000 |
| N | 3.3058000 | 4.4269000 | 3.9256000 |
| C | 0.9101000 | 5.9821000 | -0.2863000 |
| C | 3.7298000 | 5.4945000 | 5.9859000 |
| C | 0.7919000 | 6.2995000 | 1.0523000 |
| C | 2.6332000 | 4.8272000 | 1.5630000 |
| C | 1.8742000 | 5.0856000 | -0.7217000 |
| C | 1.6679000 | 5.7289000 | 1.9716000 |
| C | 3.6314000 | 4.1822000 | 2.5037000 |
| C | 2.2429000 | 3.7810000 | 4.4740000 |
| C | 4.0444000 | 5.2789000 | 4.6581000 |
| C | 1.8959000 | 4.0009000 | 5.7709000 |
| C | 2.7270000 | 4.5065000 | 0.2110000 |
| C | 2.6433000 | 4.8700000 | 6.5557000 |
| H | 1.9511000 | 4.8700000 | -1.6436000 |
| H | 3.3830000 | 3.8837000 | -0.0779000 |
| H | 1.6025000 | 5.9616000 | 2.8908000 |
| H | 0.1183000 | 6.9025000 | 1.3439000 |
| H | 1.7375000 | 3.1728000 | 3.9471000 |
| H | 4.7821000 | 5.7314000 | 4.2630000 |
| H | 2.4100000 | 5.0300000 | 7.4629000 |
| H | 1.1418000 | 3.5586000 | 6.1418000 |
| H | 4.5354000 | 4.5398000 | 2.3116000 |
| H | 3.6494000 | 3.2062000 | 2.3371000 |

(*N*-4Ito-3Brpy)Br halogen-bonded complex with benzoyl fragment

*E* = -12585.13403570 a.u.

| Symbol | X | Y | Z |
|---|---|---|---|
| I | -0.2734000 | 1.5581000 | -1.7324000 |
| Br | 4.7500000 | 1.8527000 | 6.8895000 |
| N | 3.3147000 | 4.1848000 | 3.8469000 |
| C | 0.9812000 | 2.5549000 | -0.3596000 |
| C | 2.6270000 | 3.6712000 | 6.4616000 |
| H | 2.3873000 | 3.4865000 | 7.3629000 |
| C | 3.7016000 | 3.0338000 | 5.8559000 |
| C | 4.0341000 | 3.2876000 | 4.5466000 |
| H | 4.7637000 | 2.8350000 | 4.1397000 |
| C | 1.9437000 | 3.4394000 | -0.8064000 |
| H | 2.0389000 | 3.6247000 | -1.7336000 |
| C | 2.2749000 | 4.8424000 | 4.4137000 |
| H | 1.7929000 | 5.4866000 | 3.9084000 |
| C | 0.8504000 | 2.2682000 | 0.9797000 |
| H | 0.1828000 | 1.6601000 | 1.2736000 |
| C | 3.6276000 | 4.4596000 | 2.4335000 |
| H | 4.5502000 | 4.1554000 | 2.2419000 |
| H | 3.5926000 | 5.4368000 | 2.2790000 |
| C | 2.7721000 | 4.0524000 | 0.1290000 |
| H | 3.4233000 | 4.6782000 | -0.1658000 |
| C | 1.9162000 | 4.5870000 | 5.7008000 |
| H | 1.1725000 | 5.0403000 | 6.0817000 |
| C | 1.7013000 | 2.8719000 | 1.9046000 |
| H | 1.6193000 | 2.6675000 | 2.8281000 |
| C | 2.6683000 | 3.7699000 | 1.4786000 |
| Br | -3.4409000 | 0.3228000 | -3.4269000 |

(*N*-4Brto-3Brpy)Cl halogen-bonded complex with benzoyl fragment

*E* = -6125.13798437 a.u.

| Symbol | X | Y | Z |
|---|---|---|---|
| I | 0.6068000 | 10.5277000 | 13.0159000 |
| Br | -0.2313000 | 2.2237000 | 8.8453000 |
| N | 2.7761000 | 4.3454000 | 10.5823000 |
| C | 1.8224000 | 3.9119000 | 9.7290000 |
| H | 1.6711000 | 4.3546000 | 8.9253000 |
| C | 1.6380000 | 8.9465000 | 12.0748000 |
| C | 1.0777000 | 2.8038000 | 10.0686000 |
| C | 2.9256000 | 6.7973000 | 10.8556000 |
| C | 2.6493000 | 8.2713000 | 12.7476000 |
| H | 2.8962000 | 8.5363000 | 13.6042000 |
| C | 3.0068000 | 3.7205000 | 11.7561000 |
| H | 3.6721000 | 4.0342000 | 12.3253000 |
| C | 1.2853000 | 8.5716000 | 10.7810000 |
| H | 0.6227000 | 9.0360000 | 10.3227000 |
| C | 3.2904000 | 7.2004000 | 12.1393000 |
| H | 3.9671000 | 6.7496000 | 12.5908000 |
| C | 1.9301000 | 7.4998000 | 10.1814000 |
| H | 1.6946000 | 7.2471000 | 9.3179000 |
| C | 2.2614000 | 2.6220000 | 12.1131000 |
| H | 2.4214000 | 2.1980000 | 12.9251000 |
| C | 3.5527000 | 5.5804000 | 10.2268000 |
| H | 3.5709000 | 5.6861000 | 9.2628000 |
| H | 4.4671000 | 5.4894000 | 10.5374000 |
| C | 1.2756000 | 2.1452000 | 11.2707000 |
| H | 0.7615000 | 1.4060000 | 11.5034000 |
| Cl | -1.2470000 | 12.8744000 | 14.3432000 |

(*N*-4Ito-3Brpy)I halogen-bonded complex with benzoyl fragment

*E* = -16931.84384990 a.u.

| Symbol | X | Y | Z |
|---|---|---|---|
| I | -0.3663000 | 6.9207000 | -1.6760000 |
| Br | 4.8144000 | 6.6237000 | 7.0331000 |
| N | 3.3058000 | 4.4269000 | 3.9256000 |
| C | 0.9101000 | 5.9821000 | -0.2863000 |
| C | 3.7298000 | 5.4945000 | 5.9859000 |
| C | 0.7919000 | 6.2995000 | 1.0523000 |
| C | 2.6332000 | 4.8272000 | 1.5630000 |
| C | 1.8742000 | 5.0856000 | -0.7217000 |
| C | 1.6679000 | 5.7289000 | 1.9716000 |
| C | 3.6314000 | 4.1822000 | 2.5037000 |
| C | 2.2429000 | 3.7810000 | 4.4740000 |
| C | 4.0444000 | 5.2789000 | 4.6581000 |
| C | 1.8959000 | 4.0009000 | 5.7709000 |
| C | 2.7270000 | 4.5065000 | 0.2110000 |
| C | 2.6433000 | 4.8700000 | 6.5557000 |
| H | 1.9511000 | 4.8700000 | -1.6436000 |
| H | 3.3830000 | 3.8837000 | -0.0779000 |
| H | 1.6025000 | 5.9616000 | 2.8908000 |
| H | 0.1183000 | 6.9025000 | 1.3439000 |
| H | 1.7375000 | 3.1728000 | 3.9471000 |
| H | 4.7821000 | 5.7314000 | 4.2630000 |
| H | 2.4100000 | 5.0300000 | 7.4629000 |
| H | 1.1418000 | 3.5586000 | 6.1418000 |
| H | 4.5354000 | 4.5398000 | 2.3116000 |
| H | 3.6494000 | 3.2062000 | 2.3371000 |
| I | -3.4361000 | 8.2211000 | -3.5446000 |

(*N*-4Brto-3Clpy)Br halogen-bonded complex with benzoyl fragment

*E* = -6125.43583120 a.u.

| Symbol | X | Y | Z |
|---|---|---|---|
| Br | 4.9758000 | 1.5378000 | 8.1542000 |
| Cl | 0.1174000 | 1.7831000 | -0.1845000 |
| N | 1.5045000 | 4.0705000 | 2.7015000 |
| C | 3.8474000 | 2.4349000 | 6.8988000 |
| C | 2.1756000 | 3.6605000 | 5.0653000 |
| C | 0.7805000 | 3.1539000 | 2.0283000 |
| H | 0.0735000 | 2.6870000 | 2.4580000 |
| C | 2.8988000 | 3.3326000 | 7.3528000 |
| H | 2.8209000 | 3.5286000 | 8.2791000 |
| C | 2.0628000 | 3.9401000 | 6.4272000 |
| H | 1.4039000 | 4.5566000 | 6.7252000 |
| C | 2.1191000 | 3.5671000 | 0.0726000 |
| H | 2.3292000 | 3.3884000 | -0.8365000 |
| C | 1.0831000 | 2.9038000 | 0.7034000 |
| C | 3.9842000 | 2.1406000 | 5.5611000 |
| H | 4.6473000 | 1.5254000 | 5.2700000 |
| C | 2.5124000 | 4.7476000 | 2.1091000 |
| H | 2.9958000 | 5.4011000 | 2.6010000 |
| C | 1.1962000 | 4.3426000 | 4.1264000 |
| H | 0.2797000 | 4.0267000 | 4.3268000 |
| H | 1.2222000 | 5.3197000 | 4.2836000 |
| C | 3.1398000 | 2.7538000 | 4.6393000 |
| H | 3.2224000 | 2.5513000 | 3.7148000 |
| C | 2.8418000 | 4.4959000 | 0.7982000 |
| H | 3.5650000 | 4.9597000 | 0.3917000 |
| Br | 8.0775000 | 0.2679000 | 9.8666000 |

(*N*-4Brto-3Clpy)I halogen-bonded complex with benzoyl fragment

*E* = -10471.71864160 a.u.

| Symbol | X | Y | Z |
|---|---|---|---|
| Br | 5.1798000 | 6.7598000 | 8.2923000 |
| Cl | 0.1188000 | 6.5104000 | -0.1251000 |
| N | 1.6030000 | 4.3681000 | 2.8229000 |
| C | 0.8522000 | 5.2360000 | 2.1195000 |
| H | 0.1426000 | 5.7049000 | 2.5419000 |
| C | 2.3061000 | 4.7609000 | 5.1867000 |
| C | 4.0213000 | 5.9226000 | 7.0339000 |
| C | 3.0885000 | 4.9951000 | 7.4664000 |
| H | 3.0333000 | 4.7583000 | 8.3844000 |
| C | 3.2529000 | 5.6943000 | 4.7769000 |
| H | 3.3086000 | 5.9398000 | 3.8607000 |
| C | 1.1187000 | 5.4383000 | 0.7875000 |
| C | 2.2327000 | 4.4151000 | 6.5361000 |
| H | 1.5913000 | 3.7762000 | 6.8227000 |
| C | 1.3083000 | 4.1213000 | 4.2550000 |
| H | 1.2977000 | 3.1448000 | 4.4177000 |
| H | 0.4054000 | 4.4712000 | 4.4621000 |
| C | 2.6239000 | 3.6966000 | 2.2490000 |
| H | 3.1369000 | 3.0832000 | 2.7618000 |
| C | 2.1680000 | 4.7802000 | 0.1735000 |
| H | 2.3664000 | 4.9301000 | -0.7435000 |
| C | 2.9227000 | 3.9006000 | 0.9236000 |
| H | 3.6476000 | 3.4352000 | 0.5233000 |
| C | 4.1151000 | 6.2667000 | 5.6979000 |
| H | 4.7684000 | 6.8946000 | 5.4125000 |
| I | 8.1951000 | 8.0491000 | 10.1753000 |

(*N*-4Brto-3Clpy)Br halogen-bonded complex with pyridyl fragment

*E* = -6125.16507001 a.u.

| Symbol | X | Y | Z |
|---|---|---|---|
| Br | 4.9758000 | 1.5378000 | 8.1542000 |
| Cl | 0.1174000 | 1.7831000 | -0.1845000 |
| N | 1.5045000 | 4.0705000 | 2.7015000 |
| C | 3.8474000 | 2.4349000 | 6.8988000 |
| C | 2.1756000 | 3.6605000 | 5.0653000 |
| C | 0.7805000 | 3.1539000 | 2.0283000 |
| H | 0.0735000 | 2.6870000 | 2.4580000 |
| C | 2.8988000 | 3.3326000 | 7.3528000 |
| H | 2.8209000 | 3.5286000 | 8.2791000 |
| C | 2.0628000 | 3.9401000 | 6.4272000 |
| H | 1.4039000 | 4.5566000 | 6.7252000 |
| C | 2.1191000 | 3.5671000 | 0.0726000 |
| H | 2.3292000 | 3.3884000 | -0.8365000 |
| C | 1.0831000 | 2.9038000 | 0.7034000 |
| C | 3.9842000 | 2.1406000 | 5.5611000 |
| H | 4.6473000 | 1.5254000 | 5.2700000 |
| C | 2.5124000 | 4.7476000 | 2.1091000 |
| H | 2.9958000 | 5.4011000 | 2.6010000 |
| C | 1.1962000 | 4.3426000 | 4.1264000 |
| H | 0.2797000 | 4.0267000 | 4.3268000 |
| H | 1.2222000 | 5.3197000 | 4.2836000 |
| C | 3.1398000 | 2.7538000 | 4.6393000 |
| H | 3.2224000 | 2.5513000 | 3.7148000 |
| C | 2.8418000 | 4.4959000 | 0.7982000 |
| H | 3.5650000 | 4.9597000 | 0.3917000 |
| Br | -1.3262000 | 0.2679000 | -3.0894000 |
